# Performance of Deep Learning in Classifying Age-Related Macular Degeneration From Images: Systematic Review and Meta-Analysis

**DOI:** 10.2196/97174

**Published:** 2026-06-15

**Authors:** Yu Zhu, Yue Niu, Shangye Sun, Wei Liu, Ying Dou, Yu Guo

**Affiliations:** 1 Department of Ophthalmology, Jilin Province FAW General Hospital, Changchun, 130011, China; 2 Department of Human Resources, Jilin Province FAW General Hospital, Changchun, China; 3 Department of CT, Jilin Province FAW General Hospital, Changchun, China; 4 Department of Otolaryngology, Jilin Province FAW General Hospital, 2643 Dongfeng StreetChangchun, Jilin Province, 130011, China, 86 15948784509

**Keywords:** age-related macular degeneration, deep learning, artificial intelligence, optical coherence tomography, meta-analysis

## Abstract

**Background:**

Age-related macular degeneration (AMD) is a leading cause of irreversible blindness worldwide. Retinal imaging and deep learning (DL) may support scalable screening, but deployment requires evidence on pooled performance. This is important because missed neovascular disease may delay treatment, whereas excessive false positives may overload referral pathways.

**Objective:**

This study aimed to compare the diagnostic performance of DL algorithms with ophthalmologists for detecting AMD and differentiating wet AMD (wAMD) from dry AMD (dAMD) and to identify factors that influence DL performance.

**Methods:**

PubMed, Embase, Web of Science, and the Cochrane Library were searched through October 5, 2025, and updated on April 19, 2026. Eligible studies applied DL to classify AMD from normal retinas or wAMD from dAMD using retinal images. Two reviewers (MHT and XL) independently extracted data and assessed risk of bias using the Prediction model Risk Of Bias Assessment Tool for Artificial Intelligence (PROBAST+AI) tool. Pooled sensitivity, specificity, accuracy, and area under the curve were estimated using bivariate random-effects models. Clinician comparisons were stratified by experience (junior vs senior). Small-study effects were assessed via Deeks’ funnel plot asymmetry test. Evidence certainty was appraised using the Grading of Recommendations, Assessment, Development, and Evaluation framework. The protocol was registered in the International Prospective Register of Systematic Reviews (PROSPERO; CRD420251243276).

**Results:**

Overall, 28 studies were included, comprising 77,485 samples for AMD detection and 28,705 samples for wAMD versus dAMD classification. For AMD detection, DL achieved a pooled sensitivity of 0.98 (95% CI 0.96‐0.99; prediction interval [PI] 0.95‐0.99), specificity of 0.98 (95% CI 0.95‐0.99; PI 0.95‐0.99), accuracy of 0.97 (95% CI 0.96‐0.99), and area under the curve of 1.00 (95% CI 0.99‐1.00). For wAMD versus dAMD, DL showed sensitivity of 0.95 (95% CI 0.91‐0.97; PI 0.89‐0.97), specificity of 0.95 (95% CI 0.93‐0.97; PI 0.92‐0.97), accuracy of 0.95 (95% CI 0.92‐0.97), and area under the curve of 0.99 (95% CI 0.97‐0.99). DL showed higher sensitivity than senior ophthalmologists for AMD (0.98 vs 0.75; *P*<.001) and higher specificity and accuracy than junior ophthalmologists for wAMD classification. Optical coherence tomography–based models performed more consistently than color fundus photography or multimodal models. Evidence certainty was moderate.

**Conclusions:**

Compared with ophthalmologists, DL algorithms demonstrated superior and more balanced diagnostic performance in the available head-to-head evidence, potentially providing a consistent decision-support baseline that mitigates human threshold-dependent trade-offs. However, high heterogeneity, wide PIs, predominantly retrospective designs, and possible performance inflation from internal validation mean that these relative performance findings remain preliminary rather than deployment ready. DL should be viewed as a triage adjunct requiring local calibration, not an autonomous diagnostic replacement. Prospective, multicenter, patient-level external validation with prespecified human comparison arms is required.

## Introduction

Age-related macular degeneration (AMD) remains a leading cause of irreversible blindness in older individuals globally [1]. Clinically, the disease is classified into dry AMD (dAMD), characterized by the progressive accumulation of drusen and geographic atrophy, and wet AMD (wAMD), which involves rapid vision loss due to macular neovascularization [2]. As the global population ages, the prevalence of AMD is projected to rise significantly; recent estimates indicate that the number of individuals with AMD-related vision impairment will increase from 8.06 million in 2021 to approximately 21.34 million by 2050 [1]. Consequently, early and accurate diagnosis is paramount. Timely detection allows for appropriate intervention, which is critical for slowing disease progression, preserving visual function, and improving overall patient prognosis.

Conventionally, color fundus photography (CFP) and optical coherence tomography (OCT) serve as the cornerstones for AMD screening and diagnosis. However, reliance on these modalities presents distinct challenges. CFP is frequently limited by image quality; issues such as media opacities or small pupils can render images ungradable, with rates as high as 47.6% in some screening contexts, and CFP often lacks sensitivity for detecting subtle early-stage structural changes or neovascular activity [3,4]. Conversely, while OCT offers high gradability (up to 97.7%) and detailed cross-sectional visualization, it is constrained by a limited field of view and reduced efficacy in identifying pigmentary abnormalities compared to CFP [3,4]. Beyond these technical constraints, the manual interpretation of vast imaging datasets is inherently labor-intensive, subjective, and prone to interobserver variability, creating a scalability bottleneck for population-wide screening.

In response to these challenges, deep learning (DL) algorithms using OCT, CFP, or multimodal imaging have emerged as a transformative approach, offering the potential for automated, high-throughput classification [5,6]. While these algorithms demonstrate theoretical superiority in efficiency and feature extraction, the current literature reveals substantial heterogeneity in performance outcomes [7,8]. Discrepancies regarding model generalization to real-world settings and the comparative performance of DL algorithms against ophthalmologists remain unresolved [7,8]. Two pivotal questions persist: First, how does the diagnostic performance of DL models quantitatively compare against ophthalmologists of varying expertise? Second, what factors, such as imaging modality, type of validation, database source, study centers, and unit of analysis, influence DL performance? Existing literature offers fragmented and sometimes contradictory insights, lacking a comprehensive quantitative synthesis.

Several previous meta-analyses have evaluated DL performance in AMD diagnosis. Leng et al [9] reported a pooled sensitivity of 94% and specificity of 97% for convolutional neural network–based algorithms, while Chen et al [10] highlighted the overall superiority of artificial intelligence (AI) over retinal specialists. However, these prior reviews have notable limitations: they did not stratify human-AI comparisons by clinician experience level, used conventional bias assessment tools rather than the recently developed Prediction model Risk Of Bias Assessment Tool for Artificial Intelligence (PROBAST+AI) instrument [11], and did not separately evaluate the clinically critical task of differentiating wAMD from dAMD. Moreover, the rapid advancement of DL architectures, particularly vision transformers, necessitates an updated quantitative synthesis incorporating the latest evidence.

Importantly, our review was designed to address several evidence gaps that were not fully covered in previous meta-analyses. First, instead of evaluating DL algorithms in isolation, we directly compared DL performance with ophthalmologists and further stratified these comparisons by clinician experience level. This is clinically relevant because screening and referral decisions are often made by clinicians with different levels of expertise. Second, we separately evaluated the classification of wAMD versus dAMD, a task with immediate therapeutic implications because delayed recognition of wAMD may postpone anti–vascular endothelial growth factor treatment. Third, we incorporated PROBAST+AI, a recently developed tool tailored to prediction models using AI, thereby providing a more AI-specific assessment of bias than conventional quality appraisal tools [12]. Fourth, we examined prediction intervals (PIs), validation strategy, imaging modality, and other sources of heterogeneity to move beyond average pooled performance and assess the likely robustness of DL algorithms across clinical settings. These features make the current review not only an update of the evidence base, but also a more deployment-oriented synthesis of the clinical value and limitations of DL for AMD image classification.

Therefore, this systematic review and meta-analysis addressed these clinically relevant and deployment-oriented evidence gaps. Its objective was to evaluate the diagnostic performance of DL algorithms compared with ophthalmologists of varying experience levels for detecting AMD and differentiating its subtypes (wAMD vs dAMD), and to assess potential factors influencing DL diagnostic performance through subgroup analyses and meta-regressions.

## Methods

### Overview

This systematic review and meta-analysis was conducted in strict accordance with the PRISMA-DTA (Preferred Reporting Items for Systematic Reviews and Meta-Analyses of Diagnostic Test Accuracy) guidelines [13], with the specific reporting items detailed in Table S1 in Multimedia Appendix 1. The abstract was reported in accordance with the PRISMA (Preferred Reporting Items for Systematic Reviews and Meta-Analyses) 2020 for abstracts checklist, as shown in Table S2 in Multimedia Appendix 1. This systematic review and meta-analysis adhered to the preregistered protocol (PROSPERO [International Prospective Register of Systematic Reviews] CRD420251243276). At the request of reviewers, PIs for sensitivity and specificity were calculated as an additional analysis to provide estimates of expected performance in new clinical settings [13].

### Search Strategy

A comprehensive literature search was conducted across PubMed, Embase, Web of Science, and the Cochrane Library databases, with coverage extending through April 19, 2026. The initial search was conducted through October 5, 2025, and subsequently updated on April 19, 2026, to capture any recently published studies. Two independent reviewers (YZ and YN) performed the preliminary screening of titles and abstracts, followed by a full-text assessment. The search strategy used a combination of free-text terms and Medical Subject Headings focusing on four distinct domains: AMD-related terminologies (eg, “Macular Degeneration”), DL concepts (eg, “Artificial Intelligence” and “Deep Learning”), imaging modalities (eg, “Optical Coherence Tomography” and “fundus photograph”), and diagnostic performance metrics (eg, “sensitivity” and “specificity”). No restrictions regarding language or publication year were applied during the initial retrieval. To ensure exhaustiveness, reference lists of included studies and relevant meta-analyses were manually scrutinized for additional literature. Detailed search queries were provided in Table S3 in Multimedia Appendix 1.

### Eligibility Criteria

The study inclusion adhered to the Patient, Index test, Target condition, Reference standard, Outcome, and Setting (PITROS) framework, which is detailed in Table 1. To ensure the analysis focused on diagnostic test accuracy, several exclusion criteria were systematically applied. We excluded publications with clearly irrelevant titles and abstracts, and specific noneligible study types, including reviews, cross-sectional surveys, case reports, conference abstracts, meta-analyses, letters, and studies with unavailable full texts. Furthermore, studies were excluded if their primary aim was not the classification of AMD versus Normal or wAMD versus dAMD, or if they lacked sufficient data to extract or calculate a 2×2 contingency table (true positives [TPs], false positives [FPs], false negatives [FNs], and true negatives [TNs]). The screening was performed independently by 2 reviewers (YZ and SYS). Any discrepancies were resolved through discussion or, if necessary, by consultation with a third senior reviewer (YG) to reach a final consensus.

**Table 1.** Summary of inclusion criteria using the PITROS^a^ framework.

Criteria	Details
Participants (P)	Adults undergoing retinal imaging (CFP^b^ or OCT^c^) with confirmed ocular status (normal, dAMD^d^, or wAMD^e^) based on clinical diagnosis or standard imaging protocols.
Index test (I)	DL^f^ algorithms using retinal images (CFP, OCT, or both) for automated diagnosis or classification.
Target conditions (T)	The study addressed two classification tasks: first, AMD^g^ versus normal, comparing confirmed AMD cases (positive) against healthy controls (negative); and second, wAMD versus dAMD, distinguishing exudative or neovascular AMD (positive) from nonexudative or atrophic AMD (negative).
Reference standard (R)	Clinical diagnosis by ophthalmologists based on multimodal imaging (eg, CFP, OCT, and fluorescein angiography) and/or longitudinal follow-up.
Outcomes (O)	Diagnostic performance metrics, including sensitivity, specificity, accuracy, and AUC^h^. Data extraction focused on contingency tables (TP^i^, FP^j^, FN^k^, and TN^l^).
Settings (S)	Retrospective or prospective studies using single-center, multicenter clinical datasets, or public databases (eg, AREDS^m^ and Kaggle).

a PITROS: Patient, Index test, Target condition, Reference standard, Outcome, and Setting.

b CFP: color fundus photography.

c OCT: optical coherence tomography.

d dAMD: dry age-related macular degeneration.

e wAMD: wet age-related macular degeneration.

f DL: deep learning.

g AMD: age-related macular degeneration.

h AUC: area under the curve.

i TP: true positive.

j FP: false positive.

k FN: false negative.

l TN: true negative.

m AREDS: Age-Related Eye Disease Study.

### Quality Assessment and Certainty of Evidence

The methodological quality and risk of bias of the included studies were assessed using the PROBAST+AI tool [11], an updated version replacing PROBAST 2019. This tool evaluates two distinct phases, model development and model evaluation, across seven domains each, encompassing participants, predictors, outcomes, and analysis. Each domain is judged as having a low, high, or unclear risk of bias based on a series of tailored signaling questions. These questions are rated as “yes,” “probably yes,” “probably no,” “no,” “no information,” or “not applicable”. The complete set of signaling questions and detailed ratings were provided in Tables S4 and S5 in Multimedia Appendix 1. To ensure objectivity and accuracy, 2 reviewers (YZ and WL) independently performed this assessment for all included studies.

To appraise the certainty of the evidence for the pooled sensitivity, specificity, and diagnostic accuracy, we used the Grading of Recommendations, Assessment, Development, and Evaluations (GRADE) framework for diagnostic studies. This approach focuses on five key domains: risk of bias, indirectness, inconsistency, imprecision, and small-study effects [13]. The GRADE summary of findings table was formatted according to the diagnostic test accuracy template recommended by the GRADE working group and used a pretest probability of 20% for expected results per 1000 tested. The full GRADE assessment criteria and the final judgments for each outcome were detailed in Table S6 in Multimedia Appendix 1.

### Data Extraction

Two reviewers (YZ and YD) independently performed data extraction from the full-text articles, and disagreements were resolved by discussion with a third reviewer (YG). Extracted information included study design, patient or sample size, imaging modality, data source, validation design, reference standard, target condition, AI architecture, diagnostic contingency data or reconstructed diagnostic data, ophthalmologist experience level when available, and risk-of-bias and certainty judgments. The data extraction tables are included in the paper.

As most studies did not report the full binary diagnostic contingency table (2×2 table), we used an indirect derivation approach. Specifically, TP, FP, FN, and TN values were extracted by merging categories from multiclass confusion matrices (three- or four-class tables) reported in the included studies. In a few cases where such matrices were unavailable, the values were indirectly calculated using reported sensitivity, specificity, AMD sample size, and total sample size. When studies provided multiple, nonoverlapping validation sets, the corresponding contingency tables were assumed independent, and all were extracted. However, when multiple DL algorithms were presented within a single study, only the major model defined by the authors was extracted to avoid patient overlap in data pooling. To ensure the independence of data in our meta-analyses and avoid double counting, this approach guaranteed that the same patient sample was not counted more than once.

### Outcome Measures

The primary outcome measures were pooled sensitivity, specificity, accuracy, and area under the curve (AUC). Sensitivity and specificity describe threshold-dependent diagnostic performance, accuracy summarizes the proportion of correctly classified samples in the analyzed dataset and may be affected by class balance, and AUC reflects threshold-independent discrimination across possible decision thresholds. These metrics were therefore interpreted as complementary rather than interchangeable indicators of model behavior.

### Statistical Analysis

Considering the inherent heterogeneity anticipated among studies, a bivariate random-effects model was used to pool the sensitivity, specificity, and AUC values for both DL algorithms and ophthalmologists [14]. For the diagnostic accuracy metric, a generalized linear mixed model with a random-effects framework was used following a rlogit transformation. A 2-sample Z-test was used to compare the differences in pooled sensitivity, specificity, diagnostic accuracy, and AUC, with statistical significance defined as a *P* value <.05. It is important to note that this bivariate model uses restricted maximum likelihood estimation, which differs fundamentally from the DerSimonian-Laird approach used in standard pairwise meta-analyses. While the Hartung-Knapp-Sidik-Jonkman adjustment is recommended for DL-based analyses to reduce false positives [15], it is not directly applicable to the bivariate diagnostic framework, as the restricted maximum likelihood–based bivariate model already provides more accurate variance estimation that inherently guards against inflated type I error rates. To characterize the distribution of true effects across different populations and settings, 95% PIs were calculated where a sufficient number of studies (≥3) were available, complementing the confidence intervals for the pooled average effects [16]. While confidence intervals quantify the precision of the average effect, PIs estimate the range within which the true diagnostic performance of a future study is expected to fall.

For DL algorithm outcomes demonstrating substantial heterogeneity, a bivariate boxplot and multivariable meta-regression were performed to explore potential sources. As prespecified, subgroup analyses were conducted based on imaging modality (OCT vs CFP vs multimodal), with between-subgroup differences compared and visualized using violin plots. The potential clinical impact of the DL algorithms was assessed using a Fagan nomogram. Small-study effects were evaluated using Deeks’ funnel plot asymmetry test, with a *P* value <.10 indicating potential asymmetry [17]. It should be noted that funnel plot asymmetry can arise from multiple sources beyond small-study effects, including differences in study quality, true heterogeneity, and chance [17,18]. All statistical analyses were performed using Stata 15.1 (StataCorp LLC, with the *midas* and *metadta* packages) and R (version 4.5.1; R Core Team, using the *ggplot2* and *tidyverse* packages). All statistical tests were 2-tailed.

### Use of Large Language Models

During the preparation of this work, we used OpenAI Codex (GPT-5) to assist with text generation, proofreading and editing, summarizing text, formulation of conclusions, translation, and reformatting under full human supervision. The tool was not used to make eligibility decisions, extract data, perform statistical analyses, or draw independent scientific conclusions. After using this tool, we reviewed and edited the content as needed and took full responsibility for the content of the publication.

## Results

### Study Selection

**Figure 1.**
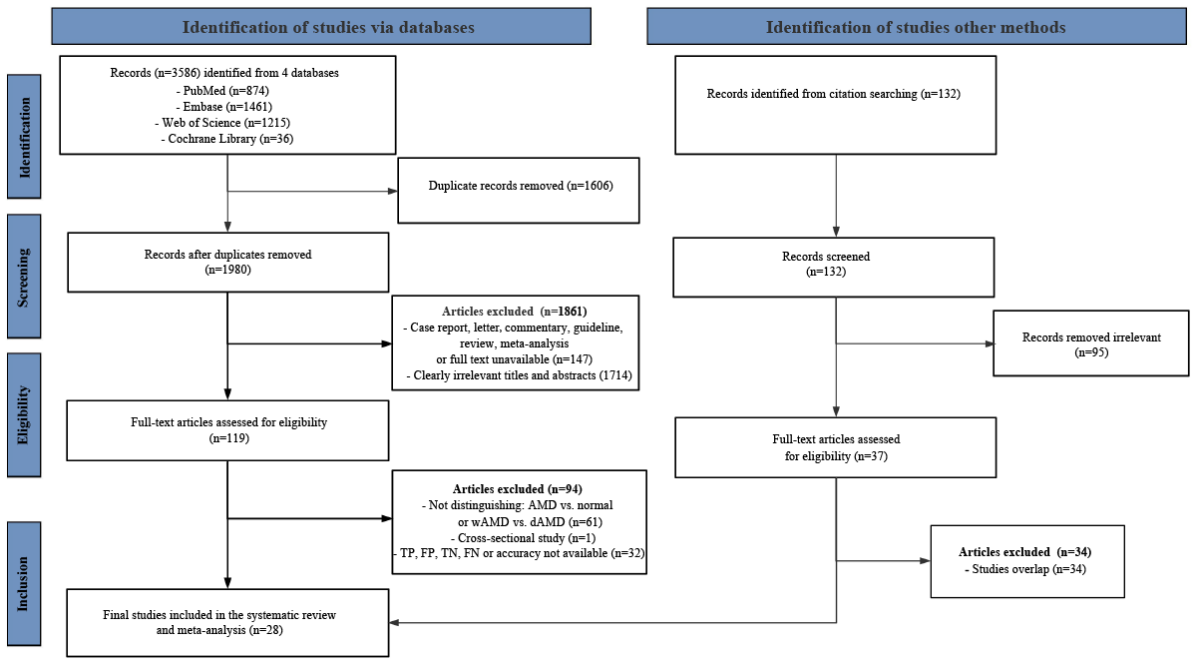
PRISMA (Preferred Reporting Items for Systematic Reviews and Meta-Analyses) flow diagram illustrating the study selection process for the systematic review and meta-analysis. AMD: age-related macular degeneration; dAMD: dry age-related macular degeneration; FN: false negative; FP: false positive; TN: true negative; TP: true positive; wAMD: wet age-related macular degeneration.

The initial database search identified 3586 potentially relevant records. After removing duplicates, 1980 unique records underwent title and abstract screening. During this phase, 1861 records were excluded due to obvious irrelevance or ineligible publication types (eg, reviews and conference abstracts). Subsequently, 119 full-text articles were assessed for eligibility. Following a detailed review, 61 studies were excluded as they did not primarily focus on distinguishing AMD from normal retinas or discriminating wAMD from dAMD. One cross-sectional study and 32 studies lacking sufficient or complete diagnostic data (TP, FP, FN, and TN) were further excluded. An additional 3 articles identified from other nondatabase sources (eg, reference lists) were also included [19-21]. Consequently, 28 studies [19-46] met all predefined inclusion criteria and were included in the meta-analysis. The study selection process followed the PRISMA guidelines, as shown in Figure 1.

### Study Characteristics

A total of 27 studies [19-36,38-46], comprising a validation set of 77,485 samples, targeted the classification of AMD versus normal. Among these, diagnostic performance data were available for junior ophthalmologists in 1 study [24], and for senior ophthalmologists in 3 studies [20,24,36]. Ten studies were based on OCT imaging [23,25-28,32,34,35,40,44], 12 on CFP [19-21,24,29-31,33,36,38,39,41], and 4 on multimodal (OCT+CFP) inputs [42,43,45,46]. Detailed study, patient, and technical characteristics were presented in Table 2 and Tables S7-S8 in Multimedia Appendix 1.

For the classification of wAMD versus dAMD, 16 studies (validation set: 28,705 samples) were included [19,23-29,31,33,37,41-44,46]. Data for head-to-head comparisons with ophthalmologists were available from two studies each for junior and senior practitioners [19,24], enabling stratified analysis by experience level. Six studies used OCT [23,25-28,44], 7 used CFP [19,24,29,31,33,37,41], and 3 used multimodal imaging [42,43,46]. Corresponding detailed characteristics were provided in Table 2 and Tables S9-S10 in Multimedia Appendix 1. The complete diagnostic data for ophthalmologists were presented in Table S11 in Multimedia Appendix 1.

**Table 2.** Study and patient characteristics of the included studies.

Author	Year	Country	Study design	Analysis	Reference standard	Target condition	Number of total sample size			Number of positive sample size
							Training	IV^a^	EV^b^	
Abdelhalim et al [22]	2025	Multiple countries	Retrospective	IB^c^	—^d^	AMD^e^ versus normal	1011	127	—	Training: 589 IV: 74
Bao et al [24]	2025	China	Retrospective	IB	—		894			
						AMD versus normal		IV1: 223	EV1: 1395 EV2: 59	Training: 406 IV1: 101 EV1: 194 EV2: 40
						wAMD^f^ versus dAMD^g^		IV1: 92	EV1: 136 EV2: 40	Training: 267 IV1: 60 EV1: 36 EV2: 21
Durmaz Engin et al [28]	2025	NA	Retrospective	IB	Expert consensus	AMD versus normal	1200	300	—	Training: 800 IV: 200
Zhen et al [46]	2025	China	Retrospective	PB^h^	—		Training 1: 664		—	
						AMD versus normal		134		Training 1: 509 IV: 101
						wAMD versus dAMD		101		Training: 386 IV: 75
Alenezi et al [23]	2024	NA	Retrospective	IB	Expert consensus		554		—	
						AMD versus normal		554		Training: 367 IV: 367
						wAMD versus dAMD		361		Training: 173 IV: 173
García-Floriano et al [30]	2024	Multiple countries	Retrospective	IB	Expert consensus	AMD versus normal	250	250	22	Training: 128 IV: 128 EV: 11
Le et al [33]	2024	Multiple countries	Retrospective	IB	Expert consensus		2359			
						AMD versus normal		2359	750	Training: 1144 IV: 1144 EV: 500
						wAMD versus dAMD		1095	440	Training: 599 IV: 588 EV: 239
Oliveira et al [36]	2024	Multiple countries	Retrospective	IB	Expert consensus	AMD versus normal	6896	210	80	Training: 275 IV: 105 EV: 40
Wan et al [41]	2024	China	Retrospective	IB	Clinical classification manifestations and expert consensus		516			
						AMD versus normal		129	100	Training: 312 IV: 78 EV: 60
						wAMD versus dAMD		78	60	Training: 214 IV: 53 EV: 41
Yusufoglu et al [44]	2024	Turkey	Retrospective	IB	—				—	
						AMD versus normal	Training 1: 1622 Training 2: 2240	IV1: 347 IV2: 491		Training 1: NA IV1: 214 Training 2: NA IV2: 248
						wAMD versus dAMD	1622	213		Training 1: NA IV1: 110
Celebi et al [26]	2023	Turkey	Retrospective	IB	Expert consensus		Training 1: 4067 Training 2: 59,139		—	
						AMD versus normal		IV1: 1741 IV2: 25,345		Training 1: 2577 IV1: 1103 Training 2: 40,544 IV2: 17,375
						wAMD versus dAMD		IV1: 1085 IV2: 17,369		Training 1: 812 IV1: 345 Training 2: 26,219 IV2: 11,234
El-Den et al [29]	2023	Multiple countries	Retrospective	IB	—		605		—	
						AMD versus normal		128		Training: 454 IV: 98
						wAMD versus dAMD		97		Training: 151 IV: 32
Leingang et al [35]	2023	Multicountries	Retrospective	PB	Expert consensus	AMD versus normal	1733	96	—	Training: 1620 IV: 90
Chen et al [27]	2022	China	Retrospective	EB^i^	Expert consensus		612			
						AMD versus normal		153	214	Training: 356 IV: 89 EV: 162
						wAMD versus dAMD		87	143	Training: 189 IV: 47 EV: 96
He et al [32]	2022	China	Retrospective	IB^c^	—	AMD versus normal	77,568	750	2130	Training: 36,656 IV: 500 EV: 723
Skevas et al [21]	2022	Germany	Prospective	PB^h^	Expert consensus	AMD versus normal	—	—	598	EV: 69
Wang et al [42]	2022	China	Retrospective	EB	Expert consensus		—		—	
						AMD versus normal		91		Training: NA IV: 71
						wAMD versus dAMD		71		Training: NA IV: 33
Tak et al [37]	2021	United States	Retrospective	IB	Expert consensus	wAMD versus dAMD	350	72	—	Training: NA IV: 28
Takhchidi et al [38]	2021	Russia	Retrospective	IB	Expert consensus	AMD versus normal	994	206	—	Training: 475 IV: 100
Thomas et al [40]	2021	India	Retrospective	IB	Expert consensus	AMD versus normal	87,264	750	30	Training: 41,238 IV: 500 EV: 15
Heo et al [19]	2020	Korea	Retrospective	IB	Expert consensus		279	279	—	
						AMD versus normal				Training: 191 IV: 191
						wAMD versus dAMD				Training: 99 IV: 99
Zapata et al [45]	2020	Multicountries	Retrospective	IB	Expert consensus	AMD versus normal	7949	2208	—	Training: NA IV: 1082
Bhatia et al [25]	2019	Multiple countries	Retrospective	EB	—		—	—		
						AMD versus normal			EV1:98 EV1:75	EV1: 48 EV2: 50
						wAMD versus dAMD			EV2:50	EV2: 25
Matsuba et al [20]	2019	Japan	Retrospective	IB	Expert consensus	AMD versus normal	5000	111	—	Training: 870 IV: 42
Yoo et al [43]	2019	Korea	Retrospective	IB	Pathological examination					
						AMD versus normal	Training 1: 2100	IV1: 900	EV2: 83	Training 1: 1400 IV1: 600 EV2: 48
						wAMD versus dAMD	—	—	EV2:48	EV2: 36
Grassmann et al [31]	2018	Multiple countries	Prospective	IB	Expert consensus		83,653			
						AMD versus normal		11,618	1677	Training: 53,375 IV: 7571 EV: 220
						wAMD versus dAMD		6631	123	Training: 9357 IV: 1432 EV: 4
Tan et al [39]	2018	Multicountries	Retrospective	IB	—	AMD versus normal	1110	1100	—	Training: 708 IV: 700
Lee et al [34]	2017	United States	Retrospective	IB	Expert consensus	AMD versus normal	80,839	20,163	—	Training: 41,074 IV: 11,616

a IV: internal validation.

b EV: external validation.

c IB: image-based.

d NA: not available.

e AMD: age-related macular degeneration.

f wAMD: wet age-related macular degeneration.

g dAMD: dry age-related macular degeneration.

h PB: patient-based.

i EB: eye-based.

### Quality Assessment and GRADE Certainty

**Figure 2.**
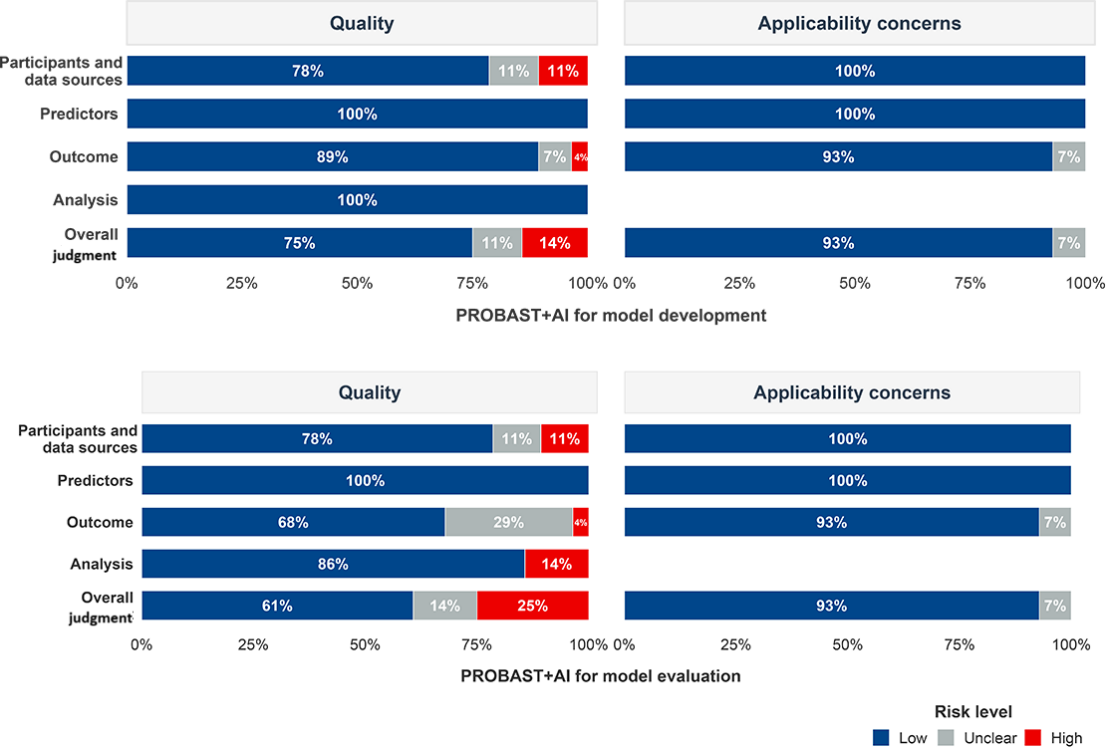
Risk of bias and applicability concerns of the included studies regarding model development and model evaluation domains using the Prediction model Risk Of Bias ASsessment Tool for artificial intelligence tools. The colors represent the proportion of studies with low, high, or unclear risk. PROBAST+AI: Prediction model Risk Of Bias ASsessment Tool for artificial intelligence.

The risk of bias and applicability concerns, as assessed by the PROBAST+AI tool, were summarized in Figure 2 and Tables S4-S5 in Multimedia Appendix 1. For the model development phase, 14% (4/28) of studies were judged to have a high overall risk of bias regarding quality [23,25,28,30], while none (0/28) raised high applicability concerns. For the model validation or testing phase, 25% (7/28) of studies were rated as having a high overall risk of bias [23,25,28,30,35,37,40], again with no studies (0/28) presenting high applicability concerns. Overall, the proportion of high-risk ratings was low, with most domains assessed as low risk, indicating an acceptable overall quality of the included literature.

Using the GRADE framework, the certainty of evidence for the two primary diagnostic tasks was rated as moderate (Table 3 and Table S6 in Multimedia Appendix 1). Following the GRADE Summary of Findings format for diagnostic test accuracy, Table 3 reports pooled sensitivity and specificity, expected results per 1000 tested at a 20% pretest probability, PIs, certainty ratings, and plain-language interpretation. The detailed data extraction tables and PROBAST+AI risk-of-bias tables are retained in the Supplementary Materials because of their length (Tables S4-S5 and S7-S10 in Multimedia Appendix 1).

In Table 3, expected results were calculated per 1000 tested at an illustrative pretest probability of 20% (200 with the target condition and 800 without); this assumed prevalence may vary across clinical settings. Diagnostic accuracy was treated as a surrogate for patient-important consequences because direct evidence on whether artificial intelligence–assisted testing improves visual outcomes, referral burden, or treatment timing was unavailable. For age-related macular degeneration versus normal, the target condition was age-related macular degeneration; for wet age-related macular degeneration versus dry age-related macular degeneration, the target condition was wet age-related macular degeneration.

**Table 3.** GRADE^a^ summary of findings table for deep learning–based AMD^b^ image classification.

x test	No. of studies and validation datasets	Sensitivity (95% CI)	Specificity (95% CI)	Expected results per 1000 tested at 20% prevalence	95% prediction interval	Certainty	Plain-language interpretation
DL^c^ for AMD versus normal	27 studies [19-36,38-46] and 37 validation datasets	0.98 (0.96‐0.99)	0.98 (0.95‐0.99)	TP^d^ 196 FN^e^ 4 TN^f^ 784 FP^g^ 16	Sensitivity 0.95‐0.99 Specificity 0.95‐0.99	Moderate^h^	At a 20% pretest probability, DL would correctly identify most AMD cases and correctly rule out most non-AMD eyes or images. The prediction intervals indicate that performance may vary across settings.
DL for wAMD^i^ versus dAMD^j^	16 studies [19,23-29,31,33,37,41-44,46] and 22 validation datasets	0.95 (0.91‐0.97)	0.95 (0.93‐0.97)	TP 190 FN 10 TN 760 FP 40	Sensitivity 0.89‐0.97 Specificity 0.92‐0.97	Moderate^h^	At a 20% pretest probability, DL would correctly identify most wAMD cases and correctly classify most dAMD cases. The wider prediction interval for sensitivity supports cautious local validation before deployment.

a GRADE: Grading of Recommendations, Assessment, Development, and Evaluation.

b AMD: age-related macular degeneration.

c DL: deep learning.

d TP: true positive.

e FN: false negative.

f TN: true negative.

g FP: false positive.

h Downgraded one GRADE level for risk of bias because most included studies were retrospective, many relied on internal validation or incompletely reported patient-level separation or reference standards, and PROBAST+AI identified high risk of bias in a subset of validation or testing studies; detailed domain-level judgments are provided in Table S6 in Multimedia Appendix 1.

i wAMD: wet age-related macular degeneration.

j dAMD: dry age-related macular degeneration.

### Goodness-of-Fit and Model Diagnostics

The goodness-of-fit and bivariate normality of the bivariate mixed-effects regression models were evaluated through graphical inspection (Figures S1 and S2 in Multimedia Appendix 1). The goodness-of-fit plots (Figures S1A and S2A in Multimedia Appendix 1), illustrating the normal probability of deviance residuals, demonstrated that the observed data points adhered closely to the reference diagonal line, indicating a robust model fit. Similarly, the bivariate normality plots (Figures S1B and S2B in Multimedia Appendix 1) revealed a linear alignment of data points within the chi-square probability plots, confirming that the random effects of sensitivity and specificity followed a bivariate normal distribution. Collectively, these diagnostic assessments substantiate the validity and statistical robustness of the models used in this systematic review and meta-analysis.

### DL Algorithms Versus Ophthalmologists for AMD Versus Normal Classification

**Figure 3.**
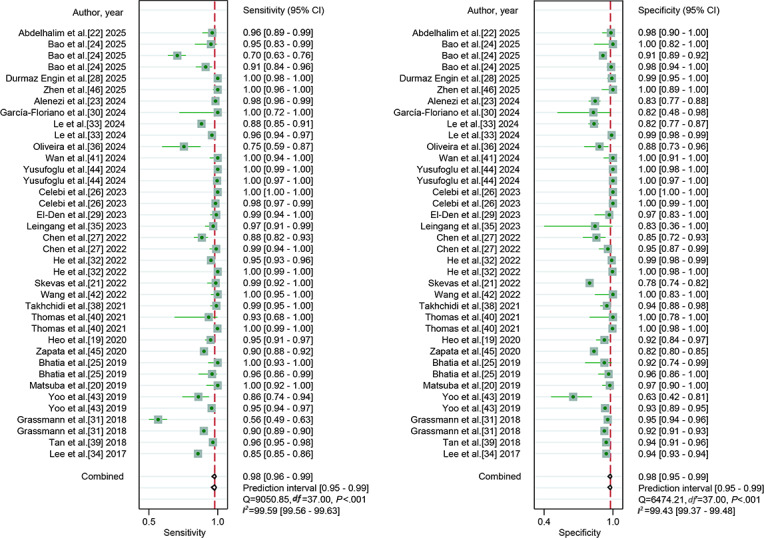
Primary forest plots for pooled sensitivity, specificity, and accuracy of deep learning algorithms for classifying age-related macular degeneration from normal retinas. Prediction intervals are printed in the plots to show expected between-setting variability [19-36,38-46].

The pooled diagnostic performance of image-based DL algorithms for distinguishing AMD from normal retinas was high across threshold-dependent metrics and threshold-independent discrimination. Sensitivity and specificity quantify performance at the diagnostic thresholds reported by individual studies, accuracy reflects overall correct classification in the analyzed datasets, and AUC describes discrimination across possible thresholds. The primary forest plots with PIs are shown in Figure 3, and the intervals should be interpreted as indicating expected between-setting variability rather than only statistical uncertainty around the pooled estimate.

**Figure 4.**
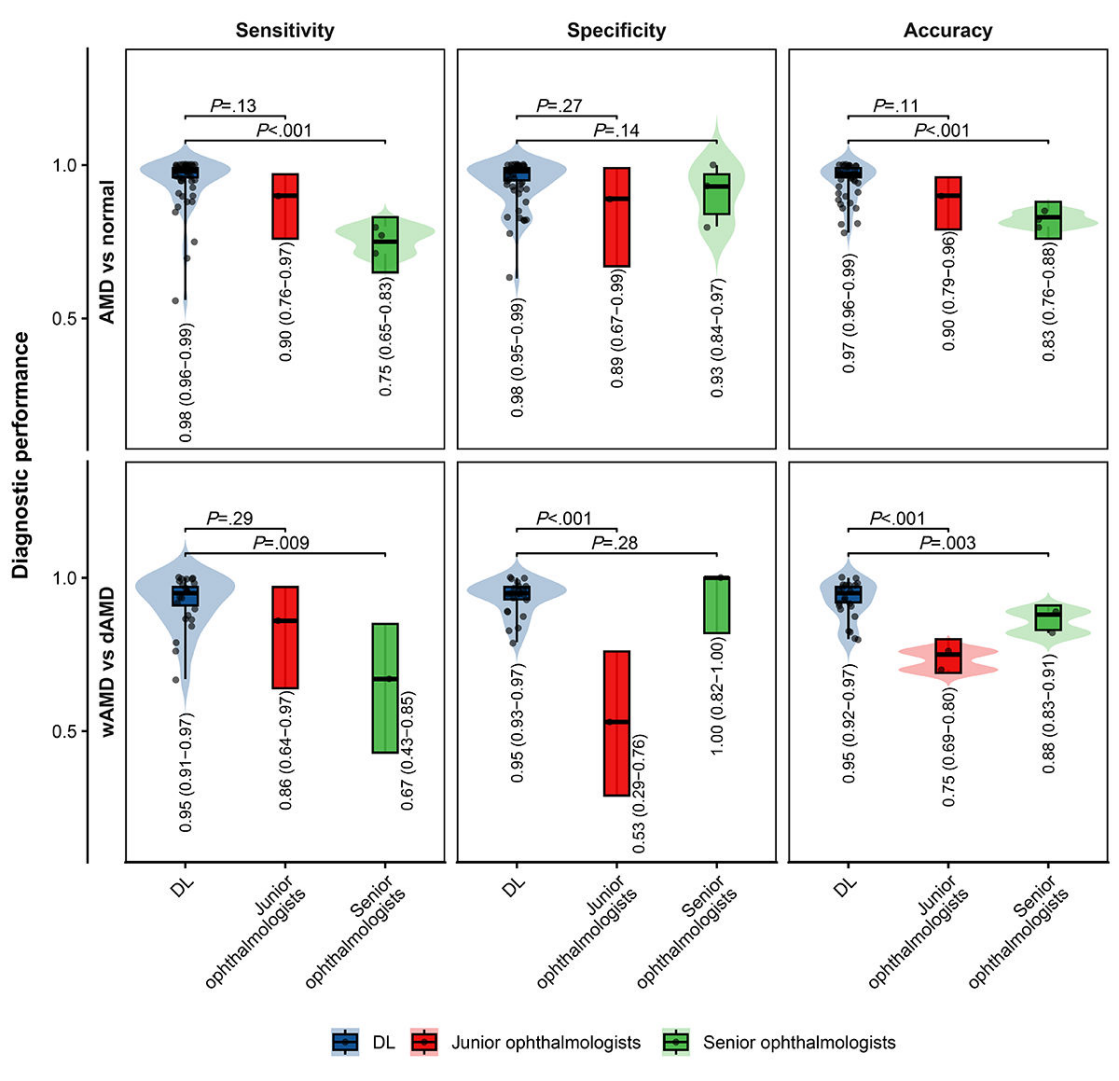
Violin plots comparing the diagnostic performance of deep learning algorithms versus junior and senior ophthalmologists. The top row displays the sensitivity, specificity, and accuracy for classifying age-related macular degeneration versus normal, while the bottom row displays the performance for classifying wet age-related macular degeneration versus dry age-related macular degeneration. The scattered dots represent individual study estimates, and the internal box plots indicate the median and interquartile range. *P* values indicate the statistical significance of the comparisons. AMD: age-related macular degeneration; dAMD: dry age-related macular degeneration; DL: deep learning; wAMD: wet age-related macular degeneration.

Comparative analysis revealed that the pooled sensitivity of DL algorithms was significantly higher than that of senior ophthalmologists (0.98 vs 0.75; Z=4.94; *P*<.001), as was the pooled accuracy (0.97 vs 0.83; Z=4.43; *P*<.001). These results were detailed in Figure 4 and Figures S5 and S6 in Multimedia Appendix 1. Notably, the comparison with junior ophthalmologists for AMD detection was limited to a single study [24], precluding robust statistical inference for this subgroup.

### DL Algorithms Versus Ophthalmologists for wAMD Versus dAMD Classification

**Figure 5.**
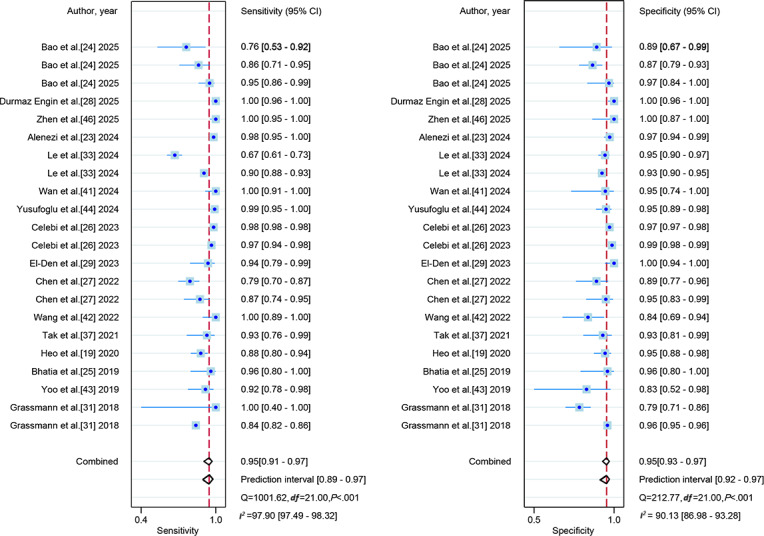
Primary forest plots for pooled sensitivity, specificity, and accuracy of deep learning algorithms for classifying wet age-related macular degeneration from dry age-related macular degeneration. Prediction intervals are printed in the plots to show expected between-setting variability [19,23-29,31,33,37,41-44,46].

For the classification of wAMD versus dAMD, DL algorithms again showed high pooled sensitivity, specificity, accuracy, and AUC, but these metrics describe different behaviors. Accuracy summarizes correct classification within the included datasets, whereas AUC reflects discrimination across thresholds and may remain high even when real-world threshold selection, disease spectrum, or image quality differs. The primary forest plots with PIs are shown in Figure 5.

Comparative analyses showed that, within the limited head-to-head datasets, DL algorithms had higher pooled specificity (0.95 vs 0.53; Z=3.49; *P*<.001) and diagnostic accuracy (0.95 vs 0.75; Z=6.48; *P*<.001) than junior ophthalmologists. Compared with senior ophthalmologists, DL algorithms had higher pooled sensitivity (0.95 vs 0.67; Z=2.58; *P*=.009) and diagnostic accuracy (0.95 vs 0.88; Z=2.90; *P*=.003). All comparisons are shown in Figure 4 and Figures S5 and S6 in Multimedia Appendix 1. These findings suggest a possible relative performance advantage for DL in selected metrics, but the small number of clinician-comparison studies means that the results should be interpreted as preliminary rather than definitive.

### Subgroup Analysis for AMD Versus Normal Classification

**Figure 6.**
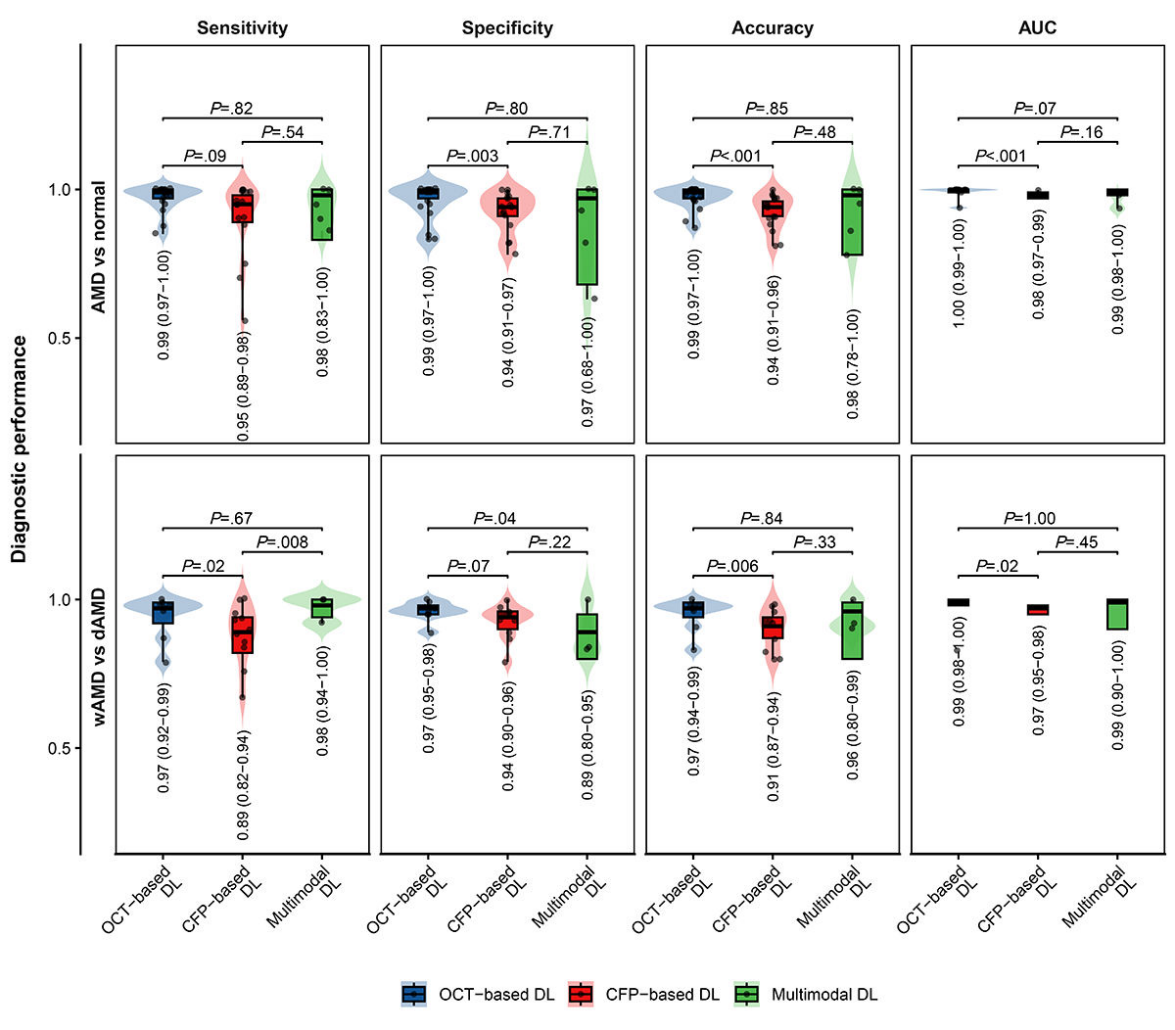
Subgroup analysis of deep learning algorithms based on imaging modalities. The violin plots illustrate the distribution of sensitivity, specificity, accuracy, and area under the curve for optical coherence tomography-based, color fundus photography-based, and multimodal models in classifying age-related macular degeneration versus normal (top row) and wet age-related macular degeneration versus dry age-related macular degeneration (bottom row). The scattered dots represent individual study estimates, and the internal box plots indicate the median and interquartile range. *P* values indicate the statistical significance of the comparisons. AMD: age-related macular degeneration; AUC: area under the curve; CFP: color fundus photography; dAMD: dry age-related macular degeneration; DL: deep learning; OCT: optical coherence tomography; wAMD: wet age-related macular degeneration.

Subgroup analysis based on imaging modality, as illustrated in Figure 6 and Figures S9-S17 in Multimedia Appendix 1, revealed statistically significant performance differences among DL algorithms. Specifically, OCT-based DL algorithms demonstrated significantly higher pooled specificity compared to CFP-based algorithms (0.99 vs 0.94; Z=2.92; *P*=.003). Furthermore, OCT-based algorithms achieved significantly higher pooled accuracy (0.99 vs 0.94; Z=3.36; *P*<.001) and a significantly higher pooled AUC value (1.00 vs 0.98; Z=3.50, *P*<.001) than CFP-based DL algorithms.

### Subgroup Analysis for wAMD Versus dAMD Classification

The subgroup analysis by imaging modality for wAMD versus dAMD classification was presented in Figure 6 and Figures S18-S27 in Multimedia Appendix 1. OCT-based DL algorithms demonstrated significantly higher pooled sensitivity than CFP-based algorithms (0.97 vs 0.89; Z=2.25; *P*=.02). Their pooled accuracy was also significantly higher (0.97 vs 0.91; Z=2.73; *P*=.006), as was their pooled AUC value (0.99 vs 0.97; Z=2.17; *P*=.02).

### Heterogeneity Investigation: Bivariate Boxplot and Meta-Regression

**Figure 7.**
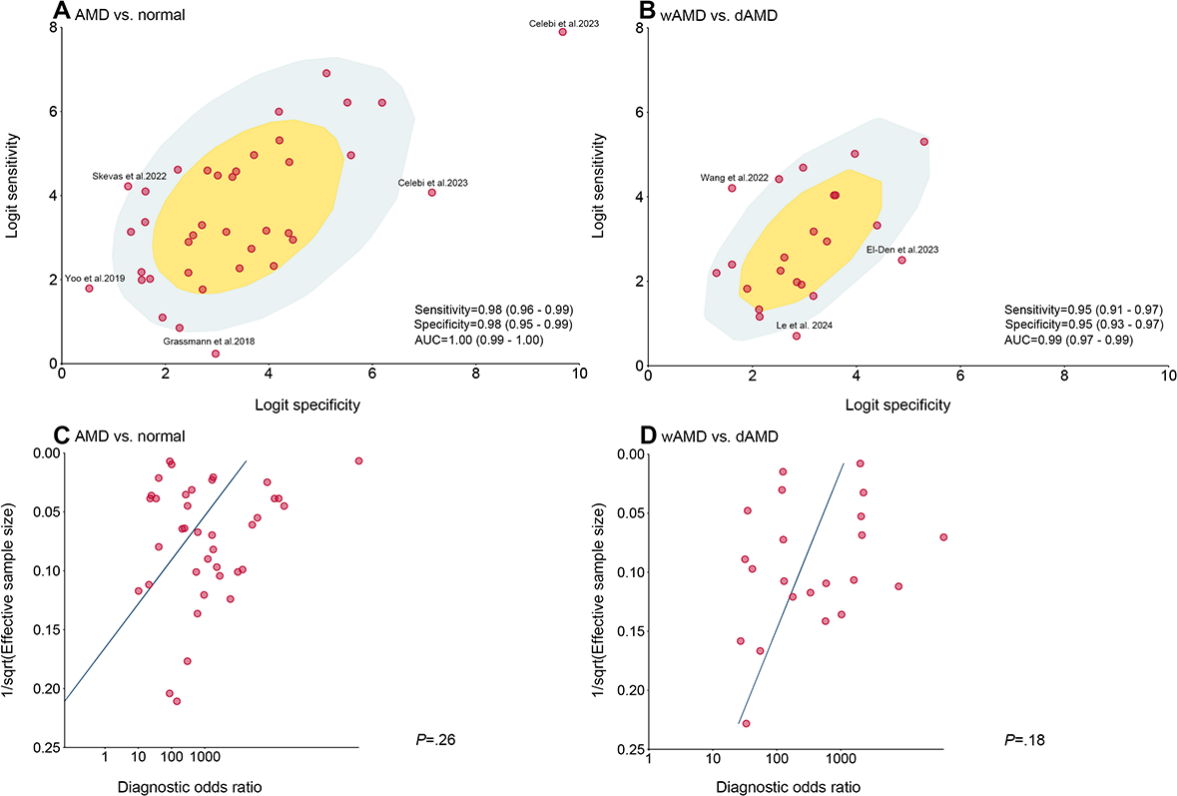
Assessment of heterogeneity and small-study effects. (A and B) Bivariate boxplots identifying potential outliers and influential studies for age-related macular degeneration versus normal and wet age-related macular degeneration versus dry age-related macular degeneration classifications. Studies falling outside the colored ellipses are considered outliers. (C and D) Deeks’ funnel plots evaluating small-study effects for the two classification tasks; a *P* value >.10 indicates no significant small-study effects. AMD: age-related macular degeneration; dAMD: dry age-related macular degeneration; wAMD: wet age-related macular degeneration [21,26,29,31,33,42,43].

To explore the substantial statistical heterogeneity observed, meta-regression and bivariate boxplot analyses were conducted. For the AMD versus normal classification, meta-regression indicated that the type of validation (internal validation vs external validation), database source (open database vs private database), and study centers (single center vs multicenter) were potential sources of heterogeneity (Table S12 in Multimedia Appendix 1). The bivariate boxplot suggested that the studies by Skevas et al [21], Celebi et al [26], Grassmann et al [31], and Yoo et al [43], might be influential outliers contributing to the heterogeneity (Figure 7A).

For the wAMD versus dAMD classification, meta-regression identified the type of validation (internal validation vs external validation), type of imaging (unimodal vs multimodal), database source (open database vs private database), and study centers (single center vs multicenter) design as potential moderators explaining heterogeneity (Table S13 in Multimedia Appendix 1). The corresponding bivariate boxplot highlighted the studies by El-Den et al [29], Le et al [33], and Wang et al [42] as potential outliers influencing the pooled estimates (Figure 7B).

### Sensitivity Analysis

Sensitivity analyses were conducted to assess the robustness of our findings. Whether excluding studies flagged as high-risk in the validation set by the PROBAST+AI tool, or removing the identified outliers and influential data points, the variations in diagnostic performance were stable (Tables S14 and S15 in Multimedia Appendix 1). The results remained consistent with the primary analysis, demonstrating that the conclusions are robust and not disproportionately driven by these extreme studies.

### Small-Study Effects and Clinical Applicability

**Figure 8.**
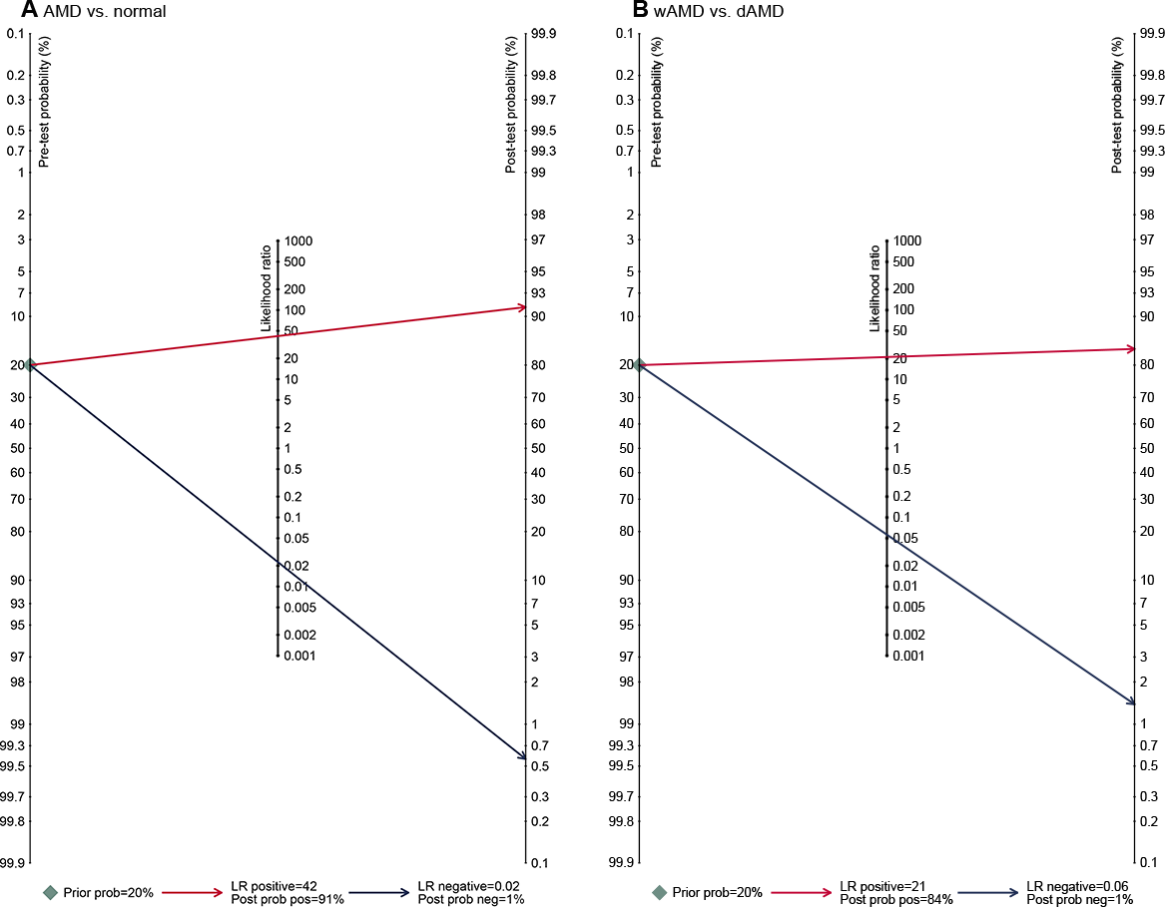
Fagan’s nomograms evaluating the clinical utility of deep learning algorithms. (A) Clinical utility for classifying age-related macular degeneration versus normal. (B) Clinical utility for classifying wet age-related macular degeneration versus dry age-related macular degeneration. The left axis represents the pretest probability (set at 20%), the middle axis represents the likelihood ratio, and the right axis represents the posttest probability. AMD: age-related macular degeneration; dAMD: dry age-related macular degeneration; wAMD: wet age-related macular degeneration.

Deeks’ funnel plot asymmetry test indicated no evidence of significant small-study effects for either AMD classification task (*P*=.26 and 0.18; Figures 7C and 7D). Assuming a pretest probability of 20%, Fagan nomogram analysis demonstrated that a positive DL test result increased the posttest probability to 91% for AMD versus normal and 84% for wAMD versus dAMD, while a negative result reduced it to 1% for both tasks (Figures 8A and 8B).

## Discussion

In relation to our objective of comparing DL algorithms with ophthalmologists and identifying factors that influence diagnostic performance, the main finding is that DL models showed strong pooled performance for both AMD detection and wAMD versus dAMD classification, but the strength of this evidence differs across comparisons. The clinician comparisons suggest a possible role for DL as a consistent decision-support baseline, yet the sparse junior-ophthalmologist data and wide between-study variability mean that these findings should be interpreted as hypothesis-generating rather than definitive for deployment.

Our meta-analysis reveals a deployment-relevant pattern. Within the analyzed datasets, DL algorithms demonstrated significantly higher pooled sensitivity and accuracy compared to the available metrics for senior ophthalmologists in distinguishing AMD from normal controls; however, no significant differences were observed between DL and junior ophthalmologists across any diagnostic metrics. The higher pooled performance of DL suggests that these models possess an enhanced capability to detect subtle, pixel-level morphological changes and nonlinear feature interactions, such as early exudative signs, that may elude the visual inspection of even the most experienced clinicians [20,24,36]. Conversely, the lower sensitivity observed in senior ophthalmologists appears to be behaviorally driven by specific decision thresholds rather than skill deficiencies. As evidenced by the data from studies such as Matsuba et al [20], Bao et al [24], and Oliveira et al [36], senior experts exhibited a distinct preference for conservative diagnostic thresholds, achieving high specificity (summary 0.93) but notably reduced sensitivity (summary 0.75). This indicates a clinical preference for “rule-in” strategies to strictly avoid false positives, a constraint that DL algorithms do not possess. However, the unexpected parity between DL algorithms and junior practitioners should be interpreted with caution; this finding is attributable to data sparsity rather than clinical equivalence, as the analysis is restricted to only one study for AMD detection and two studies for wAMD classification, introducing a high risk of small-sample bias [20,24,36]. Similarly, the comparisons with senior ophthalmologists, while more robust, remain limited in number (three studies for AMD detection, two for wAMD classification), and the resulting estimates should be considered preliminary rather than definitive. This data-driven limitation, however, stands in contrast to the wAMD versus dAMD task, where sufficient head-to-head comparisons (two studies) enabled a stratified analysis, revealing distinct behavioral patterns across experience levels [20,24,36].

The very high AUC values observed in our analysis warrant careful interpretation and should not be equated with flawless real-world diagnostic performance. Restricted test distributions, curated image quality, internal validation, repeated use of public datasets, model selection based on the best-performing algorithm, and insufficiently documented patient-level splitting may inflate discrimination [25,38,43,46]. Potential data leakage cannot be excluded in studies that did not clearly separate patients, eyes, or images across training, validation, and test sets. Therefore, AUCs close to 1.0 indicate excellent discrimination within the analyzed datasets, not proof of flawless performance in prospective clinical workflows [47,48].

In the clinically critical task of differentiating wAMD from dAMD, automated DL systems demonstrated robustness across varying datasets. Our results indicated that the pooled metrics of DL algorithms not only showed higher specificity and accuracy compared to junior ophthalmologists but also indicated higher sensitivity and accuracy relative to senior ophthalmologists. Rather than framing this as DL mitigating an “experience gap” or correcting specific human errors, these findings suggest that DL algorithms offer a more consistent and objective diagnostic baseline that balances sensitivity and specificity. These findings advocate for a collaborative clinical paradigm: DL algorithms could serve as a triage filter to enhance specificity for primary care providers while functioning as a high-sensitivity “second reader” for specialists resolving equivocal wAMD cases [49].

Interestingly, our subgroup analysis highlights the relatively consistent performance of OCT-based models for automated AMD classification. OCT-based models significantly outperformed CFP-based approaches, driven by the capture of pathognomonic cross-sectional features—such as intraretinal fluid and pigment epithelial detachment—that are often obscured in 2D fundus photography [50]. Intriguingly, multimodal DL (OCT + CFP) did not significantly surpass standalone OCT models. This suggests a “saturation effect,” where the rich structural data of OCT capture the vast majority of diagnostic signals, rendering the incremental value of CFP marginal [35]. In practice, the underperformance of multimodal models relative to standalone OCT may also stem from feature redundancy and fusion noise [51]; when OCT and CFP capture substantially overlapping diagnostic information [43], their combination can paradoxically introduce variance through misaligned spatial features, registration errors, and conflicting feature representations, ultimately degrading rather than enhancing the decision boundary. From a translational perspective, this finding is pivotal; it implies that the computational cost and technical challenges of multimodal alignment (eg, fusion noise and registration errors) may currently outweigh the clinical benefits [51,52]. Therefore, unless fusion strategies are substantially optimized, OCT-based workflows currently appear to be a practical foundation for clinical deployment [35].

Building upon the baselines established by previous meta-analyses, this systematic review advances the understanding of DL in AMD diagnosis. In 2023, Leng et al [9] reported a pooled sensitivity of 94% and specificity of 97% for convolutional neural network algorithms. More recently, Chen et al [27] highlighted the superiority of AI over retinal specialists. Our analysis incorporates the latest studies using advanced architectures, such as Vision Transformers. This inclusion yields modestly higher pooled metrics, reflecting the field’s technological maturation [33]. Most significantly, this systematic review distinguishes itself through four methodological innovations that enhance clinical relevance: (1) a stratified comparison of AI versus ophthalmologists, explicitly differentiating by experience level; (2) the application of the PROBAST+AI tool for bias assessment, complemented by the GRADE framework; (3) a rigorous subgroup analysis by imaging modality (OCT, CFP, multimodal) to isolate technical performance drivers; and (4) a granular evaluation extending beyond binary detection to the specific classification of wAMD versus dAMD. Collectively, these advancements establish a more robust evidence base than prior reviews.

Heterogeneity is central to the interpretation of these findings [53]. Extreme between-study variability should not be treated only as a statistical descriptor; it indicates that pooled estimates may not transfer reliably to clinics with different devices, acquisition protocols, labeling rules, disease spectra, or patient populations [53-55]. By using a bivariate random-effects model and multivariable meta-regression, we identified that differences in validation strategies (internal vs external), database sources (open vs. private), and study settings (single-center vs. multicenter) significantly influence diagnostic performance. Studies relying solely on internal validation frequently reported inflated metrics, illustrating a generalization gap when models face domain shifts in image acquisition or demographics [50]. Similarly, single-center studies risk overfitting to specific center features arising from uniform protocols, whereas multicenter designs typically demonstrate greater robustness through exposure to diverse image qualities [50]. Consequently, our analysis suggests that database diversity and annotation quality are likely more critical determinants of generalizability than mere data accessibility. Furthermore, specific outliers in the bivariate box plot (Skevas et al [21], Celebi et al [26], Grassmann et al [31], El-Den et al [29], Le et al [33], Wang et al [42], and Yoo et al [43]) highlight how methodological divergences, such as algorithm architecture and data curation strategies, can materially affect performance. This indicates that future improvements in AI reliability will depend less on novel model architectures and more on the curation of diverse, multicenter external validation datasets. The substantial heterogeneity observed in this systematic review and meta-analysis warrants careful interpretation. Rather than reflecting routine statistical noise, this level of heterogeneity signals the pooling of fundamentally diverse data sources. Specifically, the included studies used different imaging hardware (eg, Heidelberg Spectralis, Topcon, Zeiss Cirrus OCT devices; various fundus camera systems), acquisition protocols (varying image resolutions, fields of view, and scan patterns), and ground-truth labeling methodologies (ranging from consensus grading by multiple retinal specialists to single-expert annotation or semi-automated classification systems) [32,35,45]. These technical and methodological differences fundamentally influence the feature space available to DL algorithms and likely account for much of the observed heterogeneity. The PIs (eg, sensitivity: 0.95‐0.99; specificity: 0.95‐0.99 for distinguishing AMD from normal retinas; sensitivity: 0.89‐0.97; specificity: 0.92‐0.97 for classifying wAMD vs dAMD) further underscore that the average pooled performance, while encouraging, may not be representative of performance in any individual deployment setting. This finding has important implications for clinical deployment: site-specific validation using local imaging equipment and patient populations remains essential before implementing any DL-based AMD screening system.

These heterogeneity findings provide concrete guidance for future study design [56]. Investigators should prioritize external validation on datasets from institutions and populations distinct from the training data [53], use multicenter designs incorporating diverse imaging devices and acquisition protocols, report patient-level separation between training and testing data, and stratify performance by imaging device, acquisition protocol, labeling method, and patient demographics [56]. Because PIs indicate that local performance may differ from pooled estimates, summary metrics alone should not be used as a deployment decision rule [54].

Translating these findings into practice, DL algorithms exhibit the potential to augment the diagnostic workflow rather than replace it. The superior accuracy of DL in classifying wAMD versus dAMD suggests potential use in resource-limited settings or tele-ophthalmology screening. However, considering current algorithms are primarily trained on isolated OCT or CFP images, they often lack integration with other imaging modalities or clinical parameters; future models should therefore evaluate multimodal imaging and patient clinical contexts to emulate comprehensive diagnoses [57]. Beyond these technical and clinical considerations, significant implementation barriers persist, including the scarcity of expert-annotated data, regulatory hurdles, and technical challenges regarding data availability, model interpretability, transparency, and generalization capability [36,52,58]. Advances in few-shot learning, self-supervised models, and centralized platforms may support a more integrated AI ecosystem, requiring sustained multidisciplinary efforts to optimize AI safety and support safe clinical practice [52,58].

Beyond diagnostic performance metrics, the successful clinical translation of DL algorithms requires addressing practical implementation challenges. These include seamless integration into existing electronic health record systems and ophthalmic imaging workflows, real-time processing capabilities compatible with clinical time constraints, and intuitive user interfaces that present AI-generated results in a manner that supports rather than disrupts clinical decision-making [46]. Furthermore, clinician trust and acceptance—shaped by model interpretability, transparency of AI reasoning, and consistent performance across diverse clinical scenarios—are prerequisites for successful adoption [23,24]. Future validation of DL tools must therefore extend beyond accuracy benchmarks to encompass usability studies, clinician acceptance evaluations, and workflow efficiency assessments in real-world clinical settings [41].

Our findings should be interpreted considering several limitations. First, the predominance of retrospective study designs (26 of 28 included studies) represents a fundamental limitation that must be carefully considered when interpreting the strong pooled performance metrics. Retrospective datasets are typically curated from clinical archives, which may systematically exclude poor-quality images, atypical presentations, and diagnostically challenging cases that are routinely encountered in prospective clinical workflows. This selection inherently inflates the apparent diagnostic performance and limits the generalizability of our findings to real-world screening and clinical deployment settings [48]. Second, to address potential patient overlap and maintain statistical independence, we extracted performance metrics exclusively from the primary AI algorithm within each study, omitting data from suboptimal models. While methodologically sound for meta-analysis, this approach inherently reflects a “best-case scenario” that likely inflates the pooled performance estimates compared to average algorithmic performance. This reporting bias is an inherent limitation of the current DL literature in ophthalmology and should be carefully considered by clinicians and policymakers when interpreting these results for clinical implementation decisions [12]. Future research should therefore granularly evaluate performance variances across different algorithmic architectures, including less optimal models, to ensure a more balanced and realistic assessment of the DL landscape. Third, direct head-to-head comparisons between DL algorithms and ophthalmologists were small, particularly for the AMD versus normal task where only one study provided junior ophthalmologist data, limiting the statistical power of these specific subgroups [50]. Future research must prioritize prospective, multicenter trials with prespecified human comparison arms to definitively validate these retrospective results [12].

In conclusion, this systematic review suggests that, compared with ophthalmologists, DL algorithms demonstrate superior and more balanced diagnostic performance for AMD image classification, providing a consistent decision-support baseline that mitigates the threshold-dependent trade-offs observed in human graders. However, these relative-performance findings remain preliminary because head-to-head evidence is sparse, especially for junior ophthalmologists, and because wide PIs, high heterogeneity, retrospective designs, and possible inflation from restricted datasets, internal validation, or leakage limit clinical transportability. DL systems should therefore be locally calibrated and prospectively validated as triage adjuncts rather than autonomous replacements. Before implementation, prospective multicenter studies should test representative patients, use strict patient-level external validation [56], include prespecified human comparison arms [59], and evaluate workflow integration, interpretability, and safety [60].

## Supplementary material

10.2196/97174 Multimedia Appendix 1 Detailed methodology, search strategies, quality assessments, subgroup analyses, and sensitivity analyses.

10.2196/97174 Checklist 1 PRISMA checklist.

## Abbreviations

AI: artificial intelligence
AMD: age-related macular degeneration
AUC: area under the curve
CFP: color fundus photography
dAMD: dry age-related macular degeneration
DL: deep learning
FN: false negative
FP: false positive
GRADE: Grading of Recommendations, Assessment, Development, and Evaluations
OCT: optical coherence tomography
PI: prediction interval
PITROS: Patient, Index test, Target condition, Reference standard, Outcome, and Setting
PRISMA: Preferred Reporting Items for Systematic Reviews and Meta-Analyses
PRISMA-DTA: Preferred Reporting Items for Systematic Reviews and Meta-Analyses of Diagnostic Test Accuracy
PROBAST+AI: Prediction model Risk Of Bias Assessment Tool for Artificial Intelligence
PROSPERO: International Prospective Register of Systematic Reviews
TN: true negative
TP: true positive
wAMD: wet age-related macular degeneration
