# Supplementary material for: Performance of Deep Learning in Classifying Age-Related Macular Degeneration From Images: Systematic Review and Meta-Analysis
Source: J Med Internet Res. 2026 Jun 15;28:e97174. doi: 10.2196/97174 (PMC13268637; doi:10.2196/97174)
Supplement: Multimedia Appendix 1 [file jmir-v28-e97174-s001.pdf]

Multimedia Appendix 1

Performance of Deep Learning in Classifying Age-related Macular Degeneration from Images: Systematic Review and Meta-analysis

---

This supplementary document supports the main manuscript and provides detailed methodological descriptions, additional results, comprehensive search strategies, and quality assessment data.

Table of Contents

Table S1. Preferred Reporting Items for Systematic Reviews and Meta-Analyses of Diagnostic Test Accuracy (PRISMA-DTA) Checklist....6

Table S2. PRISMA 2020 for Abstracts Checklist. 8

Table S3. Search strategy in PubMed, Embase, Web of Science, and Cochrane Library. 9

Table S4. Risk of bias assessment (PROBAST+AI) model development based on seven domains. 11

Table S5. Risk of bias assessment (PROBAST+AI) model evaluation based on seven domains. 13

Table S6. Detailed GRADE assessment criteria and certainty judgments for all pooled outcomes. 16

Table S7. Technical aspects of age-related macular degeneration (AMD) versus normal in the included studies....17

Table S8. Diagnostic performance of age-related macular degeneration (AMD) versus normal in the included studies....19

Table S9. Technical aspects of wet age-related macular degeneration (wAMD) versus dry age-related macular degeneration (dAMD) in the included studies....21

Table S10. Diagnostic performance of wet age-related macular degeneration (wAMD) versus dry age-related macular degeneration (dAMD) in the included studies. 22

Table S11. Diagnostic data for different level of ophthalmologists. 23

Table S12. Subgroup analysis and meta-regression analysis for age-related macular degeneration (AMD) vs. normal....24

Table S13. Subgroup analysis and meta-regression analysis for wet age-related macular degeneration (wAMD) vs. dry age-related macular degeneration (dAMD). 25

Table S14. Sensitivity analysis of deep learning algorithms for classifying age-related macular degeneration (AMD) from normal under different exclusion criteria....26

Table S15. Sensitivity analysis of deep learning algorithms for classifying wet age-related macular degeneration (wAMD) from dry age-related macular degeneration (dAMD) under different exclusion criteria....27

Figure S1. Graphical assessment of model fit, bivariate normality, and influence diagnostics for deep learning algorithms classifying age-related macular degeneration (AMD) from normal....28

Figure S2. Graphical assessment of model fit, bivariate normality, and influence diagnostics for deep learning algorithms classifying wet age-related macular degeneration (wAMD) from dry age-related macular degeneration (dAMD). 29

Figure S3. Forest plots of the pooled accuracy of deep learning algorithms using generalized linear mixed model (GLMM) for classifying age-related macular degeneration

(AMD) from normal. 30

Figure S4. Summary receiver operating characteristic (SROC) curve of deep learning algorithms for classifying age-related macular degeneration (AMD) from normal.....31

Figure S5. Forest plots of the pooled sensitivity and specificity of deep learning algorithms of junior and senior ophthalmologists for classifying age-related macular degeneration (AMD) from normal and wet age-related macular degeneration (wAMD) from dry age-related macular degeneration (dAMD). 32

Figure S6. Forest plots of the pooled accuracy of deep learning algorithms using generalized linear mixed model (GLMM) of junior and senior ophthalmologists for classifying age-related macular degeneration (AMD) from normal and wet age-related macular degeneration (wAMD) from dry age-related macular degeneration (dAMD). 33

Figure S7. Forest plots of the pooled accuracy of deep learning algorithms using generalized linear mixed model (GLMM) for classifying wet age-related macular degeneration (wAMD) from dry age-related macular degeneration (dAMD). 34

Figure S8. Summary receiver operating characteristic (SROC) curve of deep learning algorithms for classifying wet age-related macular degeneration (wAMD) from dry age-related macular degeneration (dAMD). 35

Figure S9. Forest plots of the pooled sensitivity and specificity of deep learning algorithms for classifying age-related macular degeneration (AMD) from normal based on optical coherence tomography (OCT) images. 36

Figure S10. Forest plots of the pooled accuracy of deep learning algorithms using generalized linear mixed model (GLMM) for classifying age-related macular degeneration (AMD) from normal based on optical coherence tomography (OCT) images. 37

Figure S11. Summary receiver operating characteristic (SROC) curve of deep learning algorithms for classifying age-related macular degeneration (AMD) from normal based on optical coherence tomography (OCT) images. 38

Figure S12. Forest plots of the pooled sensitivity and specificity of deep learning algorithms for classifying age-related macular degeneration (AMD) from normal based on color fundus photography (CFP) images. 39

Figure S13. Forest plots of the pooled accuracy of deep learning algorithms using generalized linear mixed model (GLMM) for classifying age-related macular degeneration (AMD) from normal based on color fundus photography (CFP) images. 40

Figure S14. Summary receiver operating characteristic (SROC) curve of deep learning algorithms for classifying age-related macular degeneration (AMD) from normal based on color fundus photography (CFP) images. 41

Figure S15. Forest plots of the pooled sensitivity and specificity of deep learning algorithms for classifying age-related macular degeneration (AMD) from normal based on multimodal images. 42

Figure S16. Forest plots of the pooled accuracy of deep learning algorithms using generalized linear mixed model (GLMM) for classifying age-related macular degeneration (AMD) from normal based on multimodal images. 43

Figure S17. Summary receiver operating characteristic (SROC) curve of deep learning algorithms for classifying age-related macular degeneration (AMD) from normal based on multimodal images. 44

Figure S18. Forest plots of the pooled sensitivity and specificity of deep learning algorithms for classifying wet age-related macular degeneration (wAMD) from dry age-related macular degeneration (dAMD) based on optical coherence tomography (OCT) images. 45

Figure S19. Forest plots of the pooled accuracy of deep learning algorithms using generalized linear mixed model (GLMM) for classifying wet age-related macular degeneration (wAMD) from dry age-related macular degeneration (dAMD) based on optical coherence tomography (OCT) images. 46

Figure S20. Summary receiver operating characteristic (SROC) curve of deep learning algorithms for classifying wet age-related macular degeneration (wAMD) from dry age-related macular degeneration (dAMD) based on optical coherence tomography (OCT) images. 47

Figure S21. Forest plots of the pooled sensitivity and specificity of deep learning algorithms for classifying wet age-related macular degeneration (wAMD) from dry age-related macular degeneration (dAMD) based on color fundus photography (CFP) images. 48

Figure S22. Forest plots of the pooled accuracy of deep learning algorithms using generalized linear mixed model (GLMM) for classifying wet age-related macular degeneration (wAMD) from dry age-related macular degeneration (dAMD) based on color fundus photography (CFP) images. 49

Figure S23. Summary receiver operating characteristic (SROC) curve of deep learning algorithms for classifying wet age-related macular degeneration (wAMD) from dry age-related macular degeneration (dAMD) based on color fundus photography (CFP) images. 50

Figure S24. Forest plots of the pooled sensitivity of deep learning algorithms for classifying wet age-related macular degeneration (wAMD) from dry age-related macular degeneration (dAMD) based on multimodal images. 51

Figure S25. Forest plots of the pooled specificity of deep learning algorithms for classifying wet age-related macular degeneration (wAMD) from dry age-related macular degeneration (dAMD) based on multimodal images. 52

Figure S26. Forest plots of the pooled accuracy of deep learning algorithms using generalized linear mixed model (GLMM) for classifying wet age-related macular degeneration (wAMD) from dry age-related macular degeneration (dAMD) based on multimodal images. 53

Figure S27. Summary receiver operating characteristic (SROC) curve of deep learning algorithms for classifying wet age-related macular degeneration (wAMD) from dry age-related macular degeneration (dAMD) based on multimodal images. 54

References 55

Table S1. Preferred Reporting Items for Systematic Reviews and Meta-Analyses of Diagnostic Test Accuracy (PRISMA-DTA) Checklist.

**TITLE/ABSTRACT**			Reported on page #
Title	1	Identify the report as a systematic review (+/−meta-analysis) of diagnostic test accuracy (DTA) studies	1
Abstract	2	See the PRISMA for Abstracts checklist.	4-5
**INTRODUCTION**			
Rationale	3	Describe the rationale for the review in the context of what is already known	6-8
Clinical role of index test	D1	State the scientific and clinical background, including the intended use and clinical role of the index test, and if applicable, the rationale for minimally acceptable test accuracy (or minimum difference in accuracy for comparative design)	6-8
Objectives	4	Provide an explicit statement of question(s) being addressed in terms of participants, index test(s), and target condition(s)	8
**METHODS**			
Protocol and registration	5	Indicate if a review protocol exists, if and where it can be accessed (e.g., Web address), and, if available, provide registration information including registration number.	8
Eligibility criteria	6	Specify study characteristics (participants, setting, index test(s), reference standard(s), target condition(s), and study design) and report characteristics (e.g., years considered, language, publication status) used as criteria for eligibility, giving rationale.	9, 39
Information sources	7	Describe all information sources (e.g., databases with dates of coverage, contact with study authors to identify additional studies) in the search and date last searched.	8-9
Search	8	Present full search strategies for all electronic databases and other sources searched, including any limits used, such that they could be repeated.	8-9
Selection process	9	State the process for selecting studies (i.e., screening, eligibility, included in systematic review, and, if applicable, included in the meta-analysis).	8-9
Data collection process	10	Describe method of data extraction from reports (e.g., piloted forms, independently, in duplicate) and any processes for obtaining and confirming data from investigators.	10
Definitions for data extraction	11	Provide definitions used in data extraction and classifications of target condition(s), index test(s), reference standard(s) and other characteristics (e.g., study design, clinical setting).	10-11, 39
Risk of bias and applicability	12	Describe methods used for assessing risk of bias in individual studies and concerns regarding the applicability to the review question.	9-10
Diagnostic accuracy measures	13	State the principal diagnostic accuracy measure(s) reported (e.g., sensitivity, specificity) and state the unit of assessment (e.g., per-patient, per-lesion).	10-11, 39
Synthesis of results	12	Describe methods of handling data, combining results of studies and describing variability between studies. This could include, but is not limited to: a) handling of multiple definitions of target condition. b) handling of multiple thresholds of test positivity, c) handling multiple<br>index test readers, d) handling of indeterminate test results, e) grouping and comparing tests, f) handling of different reference standards	11-12
Meta-analysis	D2	Report the statistical methods used for meta-analyses, if performed.	11-12
Additional analyses	16	Describe methods of additional analyses (e.g., sensitivity or subgroup analyses, meta-regression), if done, indicating which were pre-specified.	11-12
**RESULTS**			

Study selection	17	Provide numbers of studies screened, assessed for eligibility, included in the review (and included in meta-analysis, if applicable) with reasons for exclusions at each stage, ideally with a flow diagram.	12-13
Study characteristics	18	For each included study provide citations and present key characteristics including: a) participant characteristics (presentation, prior testing), b) clinical setting, c) study design, d) target condition definition, e) index test, f) reference standard, g) sample size, h) funding sources	13-14, 40-43
Risk of bias in studies	19	Present evaluation of risk of bias and concerns regarding applicability for each study.	14-15
Results of individual studies	20	For each analysis in each study (e.g., unique combination of index test, reference standard, and positivity threshold) report 2x2 data (TP, FP, FN, TN) with estimates of diagnostic accuracy and confidence intervals, ideally with a forest or receiver operator characteristic (ROC) plot.	15-19
Synthesis of results	22	Describe test accuracy, including variability; if meta-analysis was done, include results and confidence intervals.	15-23, 44-45
Additional analysis	23	Give results of additional analyses, if done (e.g., sensitivity or subgroup analyses, meta-regression; analysis of index test: failure rates, proportion of inconclusive results, adverse events).	19-23
**DISCUSSION**			
Summary of evidence	24	Summarize the main findings including the strength of evidence.	15, 24-25, 44-45
Limitations	25	Discuss limitations from included studies (e.g., risk of bias and concerns regarding applicability) and from the review process (e.g., incomplete retrieval of identified research).	30
Conclusions	26	Provide a general interpretation of the results in the context of other evidence. Discuss implications for future research and clinical practice (e.g., the intended use and clinical role of the index test).	31
**FUNDING**			
Funding	26	For the systematic review, describe the sources of funding and other support and the role of the funders.	2

Salameh, Jean-Paul et al. "Preferred reporting items for systematic review and meta-analysis of diagnostic test accuracy studies (PRISMA-DTA): explanation, elaboration, and checklist." *BMJ (Clinical research ed.)* vol. 370 m2632. 14 Aug. 2020, doi:10.1136/bmj.m2632

Table S2. PRISMA 2020 for Abstracts Checklist.

Section and Topic	Item #	Checklist item	Reported on page #
**TITLE**			
Title	1	Identify the report as a systematic review.	1
**BACKGROUND**			
Objectives	2	Provide an explicit statement of the main objective(s) or question(s) the review addresses.	4
**METHODS**			
Eligibility criteria	3	Specify the inclusion and exclusion criteria for the review.	4
Information sources	4	Specify the information sources (e.g. databases, registers) used to identify studies and the date when each was last searched.	4
Risk of bias	5	Specify the methods used to assess risk of bias in the included studies.	4
Synthesis of results	6	Specify the methods used to present and synthesise results.	4
**RESULTS**			
Included studies	7	Give the total number of included studies and participants and summarise relevant characteristics of studies.	5
Synthesis of results	8	Present results for main outcomes, preferably indicating the number of included studies and participants for each. If meta-analysis was done, report the summary estimate and confidence/credible interval. If comparing groups, indicate the direction of the effect (i.e. which group is favoured).	5
**DISCUSSION**			
Limitations of evidence	9	Provide a brief summary of the limitations of the evidence included in the review (e.g. study risk of bias, inconsistency and imprecision).	5
Interpretation	10	Provide a general interpretation of the results and important implications.	5
**OTHER**			
Funding	11	Specify the primary source of funding for the review.	2
Registration	12	Provide the register name and registration number.	4

Table S3. Search strategy in PubMed, Embase, Web of Science, and Cochrane Library.

Database	Search strategy	Filters and Limits	Number of Studies
PubMed	("Macular Degeneration"[Mesh] OR "Macular Degeneration"[Title/Abstract] OR "Macular Degenerations"[Title/Abstract] OR "Macular Dystrophy"[Title/Abstract] OR "Macular Dystrophies"[Title/Abstract] OR "Maculopathy"[Title/Abstract] OR "Maculopathies"[Title/Abstract] OR "age related macular degeneration"[Title/Abstract] OR "age-related macular degeneration"[Title/Abstract] OR "Age-Related Macular Degenerations"[Title/Abstract] OR "senile macular degeneration"[Title/Abstract] OR "Age-Related Maculopathy"[Title/Abstract] OR "Age Related Maculopathy"[Title/Abstract] OR "Age-Related Maculopathies"[Title/Abstract] OR "Age Related Maculopathies"[Title/Abstract] OR "ARMD"[Title/Abstract] OR "AMD"[Title/Abstract])<br>AND<br>("Artificial Intelligence"[Mesh] OR "Machine Learning"[Mesh] OR "Deep Learning"[Mesh] OR "Neural Networks, Computer"[Mesh] OR "Artificial Intelligence"[Title/Abstract] OR "AI"[Title/Abstract] OR "Machine Learning"[Title/Abstract] OR "Deep Learning"[Title/Abstract] OR "Neural Network"[Title/Abstract] OR "CNN"[Title/Abstract] OR "convolutional neural network"[Title/Abstract] OR "radiomics"[Title/Abstract])<br>AND<br>("Tomography, Optical Coherence"[Mesh] OR "optical coherence tomography"[Title/Abstract] OR "OCT"[Title/Abstract] OR "fundus photograph*"[Title/Abstract] OR "retinal photograph*"[Title/Abstract] OR "fundus image*"[Title/Abstract] OR "retinal image*"[Title/Abstract])<br>AND<br>(sensitivity[Title/Abstract] OR specificity[Title/Abstract] OR "sensitivity and specificity"[Mesh] OR "ROC curve"[Mesh] OR "ROC curve"[Title/Abstract] OR "ROC curves"[Title/Abstract] OR "ROC Curves"[Title/Abstract] OR "ROC Analysis"[Title/Abstract] OR "ROC Analyses"[Title/Abstract] OR "Receiver Operating Characteristic"[Title/Abstract] OR "Receiver Operating Characteristics"[Title/Abstract] OR AUC[Title/Abstract] OR "area under the curve"[Title/Abstract] OR accuracy[Title/Abstract] OR "diagnostic accuracy"[Title/Abstract] OR "F1 score"[Title/Abstract] OR "F score"[Title/Abstract] OR recall[Title/Abstract] OR precision[Title/Abstract] OR "diagnos*"[Title/Abstract] OR "classif*"[Title/Abstract] OR "differentiat*"[Title/Abstract])	No restrictions applied regarding date range, language, or publication type.	874
Embase	('macular degeneration'/exp OR 'macular degeneration':ab,ti OR 'macular degenerations':ab,ti OR 'macular dystrophy':ab,ti OR 'macular dystrophies':ab,ti OR 'maculopathy':ab,ti OR 'maculopathies':ab,ti OR 'age related macular degeneration':ab,ti OR 'age-related macular degeneration':ab,ti OR 'age-related macular degenerations':ab,ti OR 'senile macular degeneration':ab,ti OR 'age-related maculopathy':ab,ti OR 'age related maculopathy':ab,ti OR 'age-related maculopathies':ab,ti OR 'age related maculopathies':ab,ti OR 'ARMD':ab,ti OR 'AMD':ab,ti)<br>AND<br>('artificial intelligence'/exp OR 'machine learning'/exp OR 'deep learning'/exp OR 'neural network'/exp OR 'artificial intelligence':ab,ti OR 'AI':ab,ti OR 'machine learning':ab,ti OR 'deep learning':ab,ti OR 'neural network':ab,ti OR 'CNN':ab,ti OR 'convolutional neural network':ab,ti OR 'radiomics':ab,ti)<br>AND<br>('optical coherence tomography'/exp OR 'optical coherence tomography':ab,ti OR 'OCT':ab,ti OR 'fundus photograph*':ab,ti OR 'retinal photograph*':ab,ti OR 'fundus image*':ab,ti OR 'retinal image*':ab,ti)<br>AND<br>('sensitivity':ab,ti OR 'specificity':ab,ti OR 'sensitivity and specificity'/exp OR 'receiver operating characteristic'/exp OR 'ROC curve':ab,ti OR 'ROC curves':ab,ti OR 'ROC Curves':ab,ti OR 'ROC Analysis':ab,ti OR 'ROC Analyses':ab,ti OR 'Receiver Operating Characteristic':ab,ti OR 'Receiver Operating Characteristics':ab,ti OR 'AUC':ab,ti OR 'area under the curve':ab,ti OR	No restrictions applied regarding date range, language, or publication type.	1461

	'accuracy':ab,ti OR 'diagnostic accuracy':ab,ti OR 'F1 score':ab,ti OR 'F score':ab,ti OR 'recall':ab,ti OR 'precision':ab,ti OR 'diagnos*':ab,ti OR 'classif*':ab,ti OR 'differentiat*':ab,ti)		
Web of Science	TS=("Macular Degeneration" OR "Macular Dystrophy" OR "Maculopathy" OR "age related macular degeneration" OR "age-related macular degeneration" OR "senile macular degeneration" OR "Age-Related Maculopathy" OR "ARMD" OR "AMD")<br>AND<br>TS=("Artificial Intelligence" OR "Machine Learning" OR "Deep Learning" OR "Neural Networks, Computer" OR "AI" OR "Neural Network" OR "CNN" OR "convolutional neural network" OR "radiomics")<br>AND<br>TS=("Tomography, Optical Coherence" OR "optical coherence tomography" OR "OCT" OR "fundus photograph*" OR "retinal photograph*" OR "fundus image*" OR "retinal image*")<br>AND<br>TS=(sensitivity OR specificity OR "sensitivity and specificity" OR "ROC curve" OR "ROC Analysis" OR "Receiver Operating Characteristic" OR AUC OR "area under the curve" OR accuracy OR "diagnostic accuracy" OR "F1 score" OR "F score" OR recall OR precision OR diagnos* OR classif* OR differentiat*)	No restrictions applied regarding date range, language, or publication type.	1215
Cochrane Library	("Macular Degeneration" OR "Macular Dystrophy" OR "Maculopathy" OR "age related macular degeneration" OR "age-related macular degeneration" OR "senile macular degeneration" OR "Age-Related Maculopathy" OR "ARMD" OR "AMD"):ti,ab,kw AND ("Artificial Intelligence" OR "Machine Learning" OR "Deep Learning" OR "Neural Networks, Computer" OR "AI" OR "Neural Network" OR "CNN" OR "convolutional neural network" OR "radiomics"):ti,ab,kw AND ("Tomography, Optical Coherence" OR "optical coherence tomography" OR "OCT" OR "fundus photograph" OR "retinal photograph" OR "fundus image" OR "retinal image"):ti,ab,kw AND (sensitivity OR specificity OR "sensitivity and specificity" OR "ROC curve" OR "ROC Analysis" OR "Receiver Operating Characteristic" OR AUC OR "area under the curve" OR accuracy OR "diagnostic accuracy" OR "F1 score" OR "F score" OR “recall” OR “precision” OR “diagnose” OR “classification” OR “differentiation”):ti,ab,kw	No restrictions applied regarding date range, language, or publication type.	36

Table S4. Risk of bias assessment (PROBAST+AI) model development based on seven domains.

Author	Year	Quality				Applicability concerns			Overall judgement	
		Participants and data sources [a]	Predictors [b]	Outcome [c]	Analysis [d]	Participants and data sources [e]	Predictors [f]	Outcome [g]	Quality [h]	Applicability concerns [i]
Abdelhalim et al.[1]	2025	L	L	L	L	L	L	L	L	L
Bao et al.[2]	2025	L	L	L	L	L	L	L	L	L
Durmaz Engin et al.[3]	2025	H	L	L	L	L	L	L	H	L
Zhen et al.[4]	2025	L	L	L	L	L	L	L	L	L
Alenezi et al.[5]	2024	H	L	L	L	L	L	L	H	L
García-Floriano et al.[6]	2024	H	L	U	L	L	L	U	H	U
Le et al.[7]	2024	L	L	L	L	L	L	L	L	L
Oliveira et al.[8]	2024	L	L	L	L	L	L	L	L	L
Wan et al.[9]	2024	U	L	L	L	L	L	L	U	L
Yusufoglu et al. [10]	2024	U	L	L	L	L	L	L	U	L
Celebi et al.[11]	2023	L	L	L	L	L	L	L	L	L
El-Den et al.[12]	2023	L	L	L	L	L	L	L	L	L
Leingang et al.[13]	2023	L	L	L	L	L	L	L	L	L
Chen et al.[14]	2022	L	L	L	L	L	L	L	L	L
He et al.[15]	2022	L	L	L	L	L	L	L	L	L
Skevas et al. [16]	2022	L	L	L	L	L	L	L	L	L
Wang et al.[17]	2022	L	L	L	L	L	L	L	L	L
Tak et al.[18]	2021	L	L	L	L	L	L	L	L	L
Takhchidi et al. [19]	2021	L	L	L	L	L	L	L	L	L
Thomas et al.[20]	2021	L	L	U	L	L	L	U	U	U
Heo et al.[21]	2020	L	L	L	L	L	L	L	L	L
Zapata et al.[22]	2020	L	L	L	L	L	L	L	L	L
Bhatia et al.[23]	2019	U	L	H	L	L	L	L	H	L
Matsuba et al. [24]	2019	L	L	L	L	L	L	L	L	L
Yoo et al.[25]	2019	L	L	L	L	L	L	L	L	L
Grassmann et al.[26]	2018	L	L	L	L	L	L	L	L	L
Tan et al.[27]	2018	L	L	L	L	L	L	L	L	L
Lee et al.[28]	2017	L	L	L	L	L	L	L	L	L

Abbreviation: PROBAST+AI, Prediction model Risk of Bias Assessment Tool + AI, L low; H high; U unclear.

Footnote: Signaling questions are rated as "yes" (Y), "probably yes" (PY), "probably no" (PN), "no" (N), "no information" (NI), and in some cases "not applicable" (NA). All signaling questions are phrased in such a way that "yes" or "probably yes" indicates a low risk of bias. Any signaling questions rated as "no" or "probably no" indicate a potential high risk of bias in that domain. If there are no "no" or "probably no" ratings, but "no information" (NI) is present, the risk of bias in that domain is classified as unclear.

a. Participants and data sources

1.1 Did the in- and exclusions of study participants result in a representative dataset?

b. Predictors

2.1 Were predictors defined and assessed in a similar way for all participants?

2.2 Was any pre-processing of predictors similar for all participants?

c. Outcome

3.1 Were outcomes defined and assessed appropriately?

3.2 Were outcomes defined and assessed in a similar way for all participants?

3.3 Were outcome assessments made without use or knowledge of predictor data?

d. Analysis

4.1 Was there evidence that the sample size was reasonable?

4.2 Were continuous and categorical predictors handled appropriately?

4.3 Were participants with missing or censored data handled appropriately in the analysis?

4.4 Were methods used to address potential model overfitting?

e. Participants and data sources

Concern that the (data of the) included participants do not match the review question or the assessor’s intended use of the prediction model.

f. Predictors

Concern that the definition, pre-processing, assessment, or timing of assessment of the predictors in the model do not match the review question or the assessor’s intended use.

g. Outcome

Concern that the outcome, its definition, assessment, or timing of assessment do not match the review question or the assessor’s intended use.

h. Quality

Low risk: If all four domains were rated low concern regarding quality.

High risk: If at least one domain was rated high concern regarding quality.

Unclear: If at least one domain was rated unclear concern regarding quality and no domains were rated high concern.

i. Applicability concerns

Low risk: If all three domains were rated low concern for applicability.

High risk: If at least one domain was rated high concern for applicability.

Unclear: If at least one domain was rated unclear concern for applicability and no domains were rated high concern.

Table S5. Risk of bias assessment (PROBAST+AI) model evaluation based on seven domains.

Author	Year	Risk of bias				Applicability concerns			Overall judgement	
		Participants and data sources [a]	Predictors [b]	Outcome [c]	Analysis [d]	Participants and data sources [e]	Predictors [f]	Outcome [g]	Risk of bias [h]	Applicability concerns [i]
Abdelhalim et al.[1]	2025	L	L	L	L	L	L	L	L	L
Bao et al.[2]	2025	L	L	L	L	L	L	L	L	L
Durmaz Engin et al.[3]	2025	H	L	U	L	L	L	L	H	L
Zhen et al.[4]	2025	L	L	L	L	L	L	L	L	L
Alenezi et al. [5]	2024	H	L	L	L	L	L	L	H	L
García-Floriano et al.[6]	2024	H	L	U	H	L	L	U	H	U
Le et al.[7]	2024	L	L	L	L	L	L	L	L	L
Oliveira et al.[8]	2024	L	L	L	L	L	L	L	L	L
Wan et al.[9]	2024	U	L	U	L	L	L	L	U	L
Yusufoglu et al.[10]	2024	U	L	U	L	L	L	L	U	L
Celebi et al.[11]	2023	L	L	U	L	L	L	L	U	L
El-Den et al.[12]	2023	L	L	L	L	L	L	L	L	L
Leingang et al.[13]	2023	L	L	U	H	L	L	L	H	L
Chen et al.[14]	2022	L	L	L	L	L	L	L	L	L
He et al.[15]	2022	L	L	U	L	L	L	L	U	L
Skevas et al.[16]	2022	L	L	L	L	L	L	L	L	L
Wang et al.[17]	2022	L	L	L	L	L	L	L	L	L
Tak et al.[18]	2021	L	L	L	H	L	L	L	H	L
Takhchidi et al.[19]	2021	L	L	L	L	L	L	L	L	L
Thomas et al.[20]	2021	L	L	U	H	L	L	U	H	U
Heo et al.[21]	2020	L	L	L	L	L	L	L	L	L
Zapata et al.[22]	2020	L	L	L	L	L	L	L	L	L
Bhatia et al.[23]	2019	U	L	H	L	L	L	L	H	L
Matsuba et al.[24]	2019	L	L	L	L	L	L	L	L	L
Yoo et al.[25]	2019	L	L	L	L	L	L	L	L	L
Grassmann et al.[26]	2018	L	L	L	L	L	L	L	L	L
Tan et al.[27]	2018	L	L	L	L	L	L	L	L	L
Lee et al.[28]	2017	L	L	L	L	L	L	L	L	L

Abbreviation: PROBAST+AI, Prediction model Risk of Bias Assessment Tool + AI, L low; H high; U unclear.

Footnote: Signaling questions are rated as "yes" (Y), "probably yes" (PY), "probably no" (PN), "no" (N), "no information" (NI), and in some cases "not applicable" (NA). All signaling questions are phrased in such a way that "yes" or "probably yes" indicates a low risk of bias. Any signaling questions rated as "no" or "probably no" indicate a potential high risk of bias in that domain. If there are no "no" or "probably no" ratings, but "no information" (NI) is present, the risk of bias in that domain is classified as unclear.

a. Participants and data sources

1.1 Did the in- and exclusions of study participants result in a representative dataset?

b. Predictors

2.1 Were predictors defined and assessed in a similar way for all participants?

2.2 Was any pre-processing of predictors similar for all participants?

2.3 Were predictor assessments made without knowledge of outcome data?

c. Outcome

3.1 Were outcomes defined and assessed appropriately?

3.2 Were outcomes defined and assessed in a similar way for all participants?

3.3 Were outcome assessments made without use or knowledge of predictor data?

d. Analysis

4.1 Was there evidence that the sample size was reasonable?

4.2 Were participants with missing or censored data handled appropriately in the analysis?

4.3 If data splitting was done to create training and test datasets, was there evidence that data leakage was avoided?

4.4 If resampling methods were used to evaluate model performance, were all model development steps replicated in the resampling process?

4.5 Was the predictive performance of the model evaluated appropriately, e.g., calibration, discrimination, and net benefit?

e. Participants and data sources

Concern that the (data of the) included participants do not match the review question or the assessor's intended use of the prediction model.

f. Predictors

Concern that the definition, pre-processing, assessment, or timing of assessment of the predictors in the model do not match the review question or the assessor's intended use.

g. Outcome

Concern that the outcome, its definition, assessment, or timing of assessment do not match the review question or the assessor's intended use.

h. Risk of bias

Low risk: If all four domains were rated low risk of bias.

High risk: If at least one domain was rated high risk of bias.

Unclear: If at least one domain was rated unclear risk of bias and no domains were rated high risk of bias.

i. Applicability concerns

Low risk: If all three domains were rated low concern for applicability.

High risk: If at least one domain was rated high concern for applicability.

Unclear: If at least one domain was rated unclear concern for applicability and no domains were rated high concern.

Table S6. Detailed GRADE assessment criteria and certainty judgments for all pooled outcomes.

Dataset	Outcome	Risk of Bias [a]	Inconsistency [b]	Indirectness [c]	Imprecision [d]	Small-study effects e	Total Downgrade	Final Rating
DL for AMD vs. normal	Sensitivity	1	0	0	0	0	1	Moderate
	Specificity	1	0	0	0	0	1	Moderate
	Accuracy	1	0	0	0	0	1	Moderate
DL for wAMD vs. dAMD	Sensitivity	1	0	0	0	0	1	Moderate
	Specificity	1	0	0	0	0	1	Moderate
	Accuracy	1	0	0	0	0	1	Moderate

AMD age-related macular degeneration; wAMD wet age-related macular degeneration; dAMD dry age-related macular degeneration; DL deep learning.

a. Risk of bias: Downgraded by 1 level for all pooled outcomes. PROBAST+AI judged 14% (4/28) of studies as high risk in model development and 25% (7/28) as high risk in validation/testing; most studies were retrospective, several relied mainly on internal validation, and reporting of patient-level separation or reference standards was incomplete in some studies. These issues could overestimate diagnostic performance.

b. Inconsistency: No additional downgrade was applied. Statistical heterogeneity was substantial, but it was explored using subgroup analysis, meta-regression, bivariate boxplots, and sensitivity analyses; the direction of effect remained consistently high across the main diagnostic tasks, and the prediction intervals remained within clinically high-performance ranges. This was treated as a borderline GRADE judgment and is noted for transparency.

c. Indirectness: No downgrade was applied because the included studies addressed the prespecified review question in population, index test, target condition, reference standard, and diagnostic task. However, diagnostic accuracy is a surrogate for patient-important outcomes; direct evidence on visual outcomes, referral burden, treatment timing, or clinical workflow after AI-assisted testing was unavailable.

d. Imprecision: No downgrade was applied because pooled 95% confidence intervals for sensitivity and specificity were narrow and did not cross prespecified clinically important thresholds for diagnostic performance.

e. Small-study effects: No downgrade was applied because Deeks' funnel plot asymmetry tests did not suggest significant small-study effects for either diagnostic task (AMD vs normal, *P*=.26; wAMD vs dAMD, *P*=.18).

Table S7. Technical aspects of age-related macular degeneration (AMD) versus normal in the included studies.

Author	Year	Type of imaging	Data source	AI model	Major AI algorithms [a]	Data splitting method	OCT Data Types
Abdelhalim et al.[1]	2025	CFP	Open database	Image only model	CNN	Train/Validation/Test	B-scan
Bao et al.[2]	2025	CFP	Private database & Open database	Image only model	CNN	Hold out & Independent dataset validation	NA
Durmaz Engin et al.[3]	2025	OCT	Open database	Image only model	CNN	Hold out	B-scan
Zhen et al.[4]	2025	OCT & CFP	Open database	Image only model	Transformer	Train (5-fold cross-validation)/Validation/Test	Volume scan
Alenezi et al.[5]	2024	OCT	Open database	Image only model	CNN	5-fold cross-validation	B-scan
García-Floriano et al.[6]	2024	CFP	Open database	Image only model	CNN	10-fold cross-validation	NA
Le et al.[7]	2024	CFP	Private database & Open database	Image only model	Transformer	5-fold cross -validation & Independent dataset validation	NA
Oliveira et al.[8]	2024	CFP	Open database	Image only model	CNN	Hold out & Independent dataset validation	NA
Wan et al.[9]	2024	CFP	Private database & Open database	Image only model	CNN & Transformer	Hold out & Independent dataset validation	NA
Yusufoglu et al.[10]	2024	OCT	Private database & Open database	Image only model	CNN	Train/Validation/Test	B-scan
Celebi et al.[11]	2023	OCT	Private database & Open database	Image only model	CapsNet	Hold out	B-scan
El-Den et al.[12]	2023	CFP	Private database	Image only model	CNN	Train/Validation/Test	NA
Leingang et al.[13]	2023	OCT	Private database	Image only model	Two-Stage CNN	Hold out	Volume scan
Chen et al.[14]	2022	OCT	Private database	Image only model	CNN	Hold out	B-scan
He et al.[15]	2022	OCT	Open database	Image only model	CNN	Hold out & Independent dataset validation	B-scan
Skevas et al.[16]	2022	CFP	Private database	Image only model	CNN	Independent dataset validation	NA
Wang et al.[17]	2022	OCT & CFP	Private database	Image only model	Two-Stream CNN	Train/Validation/Test	B-scan
Takhchidi et al.[19]	2021	CFP	Private database	Image only model	Faster R-CNN	Hold out	NA

Thomas et al.[20]	2021	OCT	Open database	Image only model	CNN	Hold out & Independent dataset validation	B-scan
Heo et al.[21]	2020	CFP	Private database	Image only model	CNN	5-fold cross-validation	NA
Zapata et al.[22]	2020	OCT & CFP	Private database	Image only model	CNN	Train/Validation/Test	B-scan
Bhatia et al.[23]	2019	OCT	Private database & Open database	Image only model	CNN	Independent dataset validation	B-scan
Matsuba et al.[24]	2019	CFP	Private database	Image only model	D-CNN	Hold out	NA
Yoo et al.[25]	2019	OCT & CFP	Open database	Image only model	CNN & RF	Hold out & Independent dataset validation	B-scan
Grassmann et al.[26]	2018	CFP	Private database	Image only model	CNN & RF	Train/Validation/Test & Independent dataset validation	NA
Tan et al.[27]	2018	CFP	Private database	Image only model	D-CNN	10-fold cross-validation	NA
Lee et al.[28]	2017	OCT	Private database	Image only model	CNN	Hold out	Volume scan

TP true positive; TN true negative; FP false positive; FN false negative; NA not available; AI artificial intelligence; RF random forest; CNN convolutional neural network; D-CNN deep convolutional neural network; R-CNN region-based convolutional neural network; CapsNet capsule network; OCT optical coherence tomography; CFP color fundus photography.

[a] The major AI algorithm means the algorithm with the highest AUC value or defined by the authors.

Table S8. Diagnostic performance of age-related macular degeneration (AMD) versus normal in the included studies.

Author	Year	Type of imaging	Internal validation						External validation					
			TP	FP	FN	TN	Accuracy	AUC	TP	FP	FN	TN	Accuracy	AUC
Abdelhalim et al.[1]	2025	CFP	71	1	3	52	0.97 (123/127)	NA	NA	NA	NA	NA	NA	NA
Bao et al.[2]	2025	CFP	92	2	9	120	0.95 (212/223)	NA	136	112	58	1089	0.88 (1225/1395)	NA
			NA	NA	NA	NA	NA	NA	38	0	2	19	0.97 (57/59)	NA
Durmaz Engin et al.[3]	2025	OCT	200	1	0	99	1.00 (299/300)	NA	NA	NA	NA	NA	NA	NA
Zhen et al.[4]	2025	OCT & CFP	101	0	0	33	1.00 (134/134)	NA	NA	NA	NA	NA	NA	NA
Alenezi et al.[5]	2024	OCT	361	31	6	156	0.93 (517/554)	NA	NA	NA	NA	NA	NA	NA
García-Floriano et al.[6]	2024	CFP	NA	NA	NA	NA	NA	NA	11	2	0	9	0.91 (11/13)	NA
Le et al.[7]	2024	CFP	1095	15	49	1200	0.97 (2295/2359)	NA	440	44	60	206	0.86 (646/750)	NA
Oliveira et al.[8]	2024	CFP	90	NA	15	NA	NA	NA	30	5	10	35	0.81 (65/80)	NA
Wan et al.[9]	2024	CFP	NA	NA	NA	NA	NA	NA	60	0	0	40	1.00 (100/100)	NA
Yusufoglu et al.[10]	2024	OCT	213	0	1	133	1.00 (346/347)	NA	NA	NA	NA	NA	NA	NA
			248	0	0	243	1.00 (491/491)	NA	NA	NA	NA	NA	NA	NA
Celebi et al.[11]	2023	OCT	1085	0	18	638	0.99 (1723/1741)	NA	NA	NA	NA	NA	NA	NA
			17369	0	6	7970	1.00 (25339/25345)	NA	NA	NA	NA	NA	NA	NA
El-Den et al.[12]	2023	CFP	97	1	1	29	0.98 (126/128)	NA	NA	NA	NA	NA	NA	NA
Leingang et al.[13]	2023	OCT	87	1	3	5	0.96 (92/96)	NA	NA	NA	NA	NA	NA	NA
Chen et al.[14]	2022	OCT	88	3	1	61	0.97 (149/153)	NA	143	8	19	44	0.87 (187/214)	NA
He et al.[15]	2022	OCT	500	1	0	249	1.00 (749/750)	1.00	687	16	36	1391	0.98 (2078/2130)	0.99
Skevas et al.[16]	2022	CFP	NA	NA	NA	NA	NA	NA	68	115	1	414	0.81 (482/598)	NA
Wang et al.[17]	2022	OCT & CFP	71	0	0	20	1.00 (91/91)	NA	NA	NA	NA	NA	NA	NA
Takhchidi et al.[19]	2021	CFP	99	6	1	100	0.97 (199/206)	NA	NA	NA	NA	NA	NA	NA
Thomas et al.[20]	2021	OCT	499	1	1	249	1.00 (748/750)	0.99	14	0	1	15	0.97 (29/30)	1.00
Heo et al.[21]	2020	CFP	181	7	10	81	0.94 (262/279)	NA	NA	NA	NA	NA	NA	NA
Zapata et al.[22]	2020	OCT & CFP	972	199	110	927	0.86 (1899/2208)	0.93	NA	NA	NA	NA	NA	NA
Bhatia et al.[23]	2019	OCT	NA	NA	NA	NA	NA	NA	46	2	2	48	0.96 (94/98)	0.99
			NA	NA	NA	NA	NA	NA	50	2	0	23	0.97 (73/75)	0.99

Matsuba et al.[24]	2019	CFP	42	2	0	67	0.98 (109/111)	0.99	NA	NA	NA	NA	NA	NA
Yoo et al.[25]	2019	OCT & CFP	573	22	27	278	0.95 (851/900)	0.98	48	10	8	17	0.78 (65/83)	NA
Grassmann et al.[26]	2018	CFP	6792	323	779	3724	0.91 (10516/11618)	NA	123	71	97	1386	0.90 (1509/1677)	NA
Tan et al.[27]	2018	CFP	675	25	25	375	0.95 (1050/1100)	NA	NA	NA	NA	NA	NA	NA
Lee et al.[28]	2017	OCT	9921	528	1695	8019	0.89 (17940/20163)	0.93	NA	NA	NA	NA	NA	NA

TP true positive; TN true negative; FP false positive; FN false negative; NA not available; OCT optical coherence tomography; CFP color fundus photography.

Table S9. Technical aspects of wet age-related macular degeneration (wAMD) versus dry age-related macular degeneration (dAMD) in the included studies.

Author	Year	Type of imaging	Data source	AI model	Optimal AI algorithms [a]	Data splitting method	OCT Data Types
Bao et al.[2]	2025	CFP	Private database & Open database	Image only model	CNN	Hold out & Independent dataset validation	NA
Durmaz Engin et al.[3]	2025	OCT	Open database	Image only model	CNN	Hold out	B-scan
Zhen et al.[4]	2025	OCT & CFP	Open database	Image only model	Transformer	Train (5-fold cross-validation)/Validation/Test	Volume scan
Alenezi et al.[5]	2024	OCT	Open database	Image only model	CNN	5-fold cross-validation	B-scan
Le et al.[7]	2024	CFP	Private database & Open database	Image only model	Transformer	5-fold cross -validation & Independent dataset validation	NA
Wan et al.[9]	2024	CFP	Private database & Open database	Image only model	CNN & Transformer	Hold out & Independent dataset validation	NA
Yusufoglu et al.[10]	2024	OCT	Private database & Open database	Image only model	CNN	Train/Validation/Test	B-scan
Celebi et al.[11]	2023	OCT	Private database & Open database	Image only model	CapsNet	Hold out	B-scan
El-Den et al.[12]	2023	CFP	Private database	Image only model	CNN	Train/Validation/Test	NA
Chen et al.[14]	2022	OCT	Private database	Image only model	CNN	Hold out	B-scan
Wang et al.[17]	2022	OCT & CFP	Private database	Image only model	Two-Stream CNN	Train/Validation/Test	B-scan
Tak et al.[18]	2021	CFP	Private database	Image only model	CNN	Hold out	NA
Heo et al.[21]	2020	CFP	Private database	Image only model	CNN	5-fold cross-validation	NA
Bhatia et al.[23]	2019	OCT	Private database & Open database	Image only model	CNN	Independent dataset validation	B-scan
Yoo et al.[25]	2019	OCT & CFP	Open database	Image only model	CNN & RF	Hold out & Independent dataset validation	B-scan
Grassmann et al.[26]	2018	CFP	Private database	Image only model	CNN & RF	Train/Validation/Test & Independent dataset validation	NA

TP true positive; TN true negative; FP false positive; FN false negative; NA not available; AI artificial intelligence; RF random forest; CNN convolutional neural network; D-CNN deep convolutional neural network; R-CNN region-based convolutional neural network; CapsNet capsule network; OCT optical coherence tomography; CFP color fundus photography.

[a] The optimal AI algorithm means the algorithm with the highest AUC value.

Table S10. Diagnostic performance of wet age-related macular degeneration (wAMD) versus dry age-related macular degeneration (dAMD) in the included studies.

Author	Year	Type of imaging	Internal validation						External validation					
			TP	FP	FN	TN	Accuracy	AUC	TP	FP	FN	TN	Accuracy	AUC
Bao et al.[2]	2025	CFP	57	1	3	31	0.96 (88/92)	NA	31	13	5	87	0.87 (118/136)	NA
			NA	NA	NA	NA	NA	NA	16	2	5	17	0.82 (33/40)	NA
Durmaz Engin et al.[3]	2025	OCT	100	0	0	100	1.00 (200/200)	NA	NA	NA	NA	NA	NA	NA
Zhen et al.[4]	2025	OCT & CFP	75	0	0	26	1.00 (101/101)	NA	NA	NA	NA	NA	NA	NA
Alenezi et al.[5]	2024	OCT	170	5	3	183	0.98 (353/361)	NA	NA	NA	NA	NA	NA	NA
Le et al.[7]	2024	CFP	532	37	56	470	0.92 (1002/1095)	NA	160	11	79	190	0.80 (350/440)	NA
Wan et al.[9]	2024	CFP	NA	NA	NA	NA	NA	NA	41	1	0	18	0.98 (59/60)	NA
Yusufoglu et al.[10]	2024	OCT	109	5	1	98	0.97 (207/213)	NA	NA	NA	NA	NA	NA	NA
Celebi et al.[11]	2023	OCT	333	9	12	731	0.98 (1064/1085)	NA	NA	NA	NA	NA	NA	NA
			11039	168	195	5967	0.98 (17006/17369)	NA	NA	NA	NA	NA	NA	NA
El-Den et al.[12]	2023	CFP	30	0	2	65	0.98 (95/97)	NA	NA	NA	NA	NA	NA	NA
Chen et al.[14]	2022	OCT	41	2	6	38	0.91 (79/87)	NA	76	5	20	42	0.83 (118/143)	NA
Wang et al.[17]	2022	OCT & CFP	33	6	0	32	0.92 (65/71)	NA	NA	NA	NA	NA	NA	NA
Tak et al.[18]	2021	CFP	26	3	2	41	0.93 (67/72)	NA	NA	NA	NA	NA	NA	NA
Heo et al.[21]	2020	CFP	87	5	12	87	0.91 (174/191)	NA	NA	NA	NA	NA	NA	NA
Bhatia et al.[23]	2019	OCT	NA	NA	NA	NA	NA	NA	24	1	1	24	0.96 (48/50)	0.98
Yoo et al.[25]	2019	OCT & CFP	NA	NA	NA	NA	NA	NA	33	2	3	10	0.90 (43/48)	NA
Grassmann et al.[26]	2018	CFP	1202	209	230	4990	0.93 (6192/6631)	NA	4	25	0	94	0.80 (98/123)	NA

TP true positive; TN true negative; FP false positive; FN false negative; NA not available; OCT optical coherence tomography; CFP color fundus photography.

Table S11. Diagnostic data for different level of ophthalmologists.

Author	Year	Type of imaging	Target of conditions	Year of experience	Junior ophthalmologists					Year of experience	Senior ophthalmologists				
					TP	FP	FN	TN	Accuracy		TP	FP	FN	TN	Accuracy
Bao et al.[2]	2025	CFP	AMD vs. normal	NA	36	2	4	17	0.90 (53/59)	NA	31	0	9	19	0.85 (50/59)
Bao et al.[2]	2025	CFP	wAMD vs. AMD	NA	18	9	3	10	0.70 (28/40)	NA	14	0	7	19	0.83 (33/40)
Oliveira et al.[8]	2024	CFP	AMD vs. normal	NA	NA	NA	NA	NA	NA	NA	8	2	2	8	0.80 (16/20)
Heo et al.[21]	2020	CFP	wAMD vs. AMD	1	NA	NA	NA	NA	0.76 (146/191)	4	NA	NA	NA	NA	0.89 (170/191)
Matsuba et al.[24]	2019	CFP	AMD vs. normal	NA	NA	NA	NA	NA	NA	NA	30	3	12	39	0.82 (69/84)

TP true positive; TN true negative; FP false positive; FN false negative; NA not available; AMD age-related macular degeneration; wAMD wet age-related macular degeneration; dAMD dry age-related macular degeneration; OCT optical coherence tomography; CFP color fundus photography.

Table S12. Subgroup analysis and meta-regression analysis for age-related macular degeneration (AMD) vs. normal.

Subgroup	Number of validation data sets, n	Sensitivity(95%CI)	Meta-regression P-value	Specificity(95%CI)	Meta-regression P-value
**Validation**			< 0.001		< 0.001
Internal validation	23	0.99 (0.97 - 1.00)		0.98 (0.97 - 0.99)	
External validation	14	0.93 (0.88 - 0.99)		0.94 (0.89 - 0.99)	
**Type of imaging**			0.90		0.13
Unimodal	32	0.97 (0.96 - 0.99)		0.97 (0.96 - 0.99)	
Multimodal	5	0.98 (0.94 - 1.00)		0.94 (0.86 - 1.00)	
**Database**			< 0.001		< 0.001
Open database	17	0.98 (0.96 - 1.00)		0.97 (0.95 - 1.00)	
Private database	20	0.97 (0.94 - 0.99)		0.97 (0.94 - 0.99)	
**Analysis**			0.45		0.83
Eye-based analysis	5	0.99 (0.96 - 1.00)		0.96 (0.90 - 1.00)	
Image-based analysis	28	0.97 (0.95 - 0.99)		0.97 (0.96 - 0.99)	
**Centers**			< 0.001		< 0.001
Single center	21	0.98 (0.97 - 1.00)		0.98 (0.97 - 1.00)	
Multi-center	13	0.94 (0.89 - 0.99)		0.94 (0.89 - 0.99)	

AI artificial intelligence.

Table S13. Subgroup analysis and meta-regression analysis for wet age-related macular degeneration (wAMD) vs. dry age-related macular degeneration (dAMD).

Subgroup	Number of validation data sets, n	Sensitivity(95%CI)	Meta-regression P-value	Specificity(95%CI)	Meta-regression P-value
**Validation**			< 0.001		< 0.001
Internal validation	14	0.96 (0.94-0.99)		0.97 (0.95 - 0.98)	
External validation	8	0.89 (0.80 - 0.97)		0.90 (0.85 - 0.95)	
**Type of imaging**			0.07		0.03
Unimodal	19	0.94 (0.90 - 0.97)		0.96 (0.94 - 0.98)	
Multimodal	3	0.99 (0.96 - 1.00)		0.91 (0.81 - 1.00)	
**Databases**			< 0.001		< 0.001
Open database	8	0.97 (0.94 - 1.00)		0.97 (0.94 - 0.99)	
Private database	14	0.93 (0.88 - 0.97)		0.95 (0.92 - 0.97)	
**Analysis**			0.64		0.22
Eye-based analysis	4	0.93 (0.83 - 1.00)		0.92 (0.85 - 0.99)	
Image-based analysis	16	0.94 (0.91 - 0.98)		0.96 (0.94 - 0.98)	
**Centers**			< 0.001		< 0.001
Single center	14	0.95 (0.92 - 0.98)		0.95 (0.92 - 0.97)	
Multi-center	6	0.87 (0.77 - 0.97)		0.95 (0.91 - 0.98)	

NA not available.

Table S14. Sensitivity analysis of deep learning algorithms for classifying age-related macular degeneration (AMD) from normal under different exclusion criteria.

Analysis Scenario	No. of studies (validation sets)	Sensitivity (95%CI)	Specificity (95%CI)	Accuracy (95%CI)	AUC (95%CI)
Overall	27(77485)	0.98 (0.96 – 0.99)	0.98 (0.95 - 0.99)	0.97 (0.96 - 0.99)	1.00 (0.99 – 1.00)
Excluding outliers and influential studies [a]	27(52140)	0.97 (0.95 - 0.99)	0.97 (0.95 - 0.98)	0.97 (0.95 - 0.98)	0.99 (0.98 – 1.00)
Without low-quality studies [b]	21(75560)	0.97 (0.95 - 0.99)	0.98 (0.95 - 0.99)	0.97 (0.95 - 0.99)	1.00 (0.99 – 1.00)

CI confidence interval; AUC area under the curve.

[a] Outliers and influential points were identified based on the intersection of standardized residual plots, bivariate boxplots, and Cook's distance plots. Studies excluded: Celebi et al.[11] (data from the open database).

[b] Low-quality means that the study was rated as having at least one high-risk item in the PROBAST+AI tool (validation sets). Excluded Durmaz Engin et al.[3], Alenezi et al.[5], García-Floriano et al.[6], Leingang et al.[13], Thomas et al.[20], Bhatia et al.[23].

Table S15. Sensitivity analysis of deep learning algorithms for classifying wet age-related macular degeneration (wAMD) from dry age-related macular degeneration (dAMD) under different exclusion criteria.

Analysis Scenario	No. of studies (validation sets)	Sensitivity (95%CI)	Specificity (95%CI)	Accuracy (95%CI)	AUC (95%CI)
Overall	16(28705)	0.95 (0.91 – 0.97)	0.95 (0.93 - 0.97)	0.95 (0.92 - 0.97)	0.99 (0.97 – 0.99)
Without low-quality studies [a]	12 (28022)	0.93 (0.88 - 0.96)	0.95 (0.92 - 0.97)	0.94 (0.90 - 0.96)	0.98 (0.97 – 0.99)

CI confidence interval; AUC area under the curve.

[a] Low-quality means that the study was rated as having at least one high-risk item in the PROBAST+AI tool (validation sets). Excluded Durmaz Engin et al.[3], Alenezi et al.[5], Tak et al.[18], Bhatia et al.[23].

Note: An analysis excluding outliers and influential studies was performed using the same criteria as in Table 13 (intersection of standardized residuals, bivariate boxplots, and Cook's distance). However, no studies in the wAMD vs. dAMD dataset met the criteria for exclusion, so this analysis scenario is identical to the "Overall" scenario and is not listed separately.

Figure S1. Graphical assessment of model fit, bivariate normality, and influence diagnostics for deep learning algorithms classifying age-related macular degeneration (AMD) from normal.

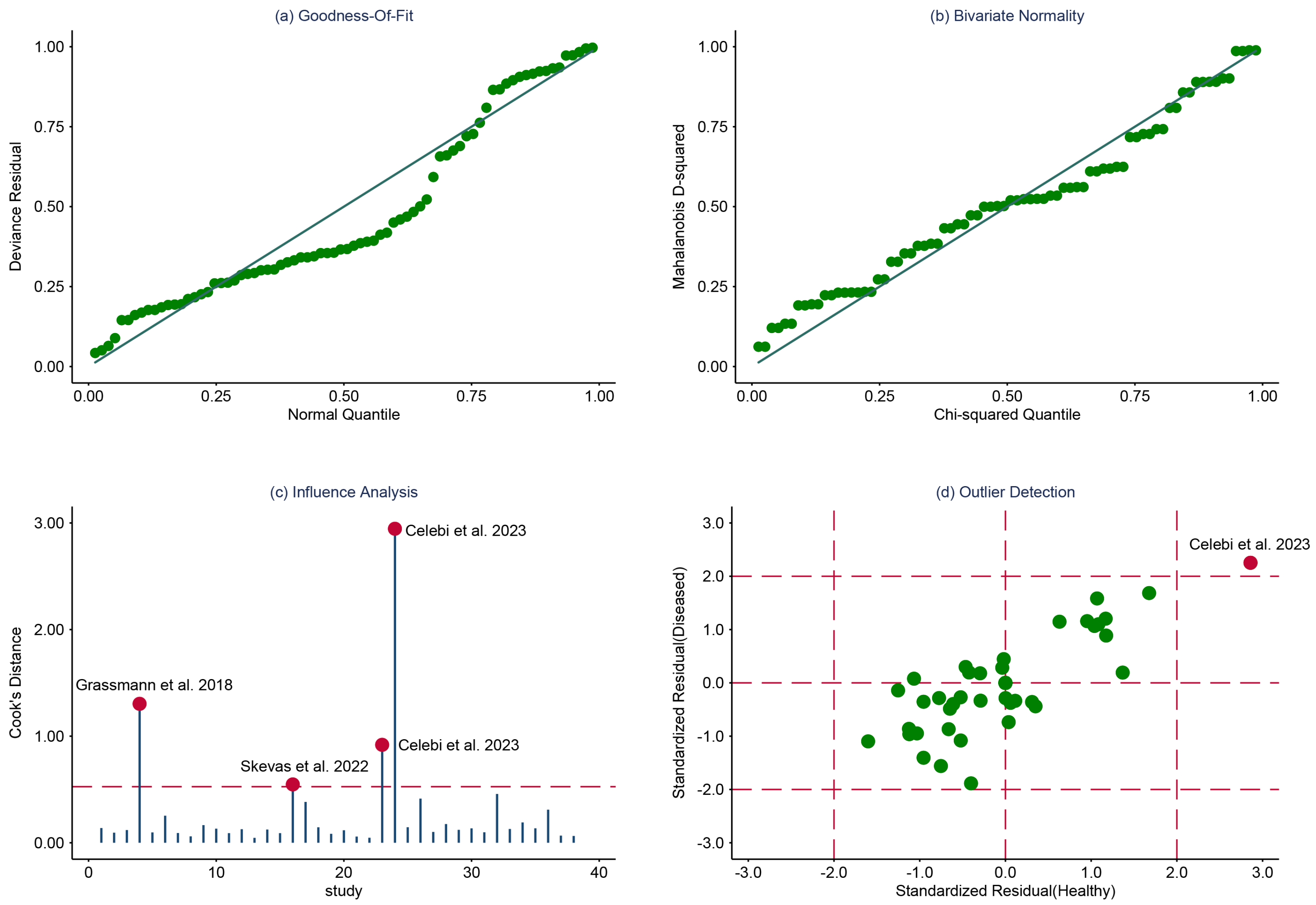

Figure S2. Graphical assessment of model fit, bivariate normality, and influence diagnostics for deep learning algorithms classifying wet age-related macular degeneration (wAMD) from dry age-related macular degeneration (dAMD).

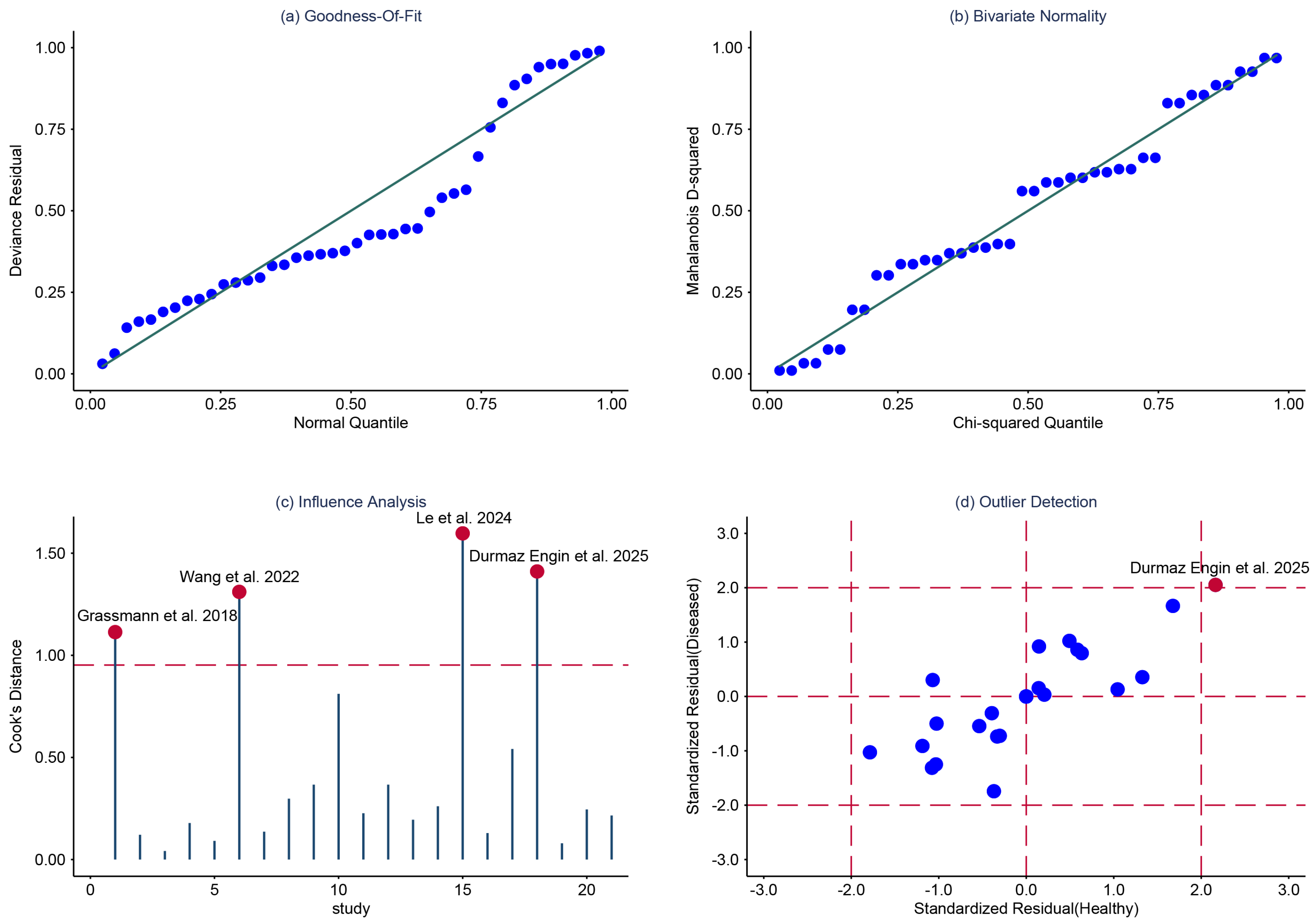

Figure S3. Forest plots of the pooled accuracy of deep learning algorithms using generalized linear mixed model (GLMM) for classifying age-related macular degeneration (AMD) from normal.

Author,year	Events	Total	GLMM, Accuracy, 95% CI
Abdelhalim et al. 2025	123	127	0.97 [0.92 - 0.99]
Bao et al. 2025	212	223	0.95 [0.91 - 0.98]
Bao et al. 2025	1225	1395	0.88 [0.86 - 0.89]
Bao et al. 2025	57	59	0.97 [0.88 - 1.00]
Durmaz Engin et al. 2025	299	300	1.00 [0.98 - 1.00]
Zhen et al. 2025	134	134	1.00 [0.97 - 1.00]
Alenezi et al. 2024	517	554	0.93 [0.91 - 0.95]
García−Floriano et al. 2024	20	22	0.91 [0.71 - 0.99]
Le et al. 2024	2295	2359	0.97 [0.97 - 0.98]
Le et al. 2024	646	750	0.86 [0.83 - 0.89]
Oliveira et al. 2024	65	80	0.81 [0.71 - 0.89]
Wan et al. 2024	100	100	1.00 [0.96 - 1.00]
Yusufoglu et al. 2024	346	347	1.00 [0.98 - 1.00]
Yusufoglu et al. 2024	491	491	1.00 [0.99 - 1.00]
Celebi et al. 2023	1723	1741	0.99 [0.98 - 0.99]
Celebi et al. 2023	25339	25345	1.00 [1.00 - 1.00]
El−Den et al. 2023	126	128	0.98 [0.94 - 1.00]
Leingang et al. 2023	92	96	0.96 [0.90 - 0.99]
Chen et al. 2022	149	153	0.97 [0.93 - 0.99]
Chen et al. 2022	187	214	0.87 [0.82 - 0.92]
He et al. 2022	749	750	1.00 [0.99 - 1.00]
He et al. 2022	2078	2130	0.98 [0.97 - 0.98]
Skevas et al. 2022	482	598	0.81 [0.77 - 0.84]
Wang et al. 2022	91	91	1.00 [0.96 - 1.00]
Takhchidi et al. 2021	199	206	0.97 [0.93 - 0.99]
Thomas et al. 2021	748	750	1.00 [0.99 - 1.00]
Thomas et al. 2021	29	30	0.97 [0.83 - 1.00]
Heo et al. 2020	262	279	0.94 [0.90 - 0.96]
Zapata et al. 2020	1899	2208	0.86 [0.84 - 0.87]
Bhatia et al. 2019	94	98	0.96 [0.90 - 0.99]
Bhatia et al. 2019	73	75	0.97 [0.91 - 1.00]
Matsuba et al. 2019	109	111	0.98 [0.94 - 1.00]
Yoo et al. 2019	851	900	0.95 [0.93 - 0.96]
Yoo et al. 2019	65	83	0.78 [0.68 - 0.87]
Grassmann et al. 2018	10516	11618	0.91 [0.90 - 0.91]
Grassmann et al. 2018	1509	1677	0.90 [0.88 - 0.91]
Tan et al. 2018	1050	1100	0.95 [0.94 - 0.97]
Lee et al. 2017	17940	20163	0.89 [0.89 - 0.89]
**Total (95% CI)**		**77485**	**0.97 [0.96 - 0.99]**

Heterogeneity: $Tau^2 = 2.8530$; $Chi^2 = 914.66$, df = 37 ($P < 0.0001$); $I^2 = 96.0\%$

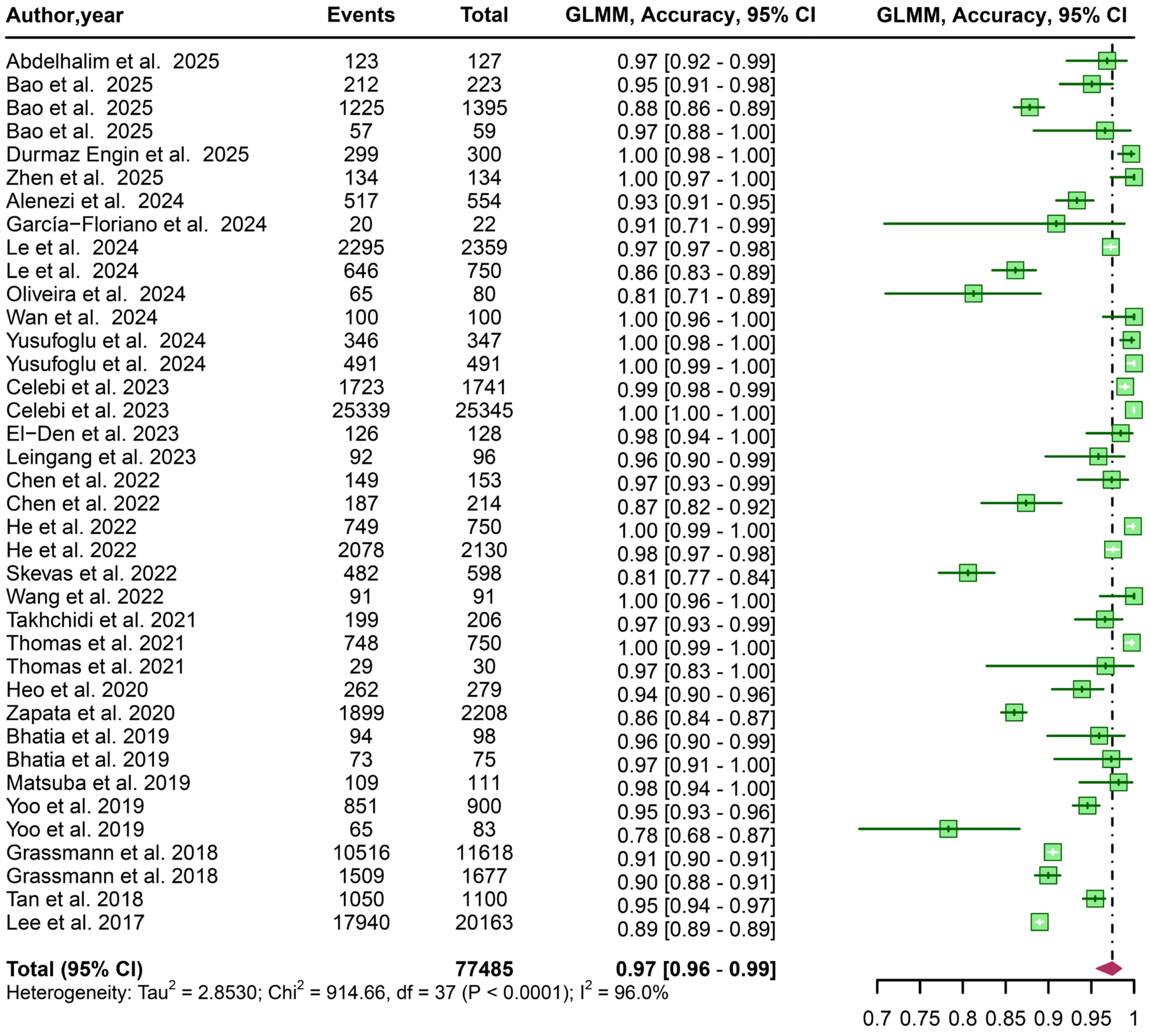

Figure S4. Summary receiver operating characteristic (SROC) curve of deep learning algorithms for classifying age-related macular degeneration (AMD) from normal.

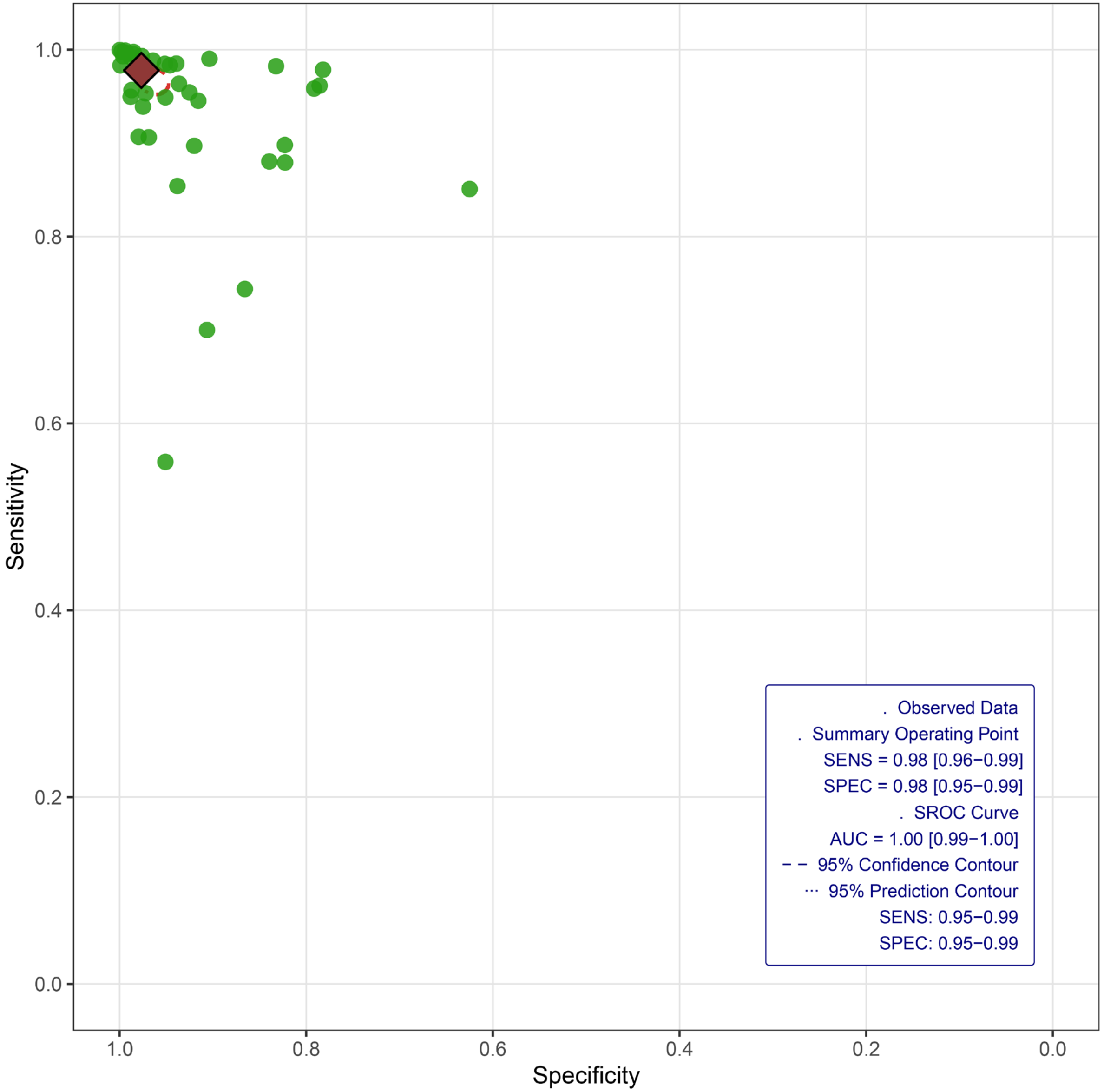

Figure S5. Forest plots of the pooled sensitivity and specificity of deep learning algorithms of junior and senior ophthalmologists for classifying age-related macular degeneration (AMD) from normal and wet age-related macular degeneration (wAMD) from dry age-related macular degeneration (dAMD).

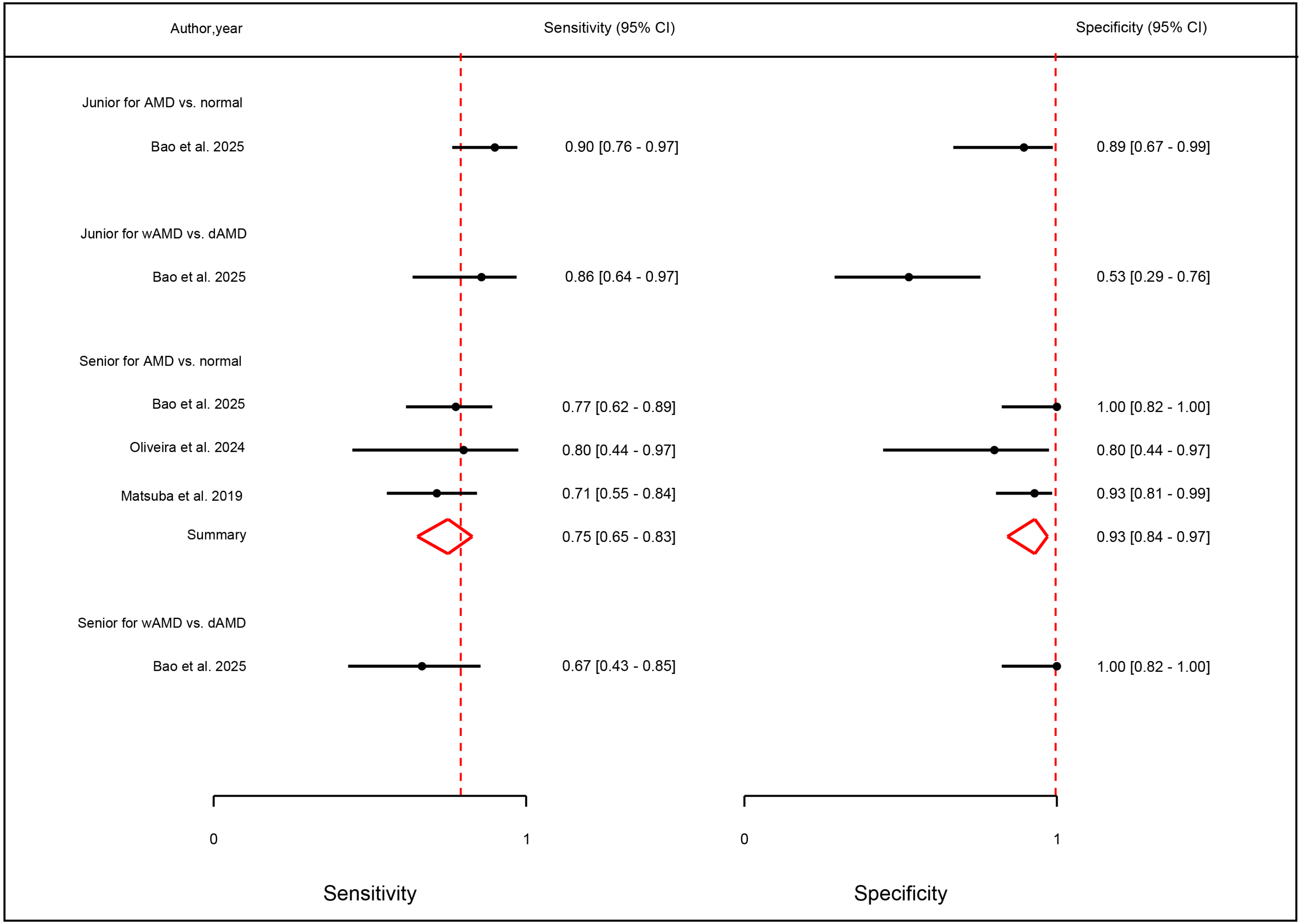

Figure S6. Forest plots of the pooled accuracy of deep learning algorithms using generalized linear mixed model (GLMM) of junior and senior ophthalmologists for classifying age-related macular degeneration (AMD) from normal and wet age-related macular degeneration (wAMD) from dry age-related macular degeneration (dAMD).

Experience<br>Author,year	Events	Total	GLMM, Accuracy, 95% CI
**experience = Junior for AMD vs. normal**			
Bao et al. 2025	53	59	0.90 [0.79 - 0.96]
**experience = Junior for wAMD vs.dAMD**			
Bao et al. 2025	28	40	0.70 [0.53 - 0.83]
Heo et al. 2020	146	191	0.76 [0.70 - 0.82]
**Total (95% CI)**		**231**	**0.75 [0.69 - 0.80]**
Heterogeneity: $Tau^2 = 0$; $Chi^2 = 0.73$, df = 1 (P = 0.3917); $I^2 = 0\%$			
**experience = Senior for AMD vs. normal**			
Bao et al. 2025	50	59	0.85 [0.73 - 0.93]
Oliveira et al. 2024	16	20	0.80 [0.56 - 0.94]
Matsuba et al. 2019	69	84	0.82 [0.72 - 0.90]
**Total (95% CI)**		**163**	**0.83 [0.76 - 0.88]**
Heterogeneity: $Tau^2 = 0$; $Chi^2 = 0.29$, df = 2 (P = 0.8643); $I^2 = 0\%$			
**experience = Senior for wAMD vs.dAMD**			
Bao et al. 2025	33	40	0.82 [0.67 - 0.93]
Heo et al. 2020	170	191	0.89 [0.84 - 0.93]
**Total (95% CI)**		**231**	**0.88 [0.83 - 0.91]**
Heterogeneity: $Tau^2 = 0$; $Chi^2 = 1.29$, df = 1 (P = 0.2561); $I^2 = 22.5\%$			
**Total (95% CI)**		**684**	**0.83 [0.78 - 0.87]**
Heterogeneity: $Tau^2 = 0.0960$; $Chi^2 = 16.60$, df = 7 (P = 0.0202); $I^2 = 57.8\%$			
Test for subgroup differences: $Chi^2 = 14.62$, df = 3 (P = 0.0022)			

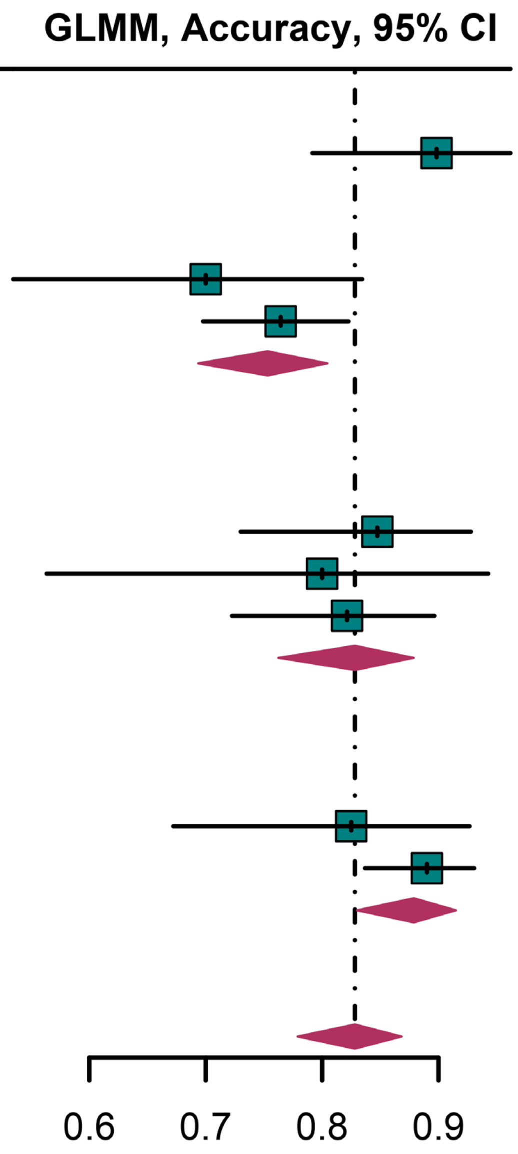

Figure S7. Forest plots of the pooled accuracy of deep learning algorithms using generalized linear mixed model (GLMM) for classifying wet age-related macular degeneration (wAMD) from dry age-related macular degeneration (dAMD).

Author,year	Events	Total	GLMM, Accuracy, 95% CI
Bao et al. 2025	88	92	0.96 [0.89 - 0.99]
Bao et al. 2025	118	136	0.87 [0.80 - 0.92]
Bao et al. 2025	33	40	0.82 [0.67 - 0.93]
Durmaz Engin et al. 2025	200	200	1.00 [0.98 - 1.00]
Zhen et al. 2025	101	101	1.00 [0.96 - 1.00]
Alenezi et al. 2024	353	361	0.98 [0.96 - 0.99]
Le et al. 2024	1002	1095	0.92 [0.90 - 0.93]
Le et al. 2024	350	440	0.80 [0.75 - 0.83]
Wan et al. 2024	59	60	0.98 [0.91 - 1.00]
Yusufoglu et al. 2024	207	213	0.97 [0.94 - 0.99]
Celebi et al. 2023	1064	1085	0.98 [0.97 - 0.99]
Celebi et al. 2023	17006	17369	0.98 [0.98 - 0.98]
El−Den et al. 2023	95	97	0.98 [0.93 - 1.00]
Chen et al. 2022	79	87	0.91 [0.83 - 0.96]
Chen et al. 2022	118	143	0.83 [0.75 - 0.88]
Wang et al. 2022	65	71	0.92 [0.83 - 0.97]
Tak et al. 2021	67	72	0.93 [0.85 - 0.98]
Heo et al. 2020	174	191	0.91 [0.86 - 0.95]
Bhatia et al. 2019	48	50	0.96 [0.86 - 1.00]
Yoo et al. 2019	43	48	0.90 [0.77 - 0.97]
Grassmann et al. 2018	6192	6631	0.93 [0.93 - 0.94]
Grassmann et al. 2018	98	123	0.80 [0.71 - 0.86]
**Total (95% CI)**		**28705**	**0.95 [0.92 - 0.97]**

Heterogeneity: $Tau^2 = 1.0681$; $Chi^2 = 695.37$, df = 21 ($P < 0.0001$); $I^2 = 97.0\%$

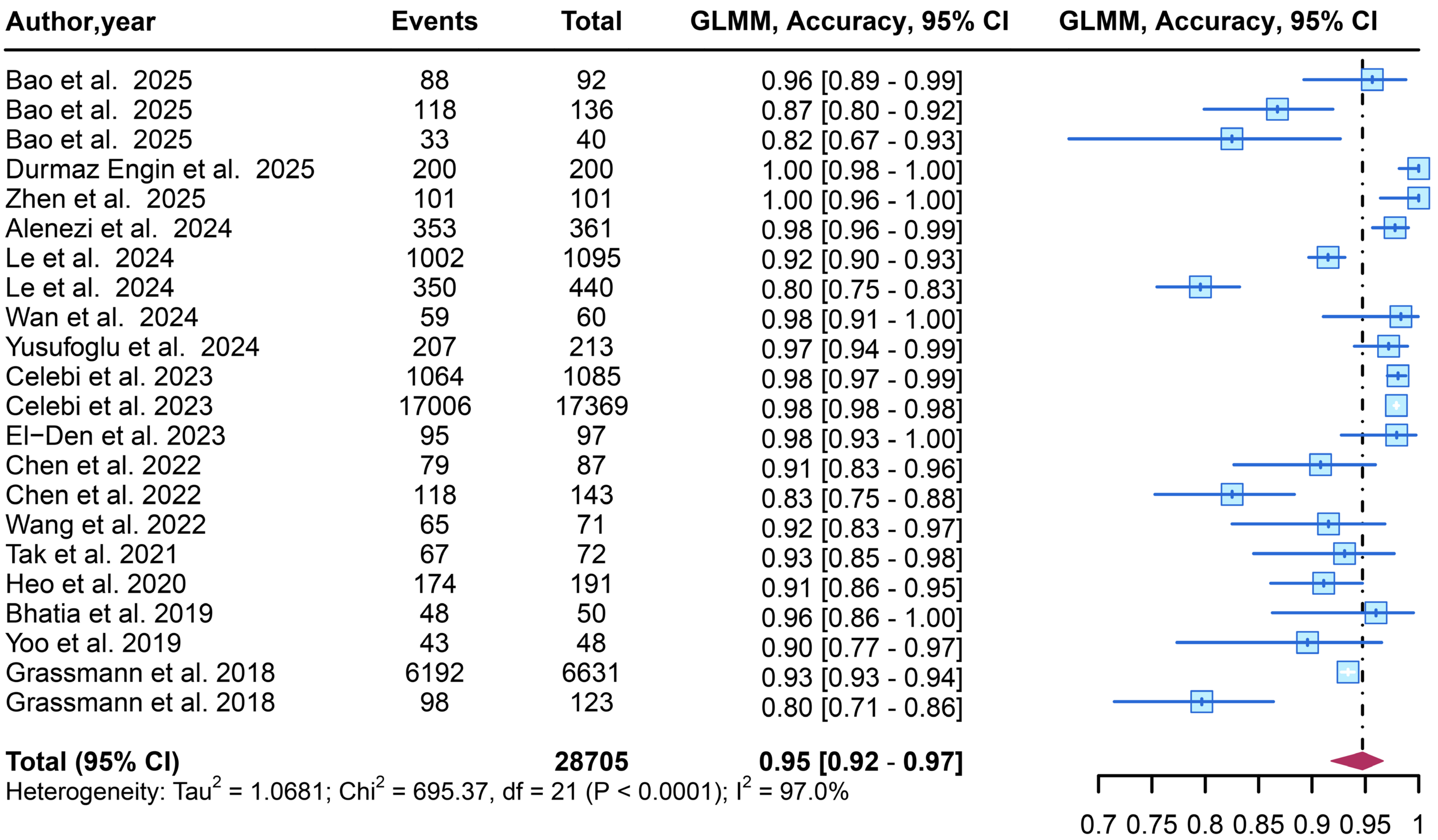

Figure S8. Summary receiver operating characteristic (SROC) curve of deep learning algorithms for classifying wet age-related macular degeneration (wAMD) from dry age-related macular degeneration (dAMD).

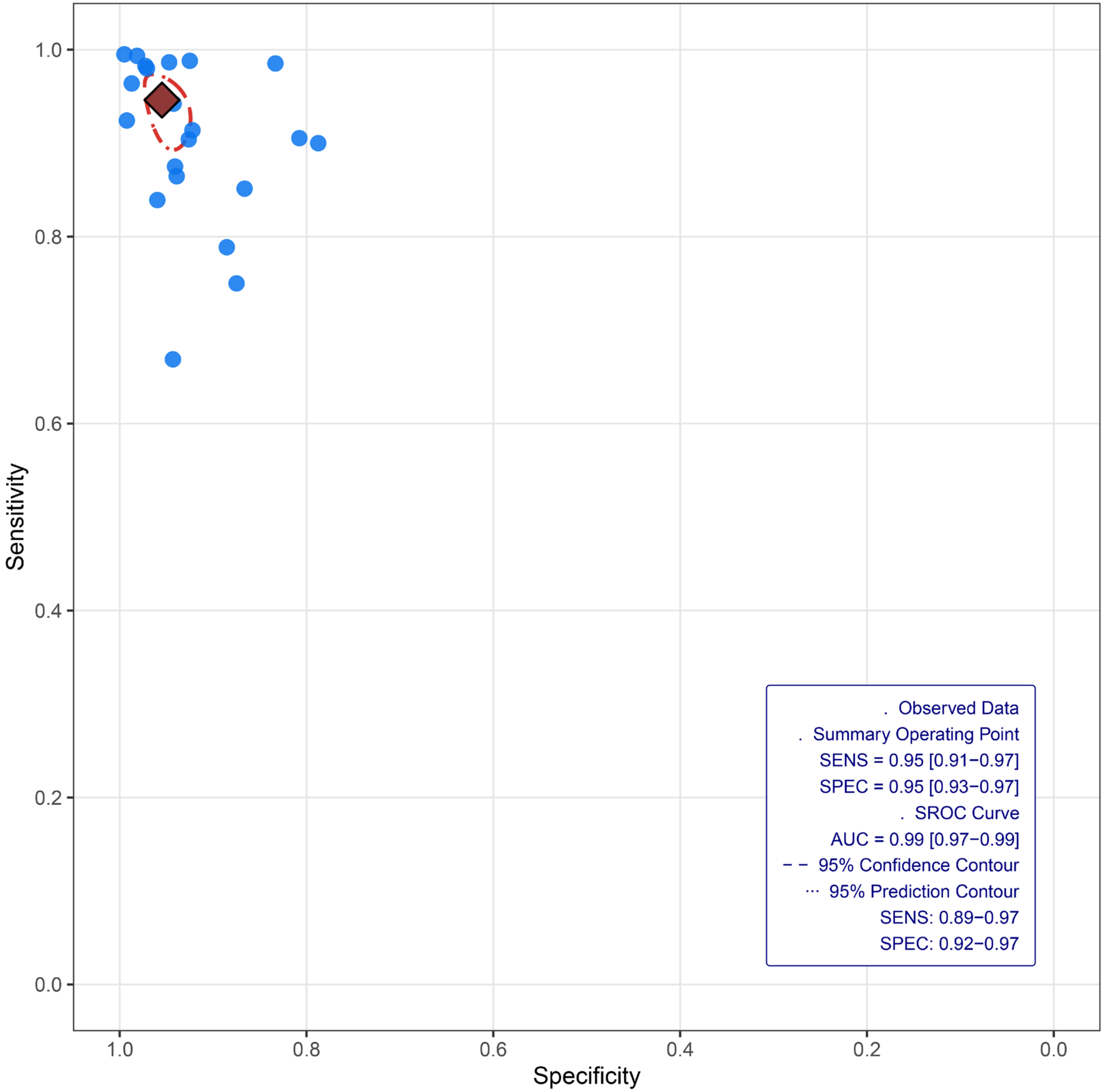

Figure S9. Forest plots of the pooled sensitivity and specificity of deep learning algorithms for classifying age-related macular degeneration (AMD) from normal based on optical coherence tomography (OCT) images.

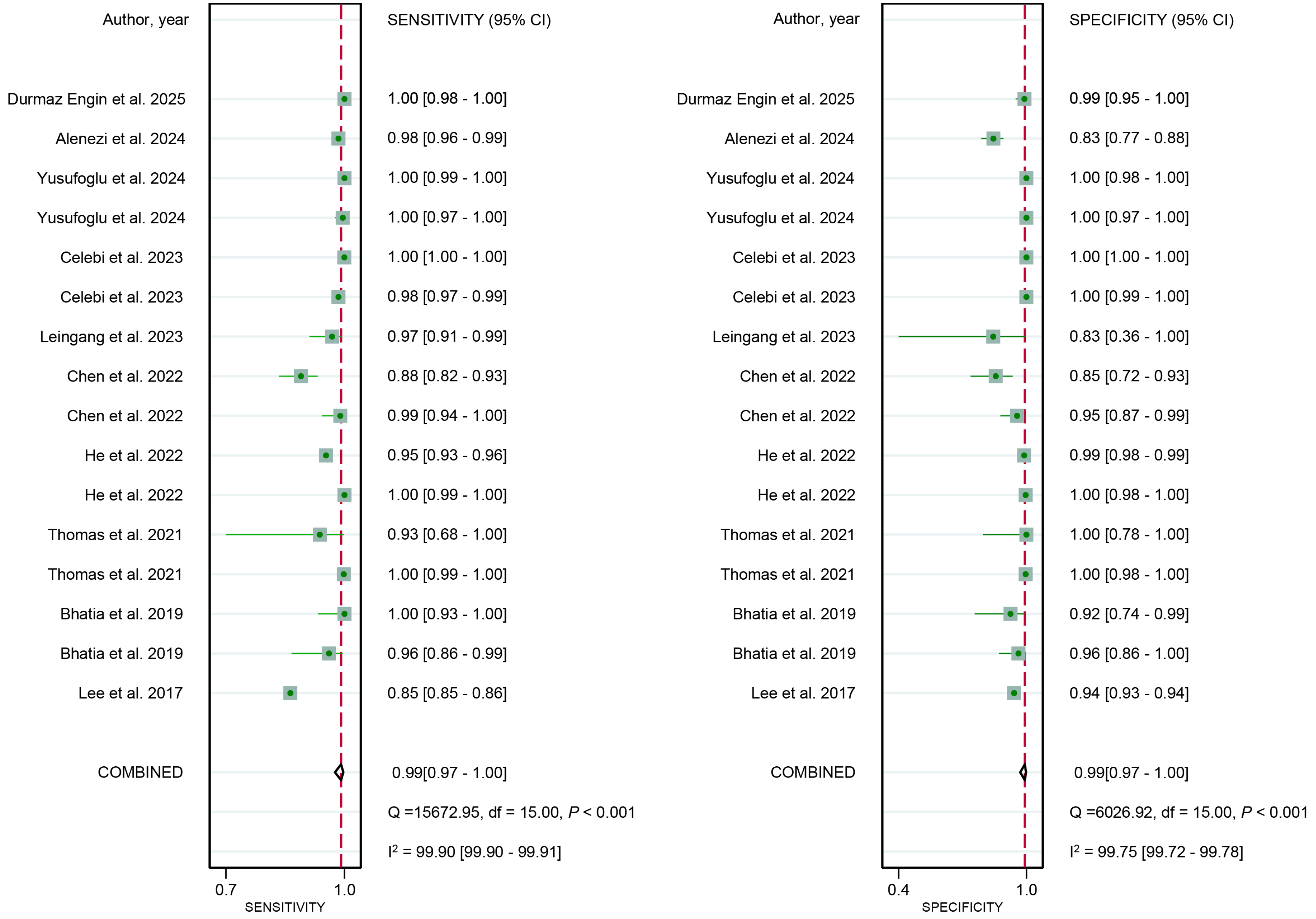

Figure S10. Forest plots of the pooled accuracy of deep learning algorithms using generalized linear mixed model (GLMM) for classifying age-related macular degeneration (AMD) from normal based on optical coherence tomography (OCT) images.

Author, year	Events	Total	GLMM, Accuracy, 95% CI
Durmaz Engin et al. 2025	299	300	1.00 [0.98 - 1.00]
Alenezi et al. 2024	517	554	0.93 [0.91 - 0.95]
Yusufoglu et al. 2024	346	347	1.00 [0.98 - 1.00]
Yusufoglu et al. 2024	491	491	1.00 [0.99 - 1.00]
Celebi et al. 2023	1723	1741	0.99 [0.98 - 0.99]
Celebi et al. 2023	25339	25345	1.00 [1.00 - 1.00]
Leingang et al. 2023	92	96	0.96 [0.90 - 0.99]
Chen et al. 2022	149	153	0.97 [0.93 - 0.99]
Chen et al. 2022	187	214	0.87 [0.82 - 0.92]
He et al. 2022	749	750	1.00 [0.99 - 1.00]
He et al. 2022	2078	2130	0.98 [0.97 - 0.98]
Thomas et al. 2021	748	750	1.00 [0.99 - 1.00]
Thomas et al. 2021	29	30	0.97 [0.83 - 1.00]
Bhatia et al. 2019	94	98	0.96 [0.90 - 0.99]
Bhatia et al. 2019	73	75	0.97 [0.91 - 1.00]
Lee et al. 2017	17940	20163	0.89 [0.89 - 0.89]
**Total (95% CI)**		**53237**	**0.99 [0.97 - 1.00]**

Heterogeneity: $Tau^2 = 3.5072$; $Chi^2 = 563.61$, df = 15 ($P < 0.0001$); $I^2 = 97.3\%$

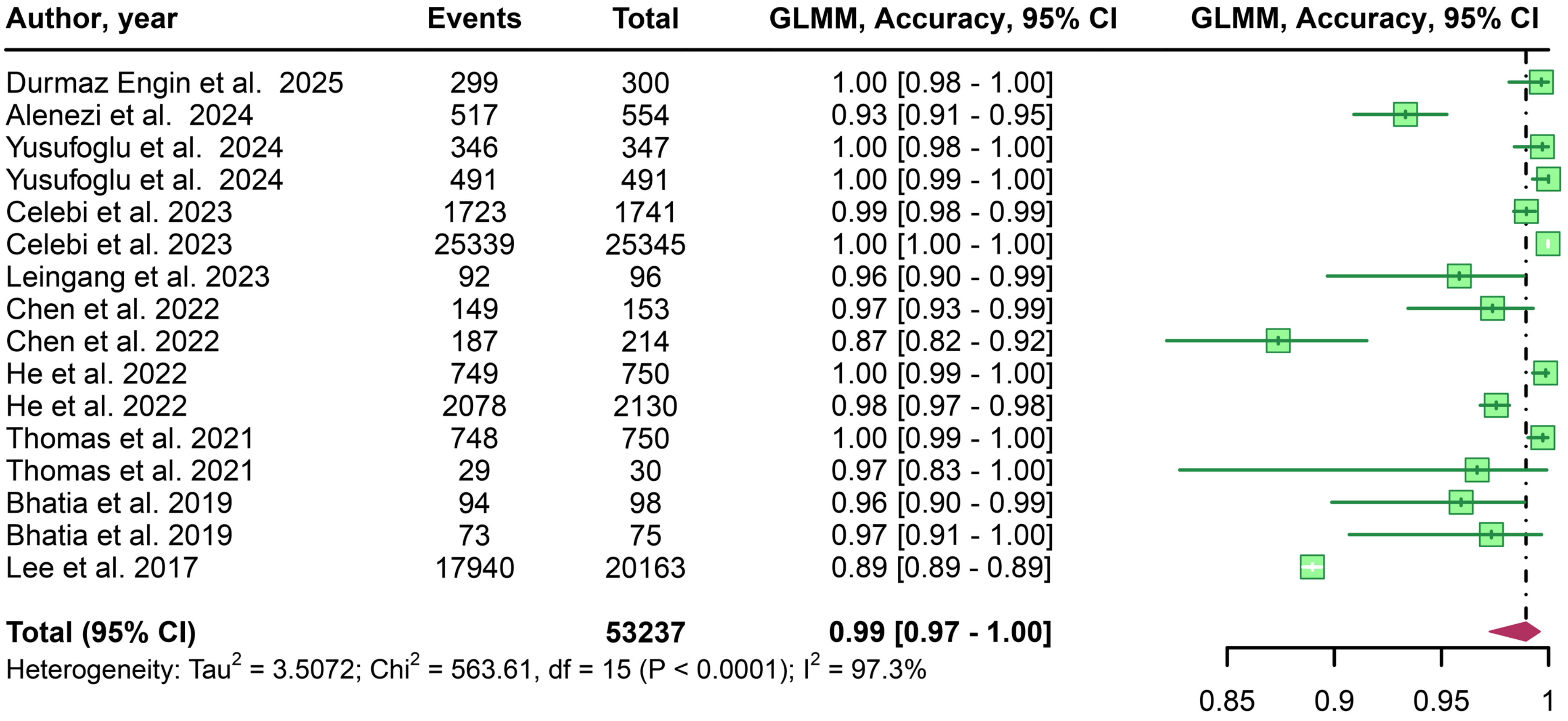

Figure S11. Summary receiver operating characteristic (SROC) curve of deep learning algorithms for classifying age-related macular degeneration (AMD) from normal based on optical coherence tomography (OCT) images.

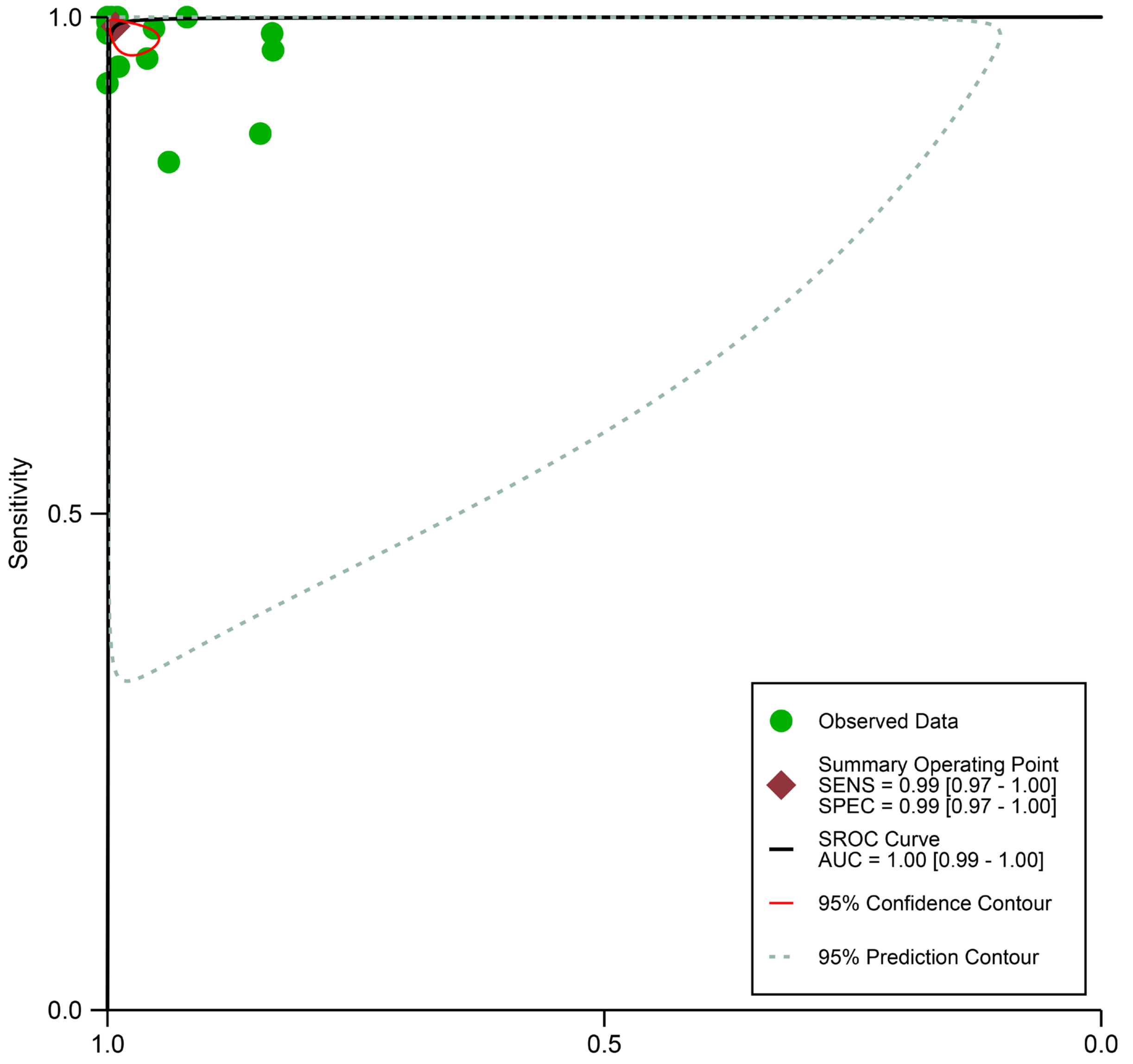

Figure S12. Forest plots of the pooled sensitivity and specificity of deep learning algorithms for classifying age-related macular degeneration (AMD) from normal based on color fundus photography (CFP) images.

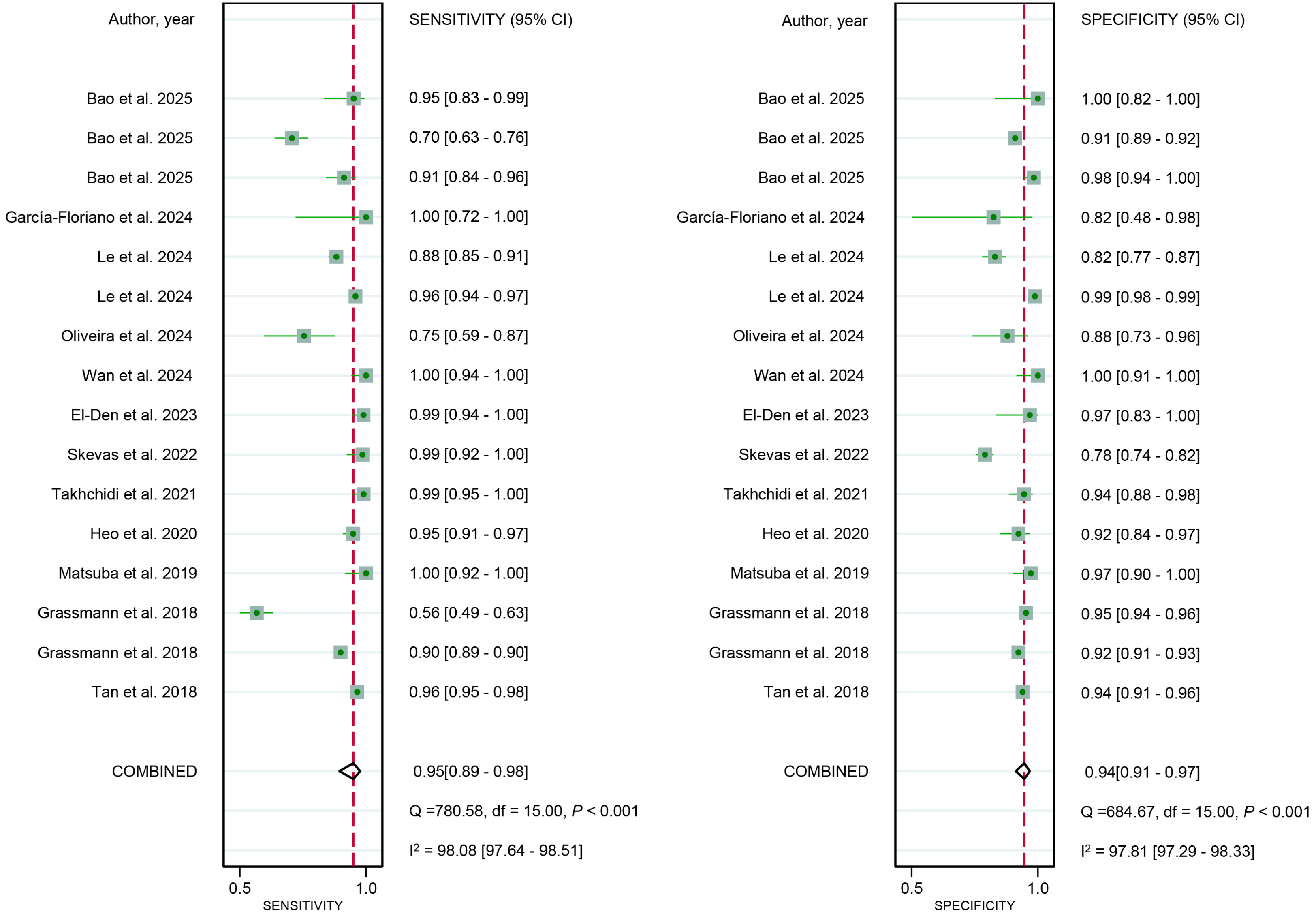

Figure S13. Forest plots of the pooled accuracy of deep learning algorithms using generalized linear mixed model (GLMM) for classifying age-related macular degeneration (AMD) from normal based on color fundus photography (CFP) images.

Author, year	Events	Total	GLMM, Accuracy, 95% CI
Bao et al. 2025	212	223	0.95 [0.91 - 0.98]
Bao et al. 2025	1225	1395	0.88 [0.86 - 0.89]
Bao et al. 2025	57	59	0.97 [0.88 - 1.00]
García−Floriano et al. 2024	20	22	0.91 [0.71 - 0.99]
Le et al. 2024	2295	2359	0.97 [0.97 - 0.98]
Le et al. 2024	646	750	0.86 [0.83 - 0.89]
Oliveira et al. 2024	65	80	0.81 [0.71 - 0.89]
Wan et al. 2024	100	100	1.00 [0.96 - 1.00]
El−Den et al. 2023	126	128	0.98 [0.94 - 1.00]
Skevas et al. 2022	482	598	0.81 [0.77 - 0.84]
Takhchidi et al. 2021	199	206	0.97 [0.93 - 0.99]
Heo et al. 2020	262	279	0.94 [0.90 - 0.96]
Matsuba et al. 2019	109	111	0.98 [0.94 - 1.00]
Grassmann et al. 2018	10516	11618	0.91 [0.90 - 0.91]
Grassmann et al. 2018	1509	1677	0.90 [0.88 - 0.91]
Tan et al. 2018	1050	1100	0.95 [0.94 - 0.97]
**Total (95% CI)**		**20705**	**0.94 [0.91 - 0.96]**

Heterogeneity: $Tau^2 = 0.7296$; $Chi^2 = 271.69$, df = 15 (P < 0.0001); $I^2 = 94.5\%$

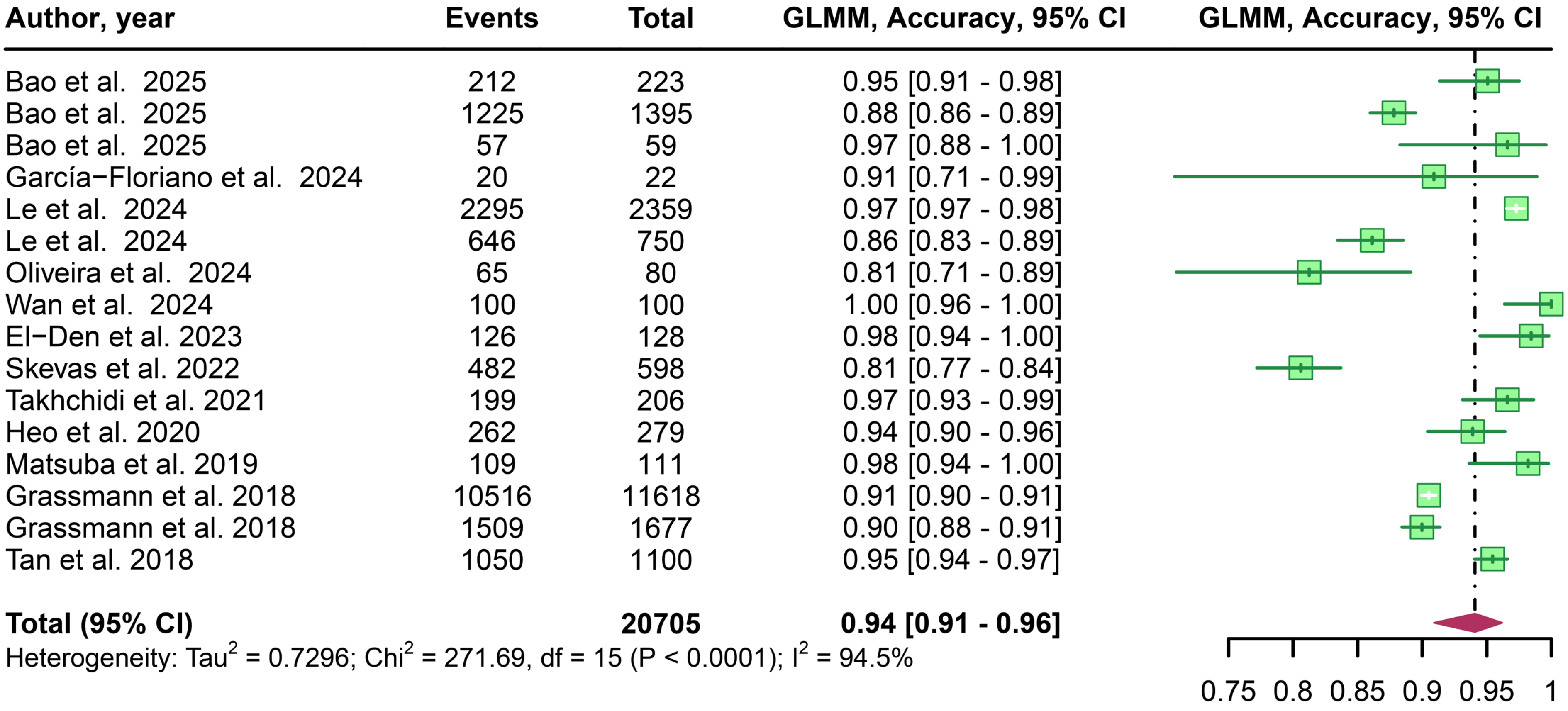

Figure S14. Summary receiver operating characteristic (SROC) curve of deep learning algorithms for classifying age-related macular degeneration (AMD) from normal based on color fundus photography (CFP) images.

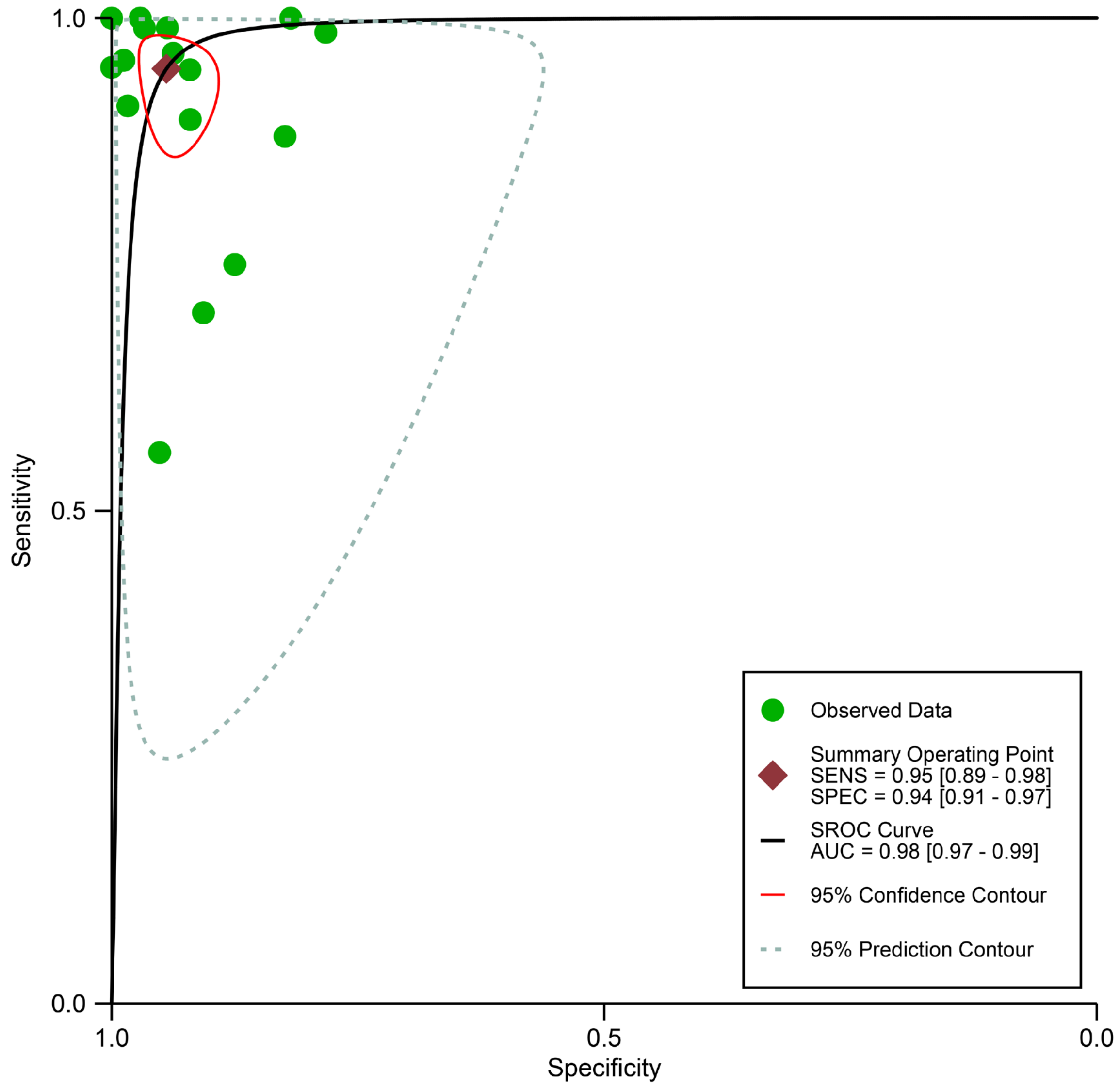

Figure S15. Forest plots of the pooled sensitivity and specificity of deep learning algorithms for classifying age-related macular degeneration (AMD) from normal based on multimodal images.

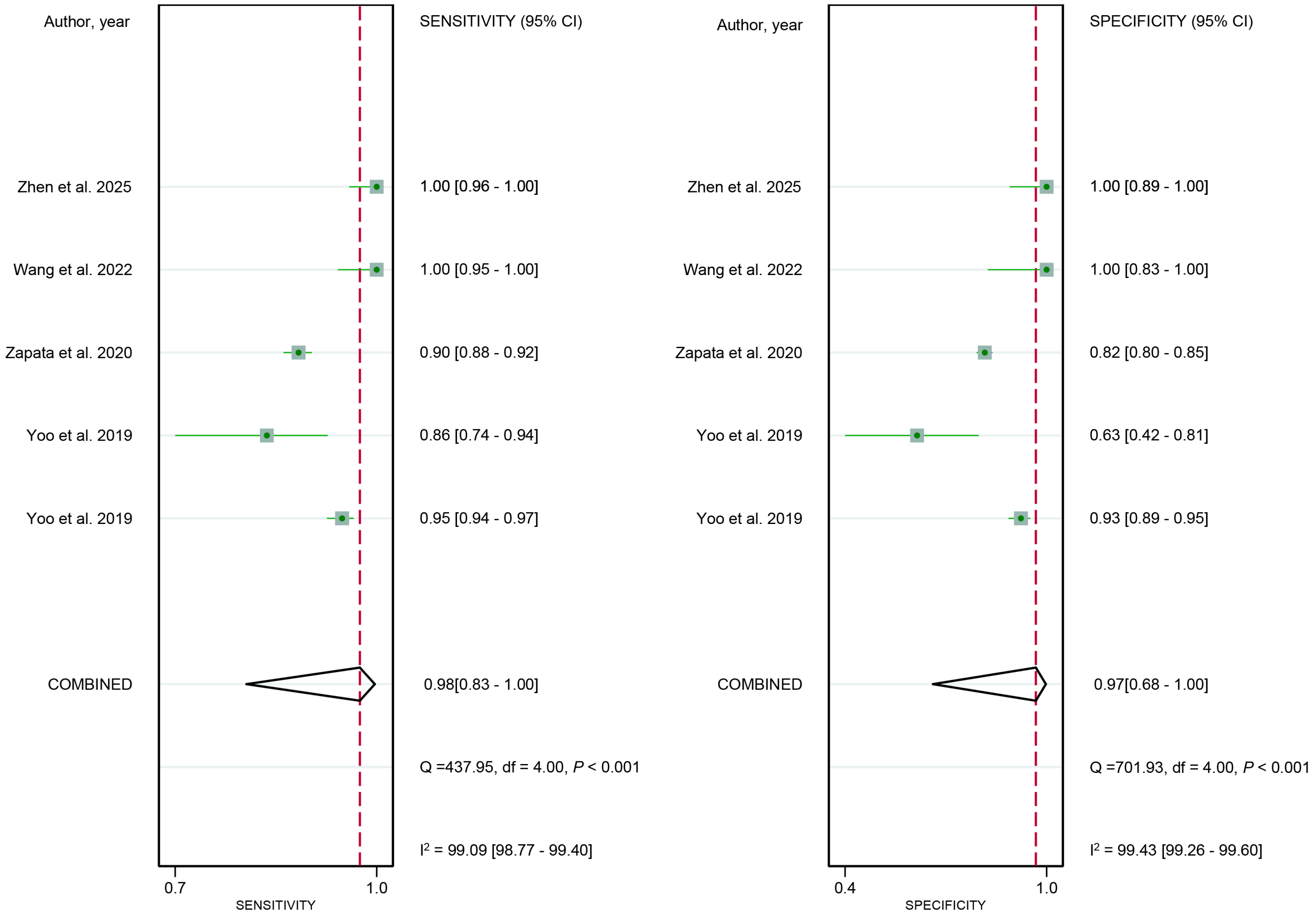

Figure S16. Forest plots of the pooled accuracy of deep learning algorithms using generalized linear mixed model (GLMM) for classifying age-related macular degeneration (AMD) from normal based on multimodal images.

Author, year	Events	Total	GLMM, Accuracy, 95% CI
Zhen et al. 2025	134	134	1.00 [0.97 - 1.00]
Wang et al. 2022	91	91	1.00 [0.96 - 1.00]
Zapata et al. 2020	1899	2208	0.86 [0.84 - 0.87]
Yoo et al. 2019	851	900	0.95 [0.93 - 0.96]
Yoo et al. 2019	65	83	0.78 [0.68 - 0.87]
**Total (95% CI)**		**3416**	**0.98 [0.78 - 1.00]**

Heterogeneity: $Tau^2 = 5.1780$; $Chi^2 = 48.93$, df = 4 (P < 0.0001); $I^2 = 91.8\%$

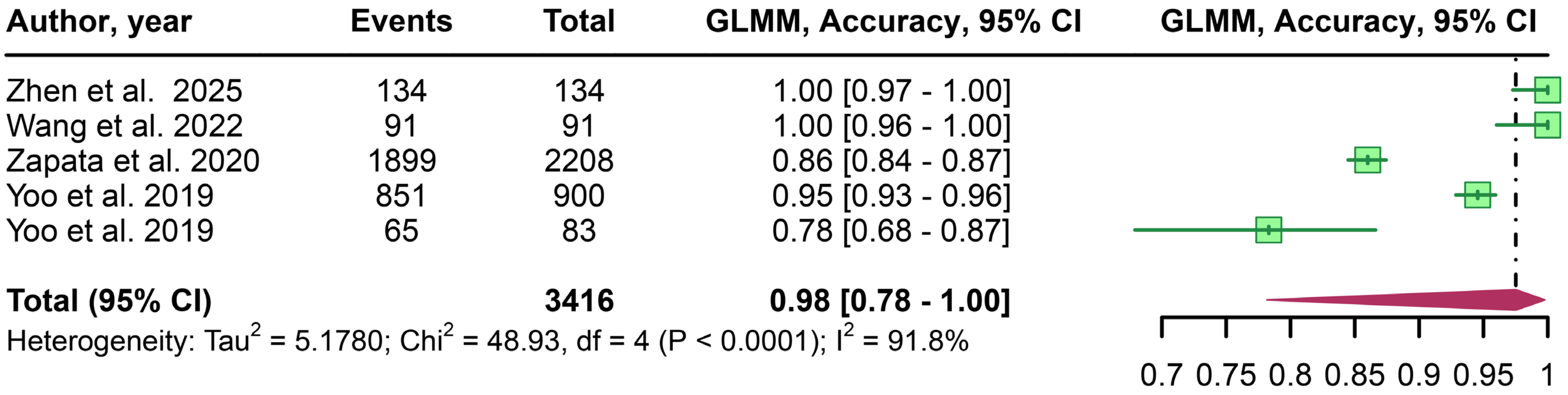

Figure S17. Summary receiver operating characteristic (SROC) curve of deep learning algorithms for classifying age-related macular degeneration (AMD) from normal based on multimodal images.

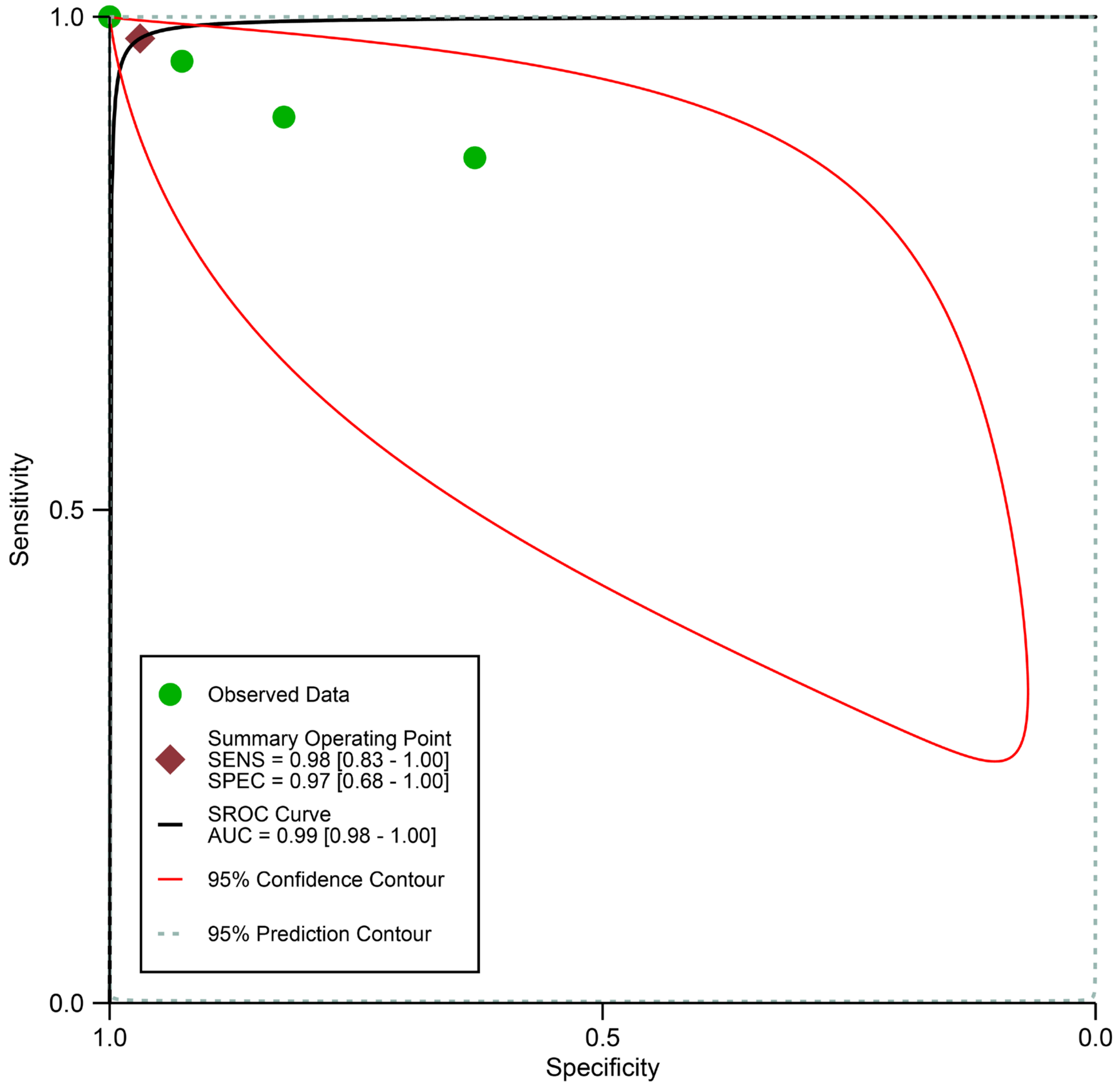

Figure S18. Forest plots of the pooled sensitivity and specificity of deep learning algorithms for classifying wet age-related macular degeneration (wAMD) from dry age-related macular degeneration (dAMD) based on optical coherence tomography (OCT) images.

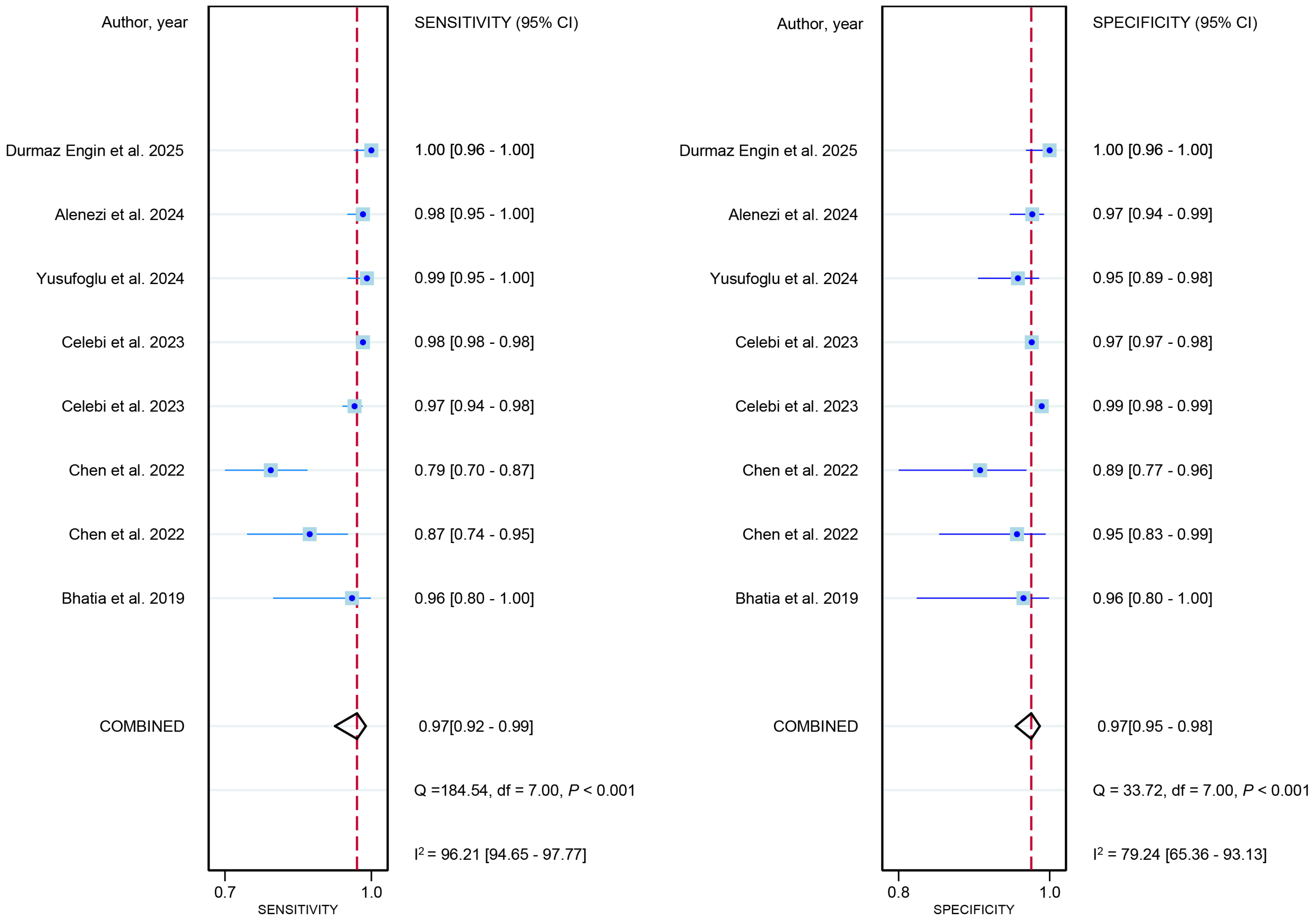

Figure S19. Forest plots of the pooled accuracy of deep learning algorithms using generalized linear mixed model (GLMM) for classifying wet age-related macular degeneration (wAMD) from dry age-related macular degeneration (dAMD) based on optical coherence tomography (OCT) images.

Author, year	Events	Total	GLMM, Accuracy, 95% CI
Durmaz Engin et al. 2025	200	200	1.00 [0.98 - 1.00]
Alenezi et al. 2024	353	361	0.98 [0.96 - 0.99]
Yusufoglu et al. 2024	207	213	0.97 [0.94 - 0.99]
Celebi et al. 2023	1064	1085	0.98 [0.97 - 0.99]
Celebi et al. 2023	17006	17369	0.98 [0.98 - 0.98]
Chen et al. 2022	79	87	0.91 [0.83 - 0.96]
Chen et al. 2022	118	143	0.83 [0.75 - 0.88]
Bhatia et al. 2019	48	50	0.96 [0.86 - 1.00]
**Total (95% CI)**		**19508**	**0.97 [0.94 - 0.99]**

Heterogeneity: $Tau^2 = 1.0988$; $Chi^2 = 119.08$, df = 7 ($P < 0.0001$); $I^2 = 94.1\%$

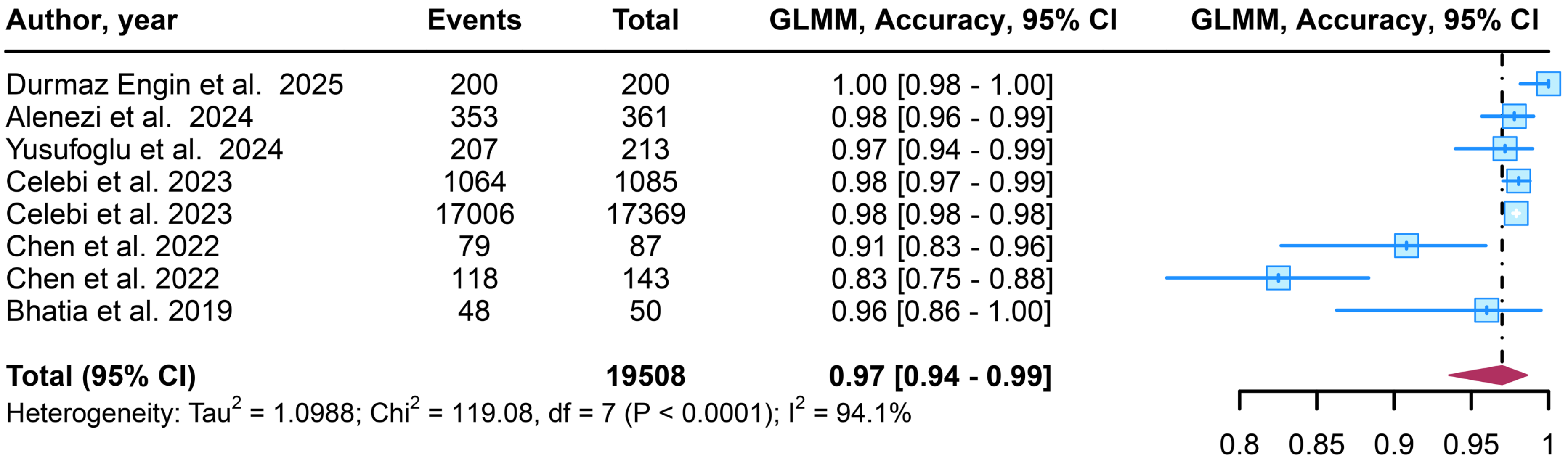

Figure S20. Summary receiver operating characteristic (SROC) curve of deep learning algorithms for classifying wet age-related macular degeneration (wAMD) from dry age-related macular degeneration (dAMD) based on optical coherence tomography (OCT) images.

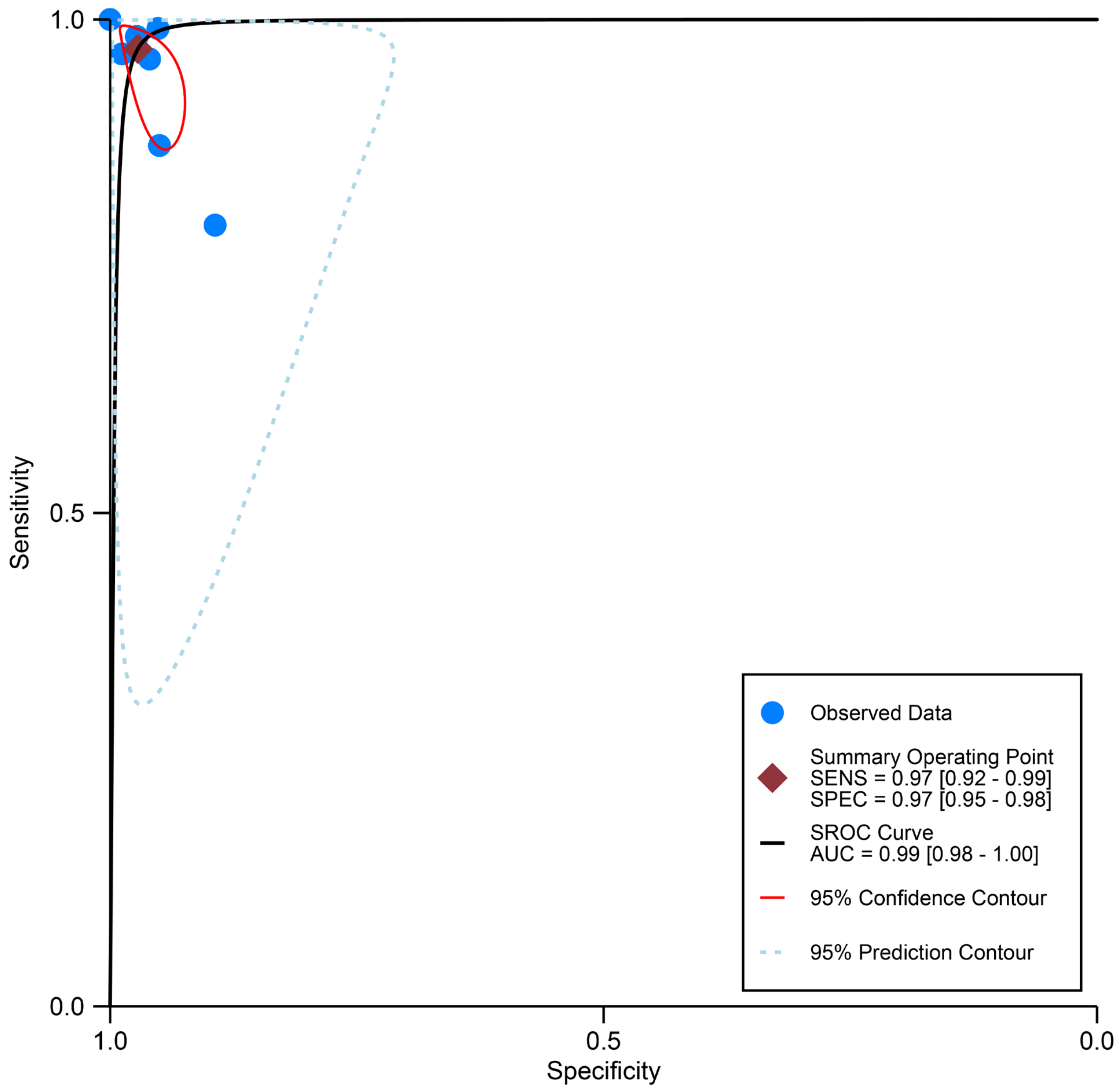

Figure S21. Forest plots of the pooled sensitivity and specificity of deep learning algorithms for classifying wet age-related macular degeneration (wAMD) from dry age-related macular degeneration (dAMD) based on color fundus photography (CFP) images.

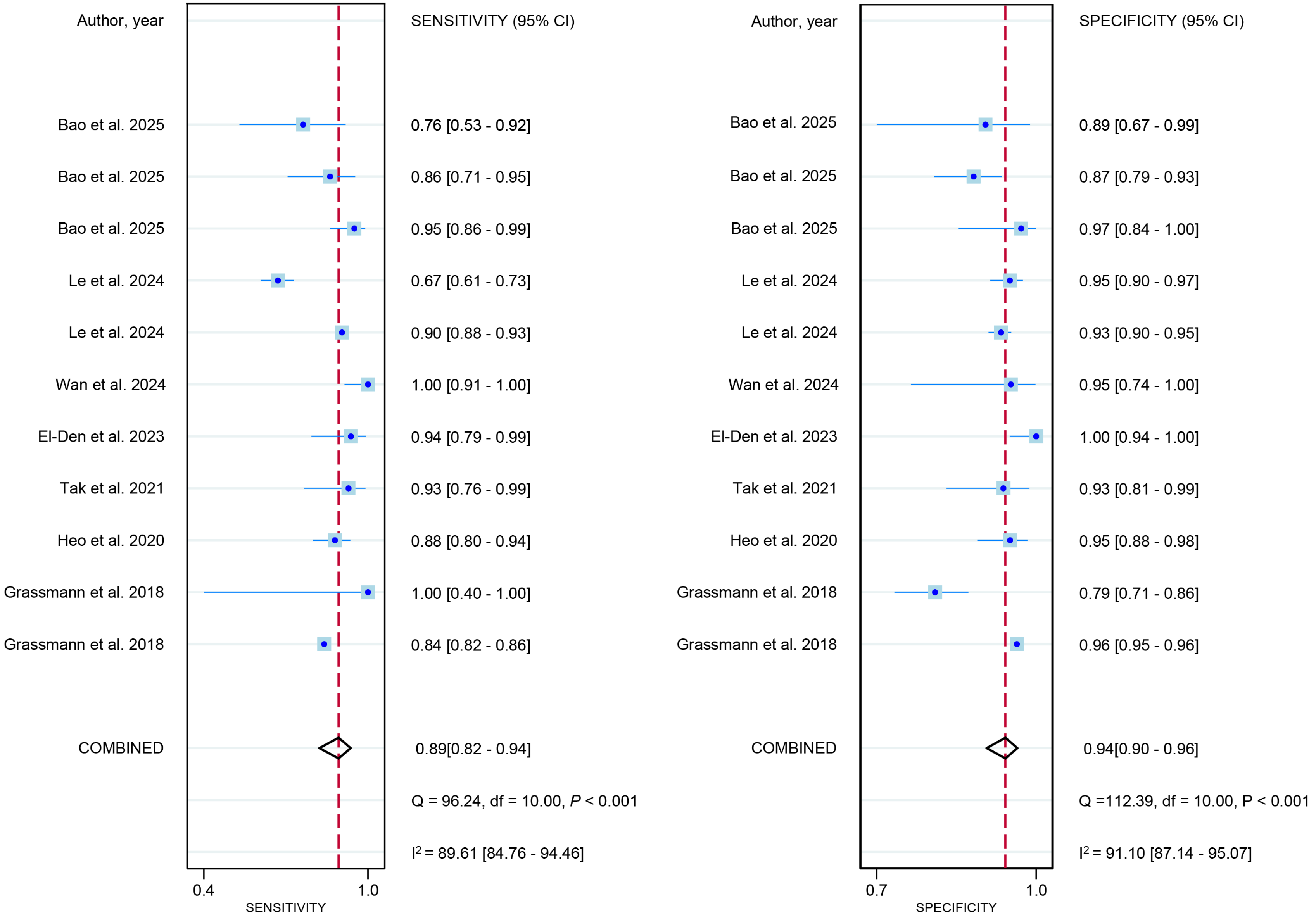

Figure S22. Forest plots of the pooled accuracy of deep learning algorithms using generalized linear mixed model (GLMM) for classifying wet age-related macular degeneration (wAMD) from dry age-related macular degeneration (dAMD) based on color fundus photography (CFP) images.

Author, year	Events	Total	GLMM, Accuracy, 95% CI
Bao et al. 2025	88	92	0.96 [0.89 - 0.99]
Bao et al. 2025	118	136	0.87 [0.80 - 0.92]
Bao et al. 2025	33	40	0.82 [0.67 - 0.93]
Le et al. 2024	1002	1095	0.92 [0.90 - 0.93]
Le et al. 2024	350	440	0.80 [0.75 - 0.83]
Wan et al. 2024	59	60	0.98 [0.91 - 1.00]
El−Den et al. 2023	95	97	0.98 [0.93 - 1.00]
Tak et al. 2021	67	72	0.93 [0.85 - 0.98]
Heo et al. 2020	174	191	0.91 [0.86 - 0.95]
Grassmann et al. 2018	6192	6631	0.93 [0.93 - 0.94]
Grassmann et al. 2018	98	123	0.80 [0.71 - 0.86]
**Total (95% CI)**		**8977**	**0.91 [0.87 - 0.94]**

Heterogeneity: $Tau^2 = 0.4440$; $Chi^2 = 141.37$, df = 10 (P < 0.0001); $I^2 = 92.9\%$

GLMM, Accuracy, 95% CI

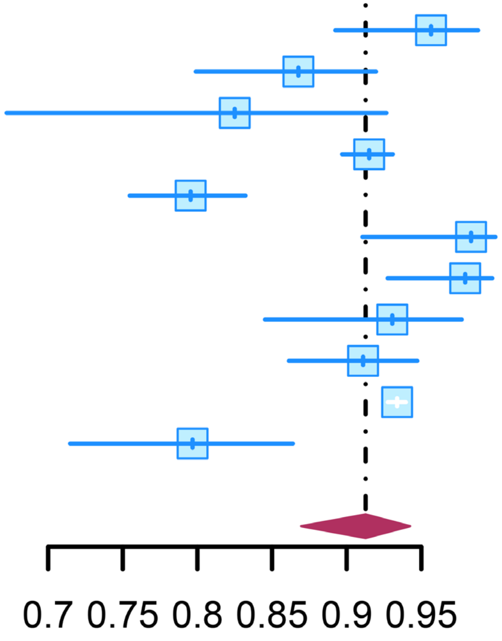

Figure S23. Summary receiver operating characteristic (SROC) curve of deep learning algorithms for classifying wet age-related macular degeneration (wAMD) from dry age-related macular degeneration (dAMD) based on color fundus photography (CFP) images.

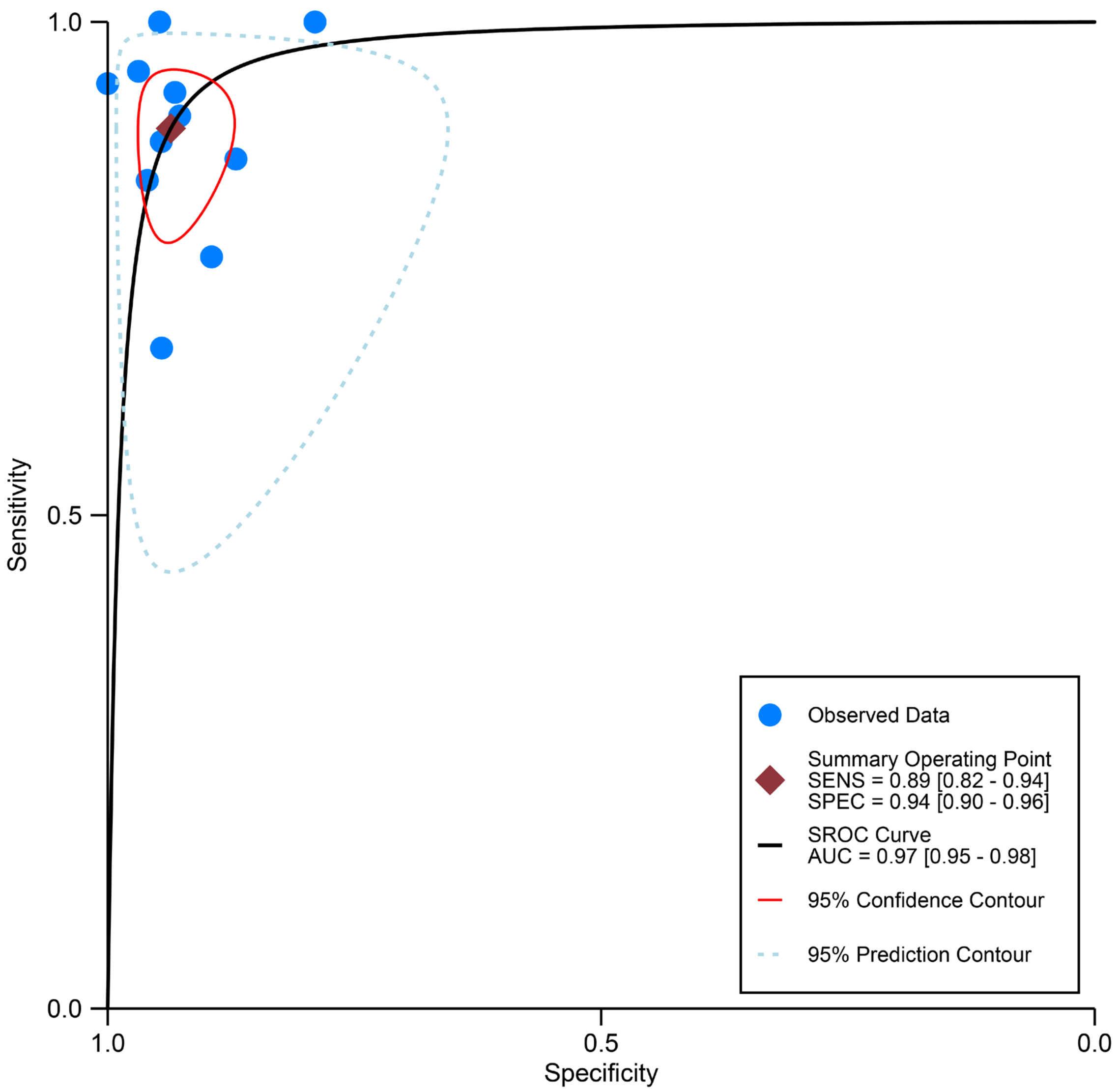

Figure S24. Forest plots of the pooled sensitivity of deep learning algorithms for classifying wet age-related macular degeneration (wAMD) from dry age-related macular degeneration (dAMD) based on multimodal images.

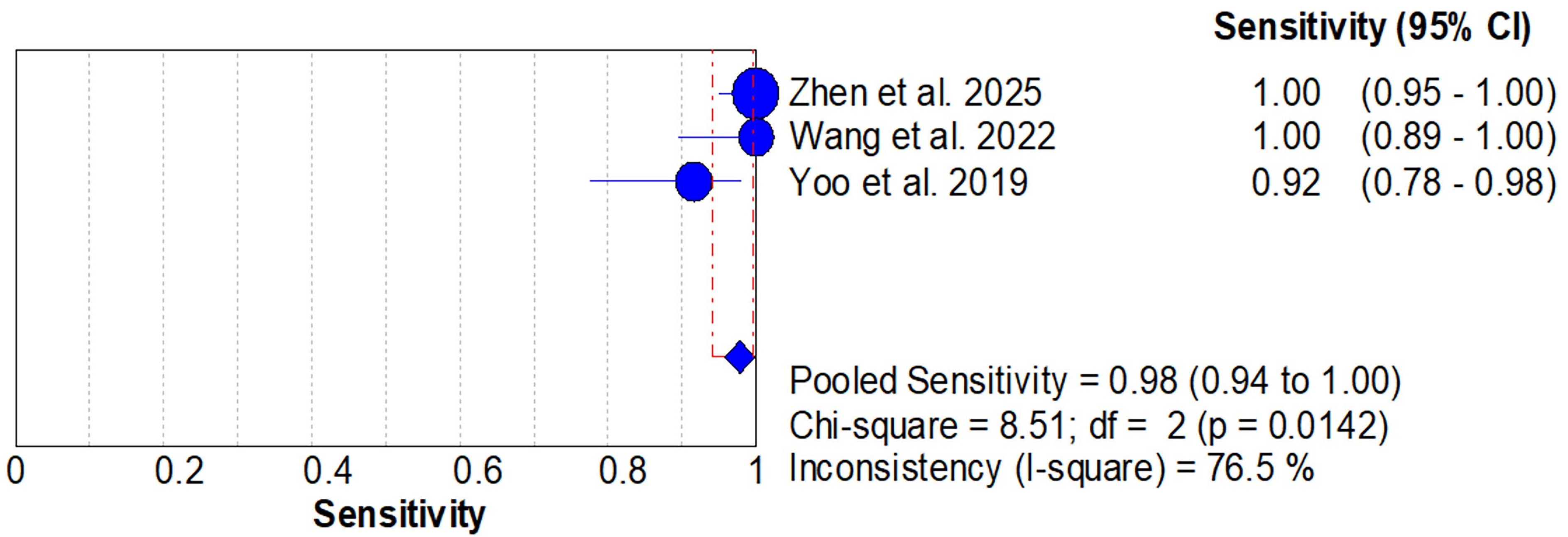

Figure S25. Forest plots of the pooled specificity of deep learning algorithms for classifying wet age-related macular degeneration (wAMD) from dry age-related macular degeneration (dAMD) based on multimodal images.

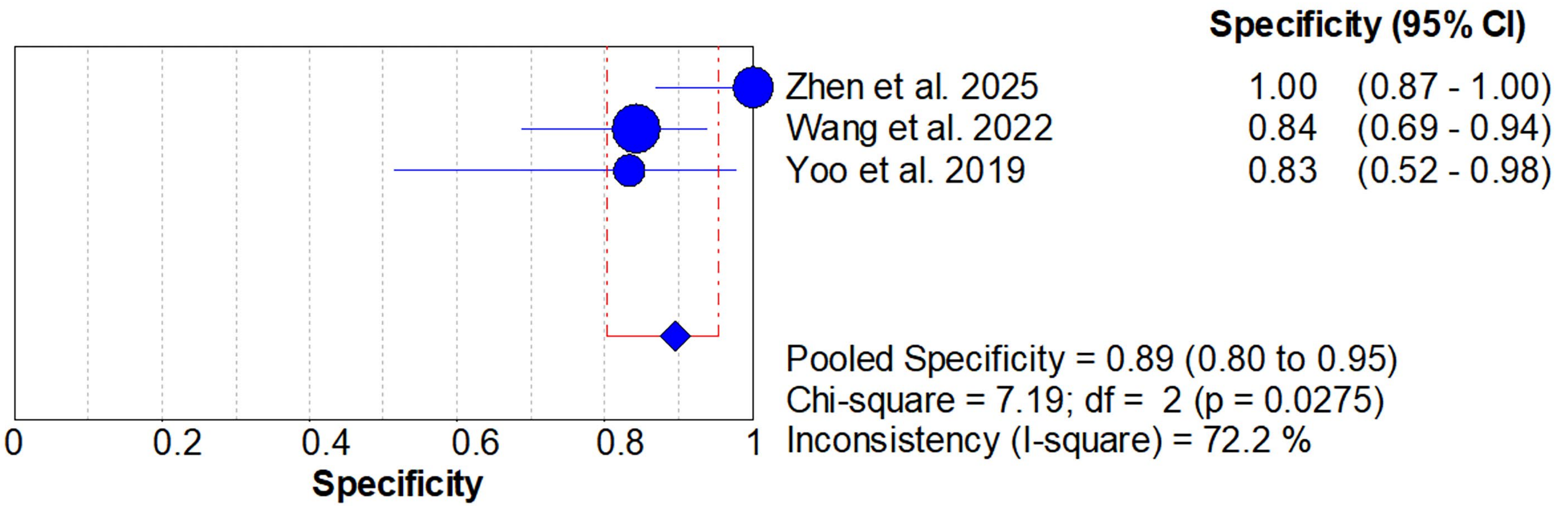

Figure S26. Forest plots of the pooled accuracy of deep learning algorithms using generalized linear mixed model (GLMM) for classifying wet age-related macular degeneration (wAMD) from dry age-related macular degeneration (dAMD) based on multimodal images.

Author, year	Events	Total	GLMM, Accuracy, 95% CI
Zhen et al. 2025	101	101	1.00 [0.96 - 1.00]
Wang et al. 2022	65	71	0.92 [0.83 - 0.97]
Yoo et al. 2019	43	48	0.90 [0.77 - 0.97]
**Total (95% CI)**		**220**	**0.96 [0.80 - 0.99]**

Heterogeneity: $Tau^2 = 1.9065$; $Chi^2 = 0.13$, df = 2 (P = 0.9364); $I^2 = 0.0\%$

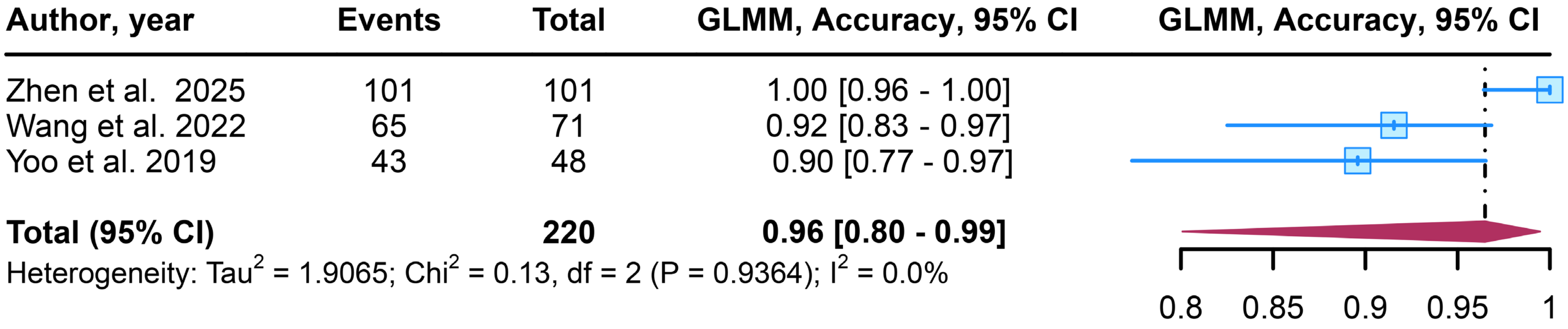

Figure S27. Summary receiver operating characteristic (SROC) curve of deep learning algorithms for classifying wet age-related macular degeneration (wAMD) from dry age-related macular degeneration (dAMD) based on multimodal images.

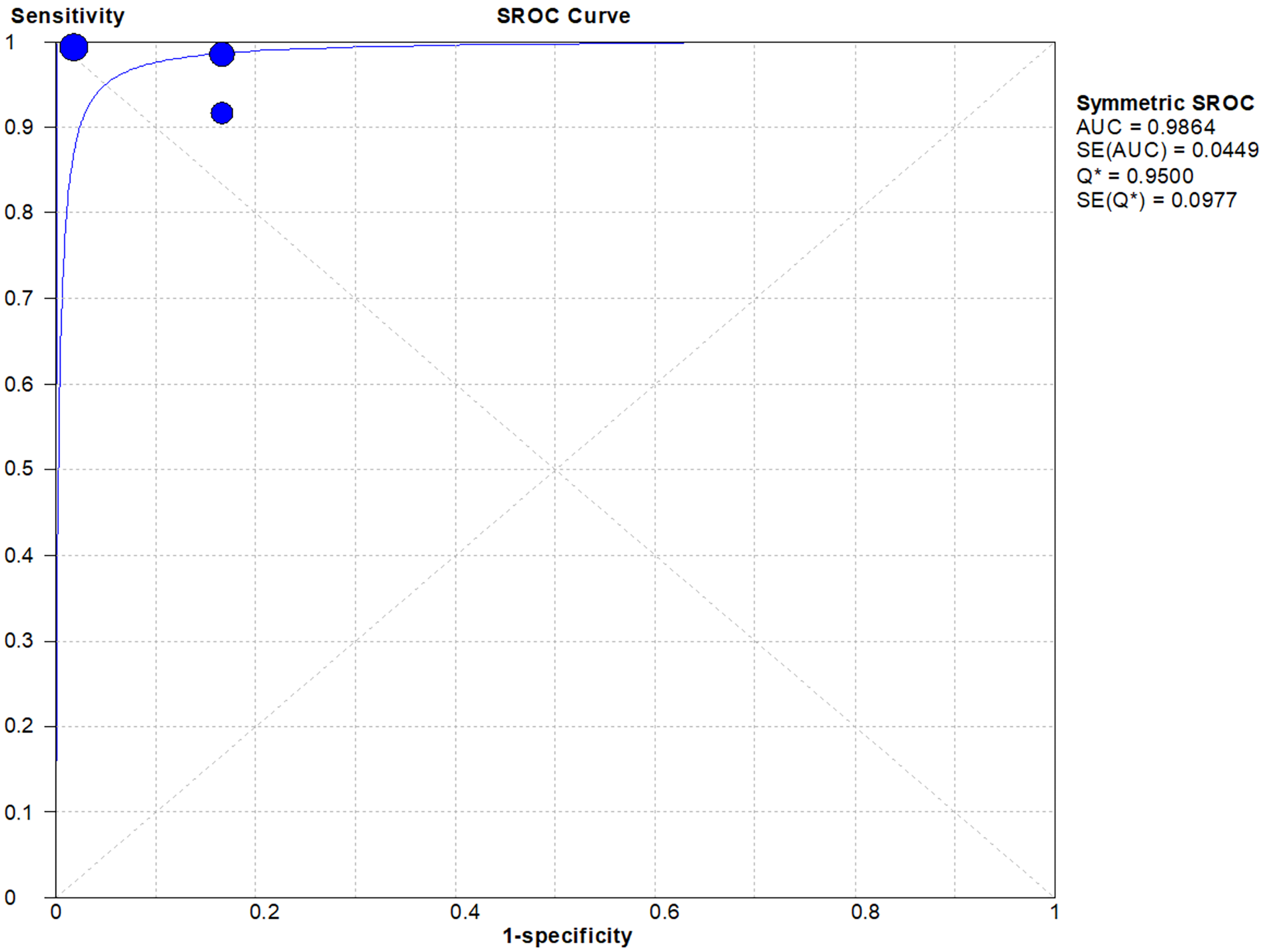

References

1. Abdelhalim I, Nadmid N, Elsharkawy M, Ghazal M, Mahmoud AH, El-Baz A. Mask-UnMask Regions (MUMR) Framework for Classifying AMD Grades Using Inter-Regional Interaction Analysis. IEEE ACCESS. 2025;13:8286-96. PMID: WOS:001397807300020. doi: 10.1109/ACCESS.2025.3526948.

2. Bao S, Yang Z, Zhang Z, Qu J, Sun J. AttResAMD: An Attention-Driven Deep Learning Framework for Expert-Level Automated Classification of Age-Related Macular Degeneration from Fundus Photography. Interdiscip Sci. 2025 Aug 30. PMID: 40885885. doi: 10.1007/s12539-025-00763-x.

3. Durmaz Engin C, Beşenk U, Özizmirliler D, Selver MA. Comparative Analysis of Automated vs. Expert-Designed Machine Learning Models in Age-Related Macular Degeneration Detection and Classification. Turk J Ophthalmol. 2025 Jun 25;55(3):120-6. PMID: 40560103. doi: 10.4274/tjo.galenos.2025.74780.

4. Zhen B, Qi Y, Tang Z, Liu C, Zhao S, Yu Y, et al. Low-Rank Fine-Tuning Meets Cross-modal Analysis: A Robust Framework for Age-Related Macular Degeneration Categorization. J Imaging Inform Med. 2025 Apr 29. PMID: 40301288. doi: 10.1007/s10278-025-01513-7.

5. Alenezi A, Alhamad H, Brindhaban A, Amizadeh Y, Jodeiri A, Danishvar S. Enhancing Readability and Detection of Age-Related Macular Degeneration Using Optical Coherence Tomography Imaging: An AI Approach. Bioengineering (Basel). 2024 Mar 22;11(4). PMID: 38671722. doi: 10.3390/bioengineering11040300.

6. García-Floriano A, Ventura-Molina E. Age-Related Macular Degeneration Detection in Retinal Fundus Images by a Deep Convolutional Neural Network. MATHEMATICS. 2024 MAY;12(10). PMID: WOS:001231500000001. doi: 10.3390/math12101445.

7. Le NT, Truong TL, Deelertpaiboon S, Srisiri W, Pongsachareonnont PF, Suwajanakorn D, et al. ViT-AMD: A New Deep Learning Model for Age-Related Macular Degeneration Diagnosis From Fundus Images. INTERNATIONAL JOURNAL OF INTELLIGENT SYSTEMS. 2024 NOV 15;2024. PMID: WOS:001362656600001. doi: 10.1155/2024/3026500.

8. Oliveira GC, Rosa GH, Pedronette DCG, Papa JP, Kumar H, Passos LA, et al. Robust deep learning for eye fundus images: Bridging real and synthetic data for enhancing generalization. Biomedical Signal Processing and Control. 2024;94. doi: 10.1016/j.bspc.2024.106263.

9. Wan C, Zhao JN, Hong XQ, Yang WH, Zhang SC. HCSP-Net: A Novel Model of Age-Related Macular Degeneration Classification Based on Color Fundus Photography. CMC-COMPUTERS MATERIALS & CONTINUA. 2024;79(1):391-407. PMID: WOS:001225035600018. doi: 10.32604/cmc.2024.048307.

10. Yusufoğlu E, Fırat H, Üzen H, Özçelik STA, Çiçek İ B, Şengür A, et al. A Comprehensive CNN Model for Age-Related Macular Degeneration Classification Using OCT: Integrating Inception Modules, SE Blocks, and ConvMixer. Diagnostics (Basel). 2024 Dec 17;14(24). PMID: 39767197. doi: 10.3390/diagnostics14242836.

11. Celebi ARC, Bulut E, Sezer A. Artificial intelligence based detection of age-related macular degeneration using optical coherence tomography with unique image preprocessing. Eur

J Ophthalmol. 2023 Jan;33(1):65-73. PMID: 35469472. doi: 10.1177/11206721221096294.

12. El-Den NN, Naglah A, Elsharkawy M, Ghazal M, Alghamdi NS, Sandhu H, et al. Scale-adaptive model for detection and grading of age-related macular degeneration from color retinal fundus images. Sci Rep. 2023 Jun 13;13(1):9590. PMID: 37311794. doi: 10.1038/s41598-023-35197-2.

13. Leingang O, Riedl S, Mai J, Reiter GS, Faustmann G, Fuchs P, et al. Automated deep learning-based AMD detection and staging in real-world OCT datasets (PINNACLE study report 5). Sci Rep. 2023 Nov 9;13(1):19545. PMID: 37945665. doi: 10.1038/s41598-023-46626-7.

14. Chen M, Jin K, Yan Y, Liu X, Huang X, Gao Z, et al. Automated diagnosis of age-related macular degeneration using multi-modal vertical plane feature fusion via deep learning. Med Phys. 2022 Apr;49(4):2324-33. PMID: 35172022. doi: 10.1002/mp.15541.

15. He T, Zhou Q, Zou Y. Automatic Detection of Age-Related Macular Degeneration Based on Deep Learning and Local Outlier Factor Algorithm. Diagnostics (Basel). 2022 Feb 18;12(2). PMID: 35204621. doi: 10.3390/diagnostics12020532.

16. Skevas C, Weindler H, Levering M, Engelberts J, van Grinsven M, Katz T. Simultaneous screening and classification of diabetic retinopathy and age-related macular degeneration based on fundus photos—a prospective analysis of the RetCAD system. International Journal of Ophthalmology. 2022;15(12):1985.

17. Wang W, Li X, Xu Z, Yu W, Zhao J, Ding D, et al. Learning Two-Stream CNN for Multi-Modal Age-Related Macular Degeneration Categorization. IEEE J Biomed Health Inform. 2022 Aug;26(8):4111-22. PMID: 35503853. doi: 10.1109/jbhi.2022.3171523.

18. Tak N, Reddy AJ, Martel J, Martel JB. Clinical Wide-Field Retinal Image Deep Learning Classification of Exudative and Non-Exudative Age-Related Macular Degeneration. Cureus. 2021 Aug;13(8):e17579. PMID: 34646633. doi: 10.7759/cureus.17579.

19. Takhchidi HP, Gliznitsa PV, Svetozarskiy SN, Bursov AI, Shusterzon KA. Labelling of data on fundus color pictures used to train a deep learning model enhances its macular pathology recognition capabilities. Bulletin of Russian State Medical University. 2021 (4):28-33. doi: 10.24075/brsmu.2021.040.

20. Thomas A, Harikrishnan PM, Gopi VP, Palanisamy P. AN AUTOMATED METHOD to DETECT AGE-RELATED MACULAR DEGENERATION from OPTICAL COHERENCE TOMOGRAPHIC IMAGES. Biomedical Engineering - Applications, Basis and Communications. 2021;33(5). doi: 10.4015/S1016237221500368.

21. Heo T-Y, Kim KM, Min HK, Gu SM, Kim JH, Yun J, et al. Development of a deep-learning-based artificial intelligence tool for differential diagnosis between dry and neovascular age-related macular degeneration. Diagnostics. 2020;10(5):261.

22. Zapata MA, Royo-Fibla D, Font O, Vela JI, Marcantonio I, Moya-Sánchez EU, et al. Artificial Intelligence to Identify Retinal Fundus Images, Quality Validation, Laterality

Evaluation, Macular Degeneration, and Suspected Glaucoma. Clin Ophthalmol. 2020;14:419-29. PMID: 32103888. doi: 10.2147/opth.S235751.

23. Bhatia KK, Graham MS, Terry L, Wood A, Tranos P, Trikha S, et al. DISEASE CLASSIFICATION OF MACULAR OPTICAL COHERENCE TOMOGRAPHY SCANS USING DEEP LEARNING SOFTWARE: Validation on Independent, Multicenter Data. Retina. 2020 Aug;40(8):1549-57. PMID: 31584557. doi: 10.1097/iae.0000000000002640.

24. Matsuba S, Tabuchi H, Ohsugi H, Enno H, Ishitobi N, Masumoto H, et al. Accuracy of ultra-wide-field fundus ophthalmoscopy-assisted deep learning, a machine-learning technology, for detecting age-related macular degeneration. Int Ophthalmol. 2019 Jun;39(6):1269-75. PMID: 29744763. doi: 10.1007/s10792-018-0940-0.

25. Yoo TK, Choi JY, Seo JG, Ramasubramanian B, Selvaperumal S, Kim DW. The possibility of the combination of OCT and fundus images for improving the diagnostic accuracy of deep learning for age-related macular degeneration: a preliminary experiment. Med Biol Eng Comput. 2019 Mar;57(3):677-87. PMID: 30349958. doi: 10.1007/s11517-018-1915-z.

26. Grassmann F, Mengelkamp J, Brandl C, Harsch S, Zimmermann ME, Linkohr B, et al. A Deep Learning Algorithm for Prediction of Age-Related Eye Disease Study Severity Scale for Age-Related Macular Degeneration from Color Fundus Photography. Ophthalmology. 2018 Sep;125(9):1410-20. PMID: 29653860. doi: 10.1016/j.ophtha.2018.02.037.

27. Tan JH, Bhandary SV, Sivaprasad S, Hagiwara Y, Bagchi A, Raghavendra U, et al. Age-related Macular Degeneration detection using deep convolutional neural network. FUTURE GENERATION COMPUTER SYSTEMS-THE INTERNATIONAL JOURNAL OF ESCIENCE. 2018 OCT;87:127-35. PMID: WOS:000437997500010. doi: 10.1016/j.future.2018.05.001.

28. Lee CS, Baughman DM, Lee AY. Deep Learning Is Effective for Classifying Normal versus Age-Related Macular Degeneration OCT Images. OPHTHALMOLOGY RETINA. 2017 JUL-AUG;1(4):322-7. PMID: WOS:000662245800015. doi: 10.1016/j.oret.2016.12.009.
