# Supplementary material for: Performance of Deep Learning in Classifying Age-Related Macular Degeneration From Images: Systematic Review and Meta-Analysis
Source: J Med Internet Res. 2026 Jun 15;28:e97174. doi: 10.2196/97174 (PMC13268637; doi:10.2196/97174)
Supplement: Checklist 1 [file jmir-v28-e97174-s002.pdf]

PRISMA checklists

Performance of Deep Learning in Classifying Age-related Macular Degeneration from Images: Systematic Review and Meta-analysis

Table of Contents

Table S1. Preferred Reporting Items for Systematic Reviews and Meta-Analyses of Diagnostic Test Accuracy (PRISMA-DTA) Checklist......3

Table S2. PRISMA 2020 for Abstracts Checklist. 5

Table S1. Preferred Reporting Items for Systematic Reviews and Meta-Analyses of Diagnostic Test Accuracy (PRISMA-DTA) Checklist.

TITLE/ABSTRACT			Reported on page #
Title	1	Identify the report as a systematic review (+/−meta-analysis) of diagnostic test accuracy (DTA) studies	1
Abstract	2	See the PRISMA for Abstracts checklist.	4-5
**INTRODUCTION**			
Rationale	3	Describe the rationale for the review in the context of what is already known	6-8
Clinical role of index test	D1	State the scientific and clinical background, including the intended use and clinical role of the index test, and if applicable, the rationale for minimally acceptable test accuracy (or minimum difference in accuracy for comparative design)	6-8
Objectives	4	Provide an explicit statement of question(s) being addressed in terms of participants, index test(s), and target condition(s)	8
**METHODS**			
Protocol and registration	5	Indicate if a review protocol exists, if and where it can be accessed (e.g., Web address), and, if available, provide registration information including registration number.	8
Eligibility criteria	6	Specify study characteristics (participants, setting, index test(s), reference standard(s), target condition(s), and study design) and report characteristics (e.g., years considered, language, publication status) used as criteria for eligibility, giving rationale.	9, 39
Information sources	7	Describe all information sources (e.g., databases with dates of coverage, contact with study authors to identify additional studies) in the search and date last searched.	8-9
Search	8	Present full search strategies for all electronic databases and other sources searched, including any limits used, such that they could be repeated.	8-9
Selection process	9	State the process for selecting studies (i.e., screening, eligibility, included in systematic review, and, if applicable, included in the meta-analysis).	8-9
Data collection process	10	Describe method of data extraction from reports (e.g., piloted forms, independently, in duplicate) and any processes for obtaining and confirming data from investigators.	10
Definitions for data extraction	11	Provide definitions used in data extraction and classifications of target condition(s), index test(s), reference standard(s) and other characteristics (e.g., study design, clinical setting).	10-11, 39
Risk of bias and applicability	12	Describe methods used for assessing risk of bias in individual studies and concerns regarding the applicability to the review question.	9-10
Diagnostic accuracy measures	13	State the principal diagnostic accuracy measure(s) reported (e.g., sensitivity, specificity) and state the unit of assessment (e.g., per-patient, per-lesion).	10-11, 39
Synthesis of results	12	Describe methods of handling data, combining results of studies and describing variability between studies. This could include, but is not limited to: a) handling of multiple definitions of target condition. b) handling of multiple thresholds of test positivity, c) handling multiple<br>index test readers, d) handling of indeterminate test results, e) grouping and comparing tests, f) handling of different reference standards	11-12
Meta-analysis	D2	Report the statistical methods used for meta-analyses, if performed.	11-12
Additional analyses	16	Describe methods of additional analyses (e.g., sensitivity or subgroup analyses, meta-regression), if done, indicating which were pre-specified.	11-12
**RESULTS**			

Study selection	17	Provide numbers of studies screened, assessed for eligibility, included in the review (and included in meta-analysis, if applicable) with reasons for exclusions at each stage, ideally with a flow diagram.	12-13
Study characteristics	18	For each included study provide citations and present key characteristics including: a) participant characteristics (presentation, prior testing), b) clinical setting, c) study design, d) target condition definition, e) index test, f) reference standard, g) sample size, h) funding sources	13-14, 40-43
Risk of bias in studies	19	Present evaluation of risk of bias and concerns regarding applicability for each study.	14-15
Results of individual studies	20	For each analysis in each study (e.g., unique combination of index test, reference standard, and positivity threshold) report 2x2 data (TP, FP, FN, TN) with estimates of diagnostic accuracy and confidence intervals, ideally with a forest or receiver operator characteristic (ROC) plot.	15-19
Synthesis of results	22	Describe test accuracy, including variability; if meta-analysis was done, include results and confidence intervals.	15-23, 44-45
Additional analysis	23	Give results of additional analyses, if done (e.g., sensitivity or subgroup analyses, meta-regression; analysis of index test: failure rates, proportion of inconclusive results, adverse events).	19-23
**DISCUSSION**			
Summary of evidence	24	Summarize the main findings including the strength of evidence.	15, 24-25, 44-45
Limitations	25	Discuss limitations from included studies (e.g., risk of bias and concerns regarding applicability) and from the review process (e.g., incomplete retrieval of identified research).	30
Conclusions	26	Provide a general interpretation of the results in the context of other evidence. Discuss implications for future research and clinical practice (e.g., the intended use and clinical role of the index test).	31
**FUNDING**			
Funding	26	For the systematic review, describe the sources of funding and other support and the role of the funders.	2

Salameh, Jean-Paul et al. "Preferred reporting items for systematic review and meta-analysis of diagnostic test accuracy studies (PRISMA-DTA): explanation, elaboration, and checklist." *BMJ (Clinical research ed.)* vol. 370 m2632. 14 Aug. 2020, doi:10.1136/bmj.m2632

Table S2. PRISMA 2020 for Abstracts Checklist.

**Section and Topic**	**Item #**	**Checklist item**	**Reported on page #**
**TITLE**			
Title	1	Identify the report as a systematic review.	1
**BACKGROUND**			
Objectives	2	Provide an explicit statement of the main objective(s) or question(s) the review addresses.	4
**METHODS**			
Eligibility criteria	3	Specify the inclusion and exclusion criteria for the review.	4
Information sources	4	Specify the information sources (e.g. databases, registers) used to identify studies and the date when each was last searched.	4
Risk of bias	5	Specify the methods used to assess risk of bias in the included studies.	4
Synthesis of results	6	Specify the methods used to present and synthesise results.	4
**RESULTS**			
Included studies	7	Give the total number of included studies and participants and summarise relevant characteristics of studies.	5
Synthesis of results	8	Present results for main outcomes, preferably indicating the number of included studies and participants for each. If meta-analysis was done, report the summary estimate and confidence/credible interval. If comparing groups, indicate the direction of the effect (i.e. which group is favoured).	5
**DISCUSSION**			
Limitations of evidence	9	Provide a brief summary of the limitations of the evidence included in the review (e.g. study risk of bias, inconsistency and imprecision).	5
Interpretation	10	Provide a general interpretation of the results and important implications.	5
**OTHER**			
Funding	11	Specify the primary source of funding for the review.	2
Registration	12	Provide the register name and registration number.	4
